# Antileishmanial compounds from *Connarus suberosus*: Metabolomics, isolation and mechanism of action

**DOI:** 10.1371/journal.pone.0241855

**Published:** 2020-11-06

**Authors:** Lais S. Morais, Renata G. Dusi, Daniel P. Demarque, Raquel L. Silva, Lorena C. Albernaz, Sônia N. Báo, Christian Merten, Luciana M. R. Antinarelli, Elaine S. Coimbra, Laila S. Espindola

**Affiliations:** 1 Laboratório de Farmacognosia, Universidade de Brasília, Campus Universitário Darcy Ribeiro, Asa Norte, Brasília, DF, Brazil; 2 Fakultät für Chemie und Biochemie, Organische Chemie II, Ruhr-Universität Bochum, Bochum, Germany; 3 Laboratório de Microscopia e Microanálise, Instituto de Ciências Biológicas, Universidade de Brasília, Campus Universitário Darcy Ribeiro, Asa Norte, Brasília, DF, Brazil; 4 Departamento de Parasitologia, Microbiologia e Imunologia, I.C.B., Universidade Federal de Juiz de Fora, Campus Universitário Juiz de Fora, Minas Gerais, Brazil; Institute for Biological Research "S. Stanković", University of Belgrade, SERBIA

## Abstract

Leishmaniasis is a disease impacting public health worldwide due to its high incidence, morbidity and mortality. Available treatments are costly, lengthy and toxic, not to mention the problem of parasite resistance. The development of alternative treatments is warranted and natural products demonstrate promising activity. This study investigated the activity of *Connarus suberosus* extracts and compounds against *Leishmania* species. Several *C*. *suberosus* extracts were tested against *L*. *amazonensis* promastigotes. Active and inactive extracts were analyzed by UHPLC-MS and data evaluated using a metabolomics platform, revealing an unknown neoflavonoid (connarin, **3**), isolated together with the pterocarpans: hemileiocarpin (**1**) and leiocarpin (**2**). The aforementioned compounds (**1**–**3**), together with the benzoquinones: rapanone (**4**), embelin (**5**) and suberonone (**6**) previously isolated by our group from the same species, were tested against: (i) *L*. *amazonensis* and *L*. *infantum* promastigotes, and (ii) *L*. *amazonensis* intracellular amastigotes, with the most active compound (**3**) also tested against *L*. *infantum* amastigotes. Cytotoxicity against murine peritoneal macrophages was also investigated. Compounds **2** and **3** presented an IC_50_ 33.8 μM and 11.4 μM for *L*. *amazonensis* promastigotes; and 44.3 μM and 13.3 μM for *L*. *infantum* promastigotes, respectively. For *L*. *amazonensis* amastigotes, the IC_50_ of **2** was 20.4 μM with a selectivity index (SI) of 5.7, while the IC_50_ of **3** was 2.9 μM with an SI of 6.3. For *L*. *infantum* amastigotes, the IC_50_ of **3** was 7.7 μM. Compounds **2** and **3** presented activity comparable with the miltefosine positive control, with compound **3** found to be 2–4 times more active than the positive control, depending on the *Leishmania* species and form. The extracts and isolated compounds showed moderate toxicity against macrophages. Compounds **2** and **3** altered the mitochondrial membrane potential (ΔΨm) and neutral lipid body accumulation, while **2** also impacted plasma membrane permeabilization, culminating in cellular disorder and parasite death. Transmission electron microscopy of *L*. *amazonensis* promastigotes treated with compound **3** confirmed the presence of lipid bodies. Leiocarpin (**2**) and connarin (**3**) demonstrated antileishmanial activity. This study provides knowledge of natural products with antileishmanial activity, paving the way for prototype development to fight this neglected tropical disease.

## Introduction

Leishmaniasis is a vector-borne disease caused by a variety of protozoan species of the *Leishmania* genus. This Neglected Tropical Disease is endemic to 92 countries and is associated with socioeconomic factors, with Brazil accounting for 84% of the cases reported in the Americas [1]. Approximately 1 billion people live in endemic regions, with annual estimates of 1 million cutaneous leishmaniasis and 90,000 visceral leishmaniasis cases [2, 3]. Current treatment consists of only a few therapeutic options, with various limitations associated with prolonged use, parenteral administration, toxicity and parasite resistance [4–6]. Therefore, the development of prototype drugs is important, with natural products (NP) demonstrating promising activity against different parasites. The complexity of NP metabolism can result in the formation of complex chemical structures that can be used as lead molecules for new medicines. A study of 74 publications (2008–2018) reported a total of 86 isolated active compounds against the *Leishmania* genus [7]. A source of taxonomically diverse species and active compounds is the Cerrado, the second largest biome in Brazil. Widely distributed in this biome, *Connarus* constitutes the principal genus of the Connaraceae family [8–10]. Our research group reported a *C*. *suberosus* extract with activity against several disease-causing agents, including *L*. *amazonensis* promastigotes [9]. In this study, the highly abundant compounds isolated from the root bark of this plant were not previously reported for their antileishmanial activity, warranting further in-depth investigation.

The exploration of active plant compounds usually relies on traditional bio-guided isolation studies. However, with the significant efforts of natural product chemists exploring the chemical diversity of plants over the last decades, chemical studies with plants can result in the re-isolation of known compounds. In order to avoid this scenario, modern analytical and statistical analysis, allied to the use of databases, has been applied in metabolomics analysis to rapidly recognize known and unidentified compounds in complex matrices, thus resulting in a more efficient drug discovery process [11]. Therefore, the present study used a metabolomics approach to investigate the antileishmanial compounds of *C*. *suberosus* extracts. This strategy considered both biological and chemical data to identify compounds potentially responsible for the activity, thus guiding the isolation process. Analysis resulted in the isolation of a new compound in active fractions. Biological tests confirmed its activity and its mechanism of action was determined, together with the other compounds described herein.

## Results and discussion

**Fig 1:**
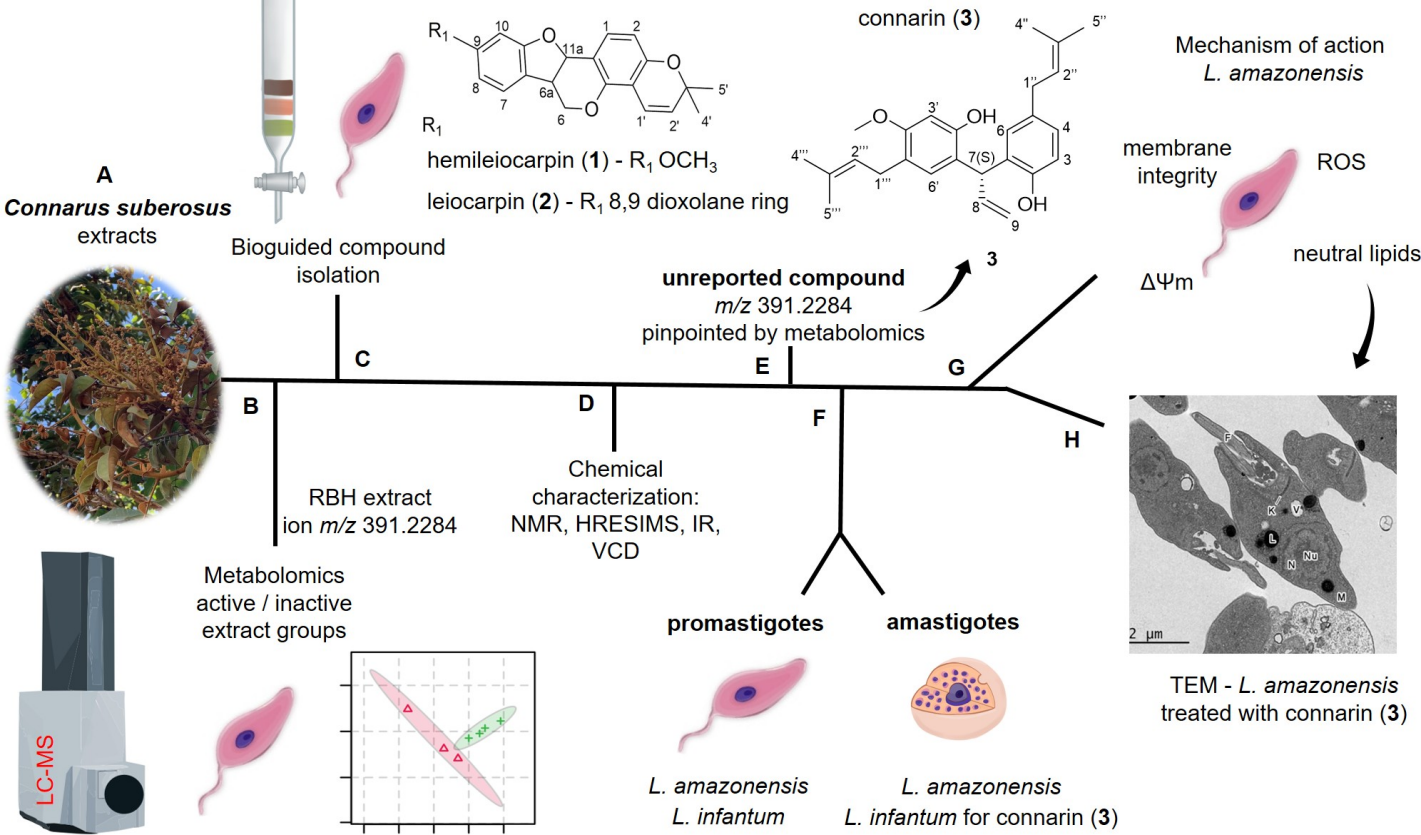
Flow diagram of the chemical and biological steps to identify active compounds in *Connarus suberosus*. (A) 9 different extracts from Brazilian Cerrado Biome Plant Extract Bank. (B) statistical grouping of extracts using chemical and biological data, with the RBH (root bark hexane) extract containing ion m/z 391.2284, important for differentiation within the active group. (C) Classical isolation in *L*. *amazonensis* promastigotes. (D) NMR (nuclear magnetic resonance), HRESIMS (high resolution electrospray ionization mass spectrometry), IR (infrared), and VCD (vibrational circular dichroism). (E) 3 isolated compounds. (F) Extract and compound activity. (G) Compound **2** and **3** studies: ROS (reactive oxygen species) production, ΔΨm (mitochondrial membrane potential) and neutral lipid body accumulation. (H) TEM (transmission electron microscopy).

Nine *C*. *suberosus* (Connaraceae) extracts were tested against *L*. *amazonensis* promastigotes at 100 μg/mL and 50 μg/mL (S1 Table). Three extracts were considered active, inhibiting parasite multiplication by >80%. LC-MS data of the 9 active and inactive extracts was considered in the statistical analysis. Ion *m/z* 391.2284 was important for differentiation between active and inactive samples. The calculated molecular formulae C_26_H_32_O_3_-H^+^ [M-H]^-^ (error = 2.8 ppm) did not match any compound isolated in the family according to the SciFinder database. The *C*. *suberosus* root bark hexane extract was the most active and had the aforementioned ion pinpointed by metabolomics. Classical bio-guided fractionation of the extract culminated in the isolation of connarin (**3**), an unreported compound with the same molecular mass as the ion pinpointed by metabolomics, and two pterocarpans: hemileiocarpin (**1**) and leiocarpin (**2**). Compound **3** was identified using traditional techniques and vibrational circular dichroism (VCD). Compounds **1**, **2**, **3** and the benzoquinones: raponone (**4**), suberonone (**5**) and embelin (**6**), previously isolated by our group [12], were tested against *L*. *infantum* and *L*. *amazonensis* promastigotes and *L*. *amazonensis* amastigotes. Compounds **2** and **3** presented an IC_50_ 33.8 μM and 11.4 μM for *L*. *amazonensis*; and 44.3 μM and 13.3 μM for *L*. *infantum*, promastigotes respectively. For *L*. *amazonensis* amastigotes, the IC_50_ of compound **2** was 20.4 μM with a selectivity index (SI) 5.7, while the IC_50_ of compound **3** was 2.9 μM (SI 6.3). Connarin (**3**), the most active compound, was also tested against *L*. *infantum* amastigotes with an IC_50_ 7.7 μM (SI 2.4). Compounds **2** and **3** presented activity comparable to the miltefosine positive control (Table 3) and were therefore selected to study the mechanism of action in *L*. *amazonensis* promastigotes. Both compounds reduced the mitochondrial membrane potential and increased neutral lipid bodies. Transmission eletron microscopy confirmed the high number of lipid bodies produced in *L*. *amazonensis* promastigotes after treatment with compound **3**. Fig 1 presents the combined chemical and biological tools to isolate antileishmanial compounds.

### Screening and metabolomic analysis of *C*. *suberosus* crude extracts

Liquid chromatography coupled with mass spectrometry (LC-MS) is a powerful tool that can be used in dereplication and data analysis [13, 14]. By assessing chemical formulae, it provides a metabolite fingerprint and uses chemometric techniques to give a better overview of the data. Our research group reported that comparison of metabolite fingerprints obtained from the LC-MS data of active and inactive samples by means of multivariate analysis can elucidate their differences and, therefore, reveal the potentially active compounds [11]. In this regard, a total of 9 *C*. *suberosus* extracts prepared: 3 hexane (H) extracts—root bark (RBH), stem wood (SWH) and root wood (RWH); 5 ethyl acetate (EtOAc) extracts–RBEtOAc, SWEtOAc, RWEtOAc, leaf (LEtOAc) and stem bark (SBEtOAc); and 1 ethanol (EtOH) extract–RWEtOH. All 9 extracts were tested against *L*. *amazonensis* promastigotes, with 3 extracts demonstrating mortality at 100 μg/mL: RBH (89.2%), SWH (86.0%) and RWEtOAc (82.3%) (S1 Table). These screening results support different extract chemical profiles. According to the data presented in S1 Table, ethyl acetate was a more effective solvent than hexane and ethanol for extracting active compounds from the root wood. Prior to the isolation procedures, untargeted metabolomic analysis was performed using LC-MS data from active and inactive extracts against *L*. *amazonensis* promastigotes (100 μg/mL). Multivariate analysis was conducted in order to annotate the metabolic differences between active and inactive extracts (see methods for details, “Metabolomics” section) [15].

**Fig 2:**
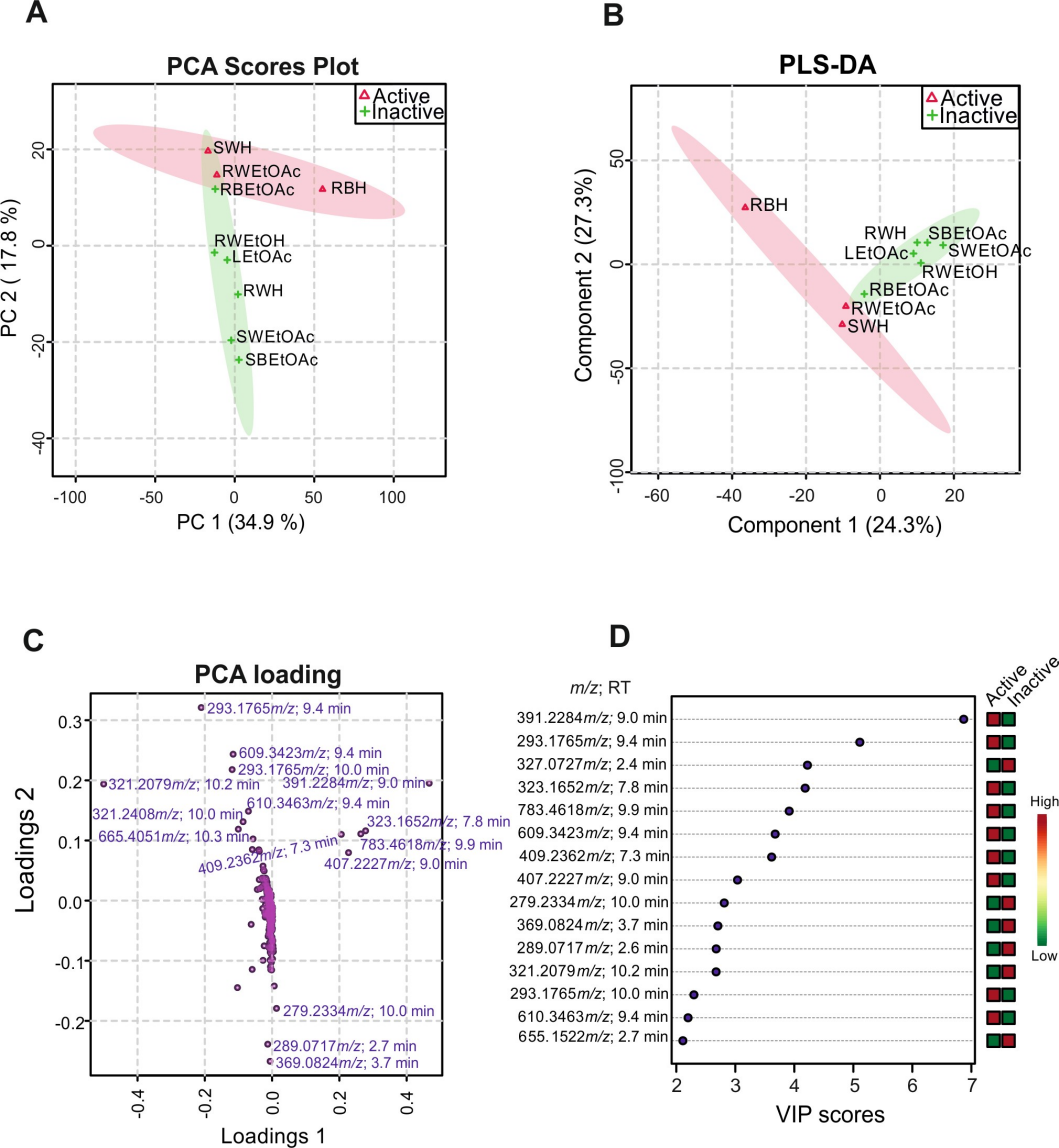
Statistical analysis produced with metabolomics data. (A) PCA (principal component analysis) does not consider differences between active and inactive samples to group them, (**Fig** B) PLS-DA (partial least squares discriminant analysis) considers the difference and maximizes it, (C) PCA–loading, and (D) VIP (Variable importance projection) scores show the different important ions presented in the chemical profile of 9 different *C*. *suberosus* extracts used to create active and inactive groups, both pinpointing ion *m/z* 391.2284 as important for differentiation, and associated *L*. *amazonensis* promastigote mortality.

Fig 2 summarizes the metabolomics data generated using the MetaboAnalyst platform. Supervised and unsupervised analyses were carried out to identify metabolic differences between active/inactive samples. As an unsupervised analysis, the PCA score does not consider the different features between the two groups (active/inactive) to build the scores plot. In Fig 2(A), it was possible to recognize two distinct groups accounting for 52.7% of the data variability of active and inactive *C*. *suberosus* extracts. The PCA loading (Fig 2C) shows several ions that could be used to distinguish the two groups.

Considering that in unsupervised analysis the intersection between the confidence regions of active and inactive samples may indicate that differences are delicate, we also performed supervised analysis, which considers the existence of 2 groups and maximizes their differences. The PLS-DA graphic obtained from this analysis (Fig 2B) represents 51.6% of the data and shows a negligible intersection. Fig 2(D) (VIP scores) shows ions with high importance in group differentiation of PLS-DA, most of which were also present among the PCA score (Fig 2C). Therefore, based on a better separation of the active and inactive extracts, we considered the 15 most important ions ranked in VIP scores to perform the annotation.

VIP scores shows the ion *m/z* 391.2284, corresponding to the molecular formula C_26_H_32_O_3_-H^+^ [M-H]^-^ (error = 2.8 ppm), with the highest score. A search in the SciFinder database resulted in no identification. Its dimer also appears among the 15 most important ions (*m/z* 783.4644). The ion *m/z* 293.1765 appears twice, with a small retention time variation, and was identified as embelin (C_17_H_26_O_4_-H^+^ [M-H]^-^), which has its antileishmanial activity reported [16]. The ions *m/z* 609.3423 and *m/z* 610.3463 relate to embelin, as the sodiated dimer and sodiated dimer isotope, respectively. The ions *m/z* 323.1652, *m/z* 409.2362 and *m/z* 407.2227 presented low intensity and were not identified. Among the most important ions for group differentiation present in inactive samples, we identified rapanone *m/z* 321.2079 (C_19_H_30_O_4_-H^+^ [M-H]^-^). The presence of this compound in inactive samples is in accordance with a previous *C*. *suberosus* study conducted by our group, that reported a rapanone/suberonone mixture inactive against *L*. *amazonensis* promastigotes [9]. The other ions were not identified.

### Isolation of compounds

#### Isolation and identification of the compound with the highest VIP score

**Fig 3:**
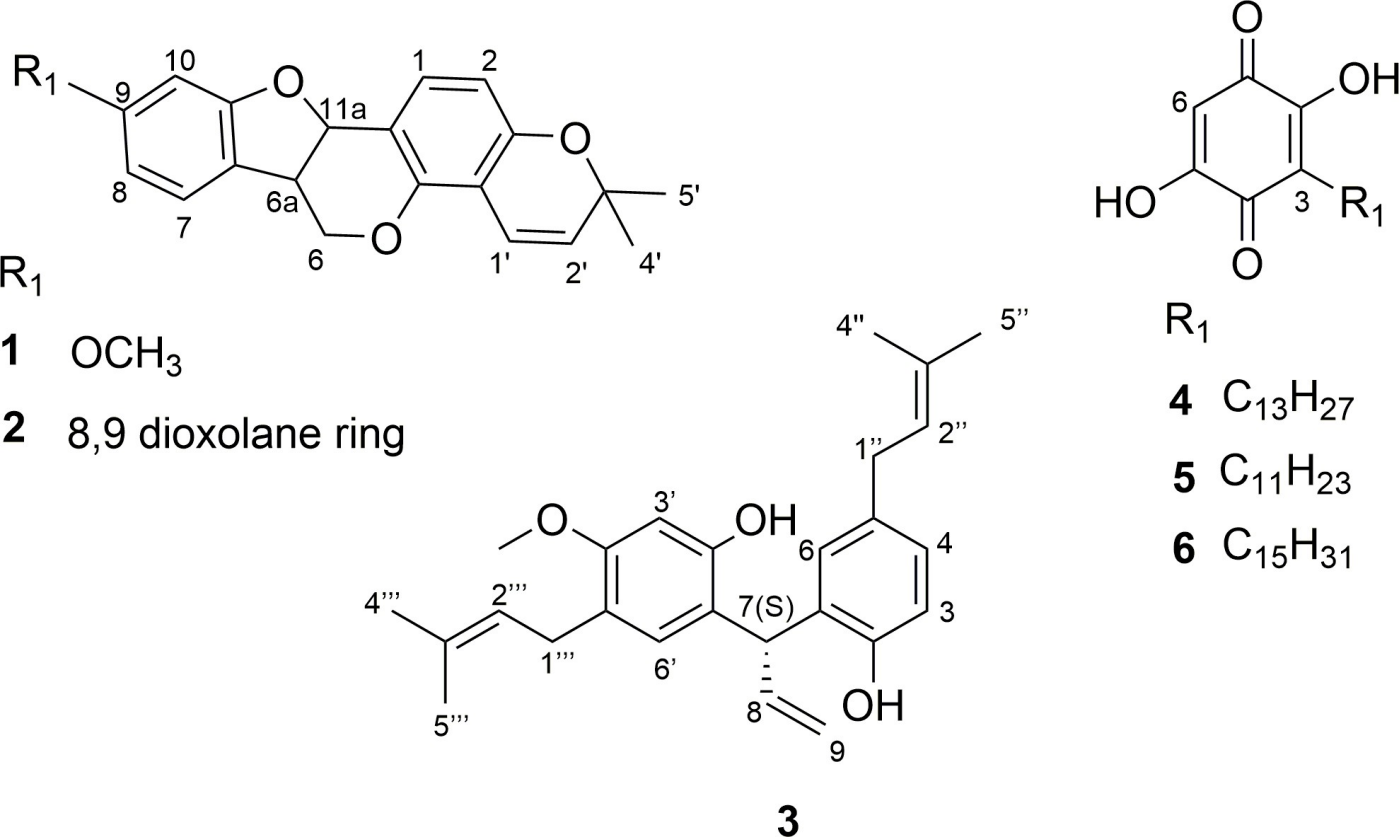
Isolated compounds from *C*. *suberosus*. *C*. *suberosus* compounds: hemileiocarpin (**1**) and leiocarpin (**2**), isolated due to the simple fraction chromatographic profiles; connarin (**3**), the ion pinpointed by metabolomics as important for activity, not found in the SciFinder database; and the quinones: rapanone (**4**), embelin (**5**) and suberonone (**6**) previously isolated by our group [12].

The RBH extract, containing the most active, and unknown compound was submitted to SPE diol fractionation using five different solvents: hexane/CH_2_Cl_2_ (9:1—**A**), CH_2_Cl_2_/EtOAc (20:1—**B**), EtOAc (**C**), EtOAc/MeOH (5:1—**D**) and MeOH (**E**). Fractions were tested against *L*. *amazonensis* promastigotes, with SPE fractions **A** and **B** presenting similar activity to the aforementioned extract (Table 1). Due to its activity (IC_50_ 12.1 μg/mL) and yield, Fraction **A** was submitted to a silica column, resulting in 29 (A1-A29) fractions. The highest yielding fraction (A22), together with the five adjacent upstream and downstream fractions (A17-A27), were tested against *L*. *amazonensis* promastigotes with 24 h exposure at 100 μg/mL (S1A Fig). Fraction A22 was the most active with 91.1% inhibition and was analyzed by LC-MS, with the major ion the same as that pinpointed by metabolomic analysis (*m/z* 391.2284; C_26_H_32_O_3_-H^+^ [M-H]^-^). Therefore, A22 was fractionated in a Sephadex LH-20 column, yielding compound **3** (Fig 3), with an IC_50_ 20.5 μM (CI_95_ 18.0 to 23.8 μM) against *L*. *amazonensis* promastigotes after 24 h of exposure (S1B Fig). This result confirmed the metabolomics results.

**Table 1:** Activity of SPE-Diol fractions from *C*. *suberosus* Root Bark Hexane (RBH) extract against *L*. *amazonensis* promastigotes, after 24 h.

Sample	^a^IC_50_ (μg/mL) ^b^(CI_95_)
RBH extract	13.9 (11.6–16.8)
Fraction A	12.1 (10.5–13.9)
Fraction B	11.7 (9.5–14.3)
Fraction C	22.3 (20.3–24.6)
Fraction D	116.5 (103.6–131.0)
Fraction E	> 250
^c^Amphotericin B	< 7.8

^a^Concentration necessary for 50% inhibition.

^b^95% confidence interval. A—hexane:CH_2_Cl_2_ (9:1); B—CH_2_Cl_2_:EtOAc (20:1); C–EtOAc; D—EtOAc:MeOH (5:1), and E -MeOH.

^c^Positive control

Compound **3** was isolated as a previously unreported pale yellow-green oil neoflavonoid, named herein as connarin. In HRESIMS, connarin (**3**) showed a [M+Na]^+^ ion at *m/z* 415.2265 (C_26_H_32_O_3_+Na^+^_,_ error = -3.8 ppm). The IR spectra showed signals at 3300 cm^-1^ (OH), 2912 cm^-1^ (aliphatic chains), 1504 cm^-1^ (aromatic) and 1200 cm^-1^ (ether group). The ^1^H NMR (600 MHz, CDCl_3_) showed four singlets in the aliphatic regions (δ_H_ 1.6 to δ_H_ 1.8; C-4”, C-5” and C-4”‘, C-5”‘) corresponding to four methyl groups, and a methoxy (δ_H_ 3.68; C-4’a). The methyl groups are the terminal part of the isoprene units that are substituents of aromatic rings. Two broad singlets were observed at δ_H_ 5.7 - δ_H_ 6.0 (C-1 and C-2’), corresponding to phenolic hydroxyls. Signals in the olefin region (δ_H_ 5.09 - δ_H_ 5.31; C-2”, C2”‘, C-9) correspond to unsaturation of the isoprene units and unsaturation of the link between the two aromatic rings possessing a deshielded signal at δ_H_ 6.35 (C-8). Five aromatic hydrogens suggest the presence of two rings with seven substitutions (C-3, C-4, C6, C-3’and C-6’). The ^13^C spectra presented 26 signals, corroborating the molecular formula of the ion in the mass spectrum. No carbonyl signals were detected, confirming that the three oxygen atoms were in the form of two hydroxyl groups and a methoxy. The edited HSQC spectrum shows the presence of three CH_2_ signals, two from the isoprene units (C-2” and C-2”‘) and a diastereotopic at δ_C_ 117.0 (C-9), deshielded since it is located near the chiral carbon at δ_C_ 42.0 (C-7). It is possible to see different coupling constants for the protons of δ_C_ 117.0 (C-9) due to their different interaction with δ_H_ 6.35 (C-8). The spectra showed five CH_3_ (including the methoxy group), three CH_2_ (described above), nine CH, and nine non-protonated carbons. HMBC shows correlation of hydrogens from two different rings with the carbon δ_C_ 42.0 (C-7), suggesting it is the link between the two rings. The NMR data and selected HMBC correlations are presented in Table 2 and S18 Fig, respectively. The absolute configuration (spatial arrangement of atoms) was determined as (7S) by vibrational circular dichroism (VCD) spectroscopy [17, 18]. This absolute configuration result is important as it impacts biological activity [19]. The presence of a chiral carbon creates different possibilities for the position of substituents in three dimensions [20]. The spectra are available in S2 Fig.

**Table 2:** ^1^H (600 MHz) and ^13^C (150 MHz) NMR data assignments for compound 3– chemical shifts (ppm) and coupling constants.

Position	δC	δH *(*J in Hz)
1	118.6	
2	151.0	
3	116.2	6.74 d (8.2)
4	127.7	6.93 dd (2.2,8.2)
5	134.7	
6	128.8	6.97 d (2.2)
7	42.0	5.01 m
8	138.6	6.35 m
9	117.0	5.09 dt (17.2, 1.5)
9	117.0	5.31 dt (10.3, 1.5)
1'	128.2	
2'	152.1	
3'	99.7	6.31 s
4'	157.0	
4'a	55.3	3.68 s
5'	122.7	
6'	129.5	6.84 bs
1''	33.6	3.24 d (7.3)
2''	123.5	5.26 m
3''	132.3	
4''	17.8	1.68 bs
5''	25.7	1.73 m
1‴	27.9	3.17 m
2‴	122.8	5.21 m
3‴	132.2	
4‴	25.7	1.69 m
5‴	17.7	1.64 bs

#### Other compounds investigated

Due to the low complexity of the chromatographic profiles of A10 and A11 fractions eluted from the silica column, they were submitted to PTLC (preparative thin layer chromatography) fractionation, and yielded hemileiocarpin (**1**) and leiocarpin (**2**), respectively (Fig 3). These two pterocarpans were identified by comparing NMR and HRESIMS data with the literature [21–23]. In a previous study, our research group isolated three benzoquinones from the *C*. *suberosus* root wood ethyl acetate extract, also investigated herein: rapanone (**4**), embelin (**5**) and suberonone (**6**) [12] (Fig 3).

### Prolonged antileishmanial activity exposure of extracts and isolated compounds

After the preliminary screening (24 h–*L*. *amazonensis* promastigote assays), crude extracts and the aforementioned compounds (**1**–**6**) were submitted to prolonged exposure tests over 72 h at several concentrations. Two *Leishmania* species were used in the promastigote tests: *L*. *amazonensis*, classically associated with cutaneous leishmaniasis, and *L*. *infantum*, mainly related to fatal visceral manifestation. Due to the epidemiological importance of *L*. *amazonensis* regarding cutaneous cases, wide distribution of the species in Brazil and ability to cause several forms of the disease [1, 24, 25], all compounds were assayed against its amastigote form. In addition, cytotoxicity tests were also conducted in murine peritoneal macrophages (Table 3 and S2 Table).

**Table 3:** *In vitro* antileishmanial activity, cytotoxicity in murine peritoneal macrophages, and selectivity index of a *C*. *suberosus* Root Bark Hexane (RBH) extract and compounds 1–6.

	Promastigotes ^a^IC_50_				Amastigotes IC_50_		Cytotoxicity ^b^CC_50_		
Sample	*L*. *amazonensis*		*L*. *infantum*		*L*. *amazonensis*		Murine macrophages		^c^SI
	μg/mL	μM	μg/mL	μM	μg/mL	μM	μg/mL	μM	
**1**	^**d**^nt	nt	20.8± 8.5	61.9 ± 25.6	10.6 (8.5–13.4)	31.6 (25.2–39.8)	>100	>297	^**e**^-
**2**	11.4 ± 3.5	33.8 ±10.1	15.5 ± 4.8	44.3 ± 13.6	7.1 (6.2–8.1)	20.4 (17.8–23.2)	39.0 (33.3–45.7)	116.0 (99.0–135.9)	5.7
**3**	4.5 ± 0.5	11.4 ± 1.2	5.2 ± 0.9	13.3 ± 2.4	1.13 (0.9–1.4)	2.9 (2.4–4.1)	7.2 (5.4–9.6)	18.3 (13.8–24.3)	6.3
**4**	50.4 ± 4.6	169.1 ± 4.6	7.17 ± 0.3	24.5 ± 1.0	>3.2	>10.6	2.9 (2.2–3.9)	9.8 (7.4–12.9)	-
**5**	12.5 ± 0.9	38.9 ± 2.7	10.7 ± 0.7	33.2 ± 2.0	>3.2	>9.7	0.9 (0.7–1.1)	2.6 (2.0–3.4)	-
**6**	51.9 ± 4.9	148.6 ± 13.9	37.2 ± 0.2	106.5 ± 0.5	>3.2	>8.9	4.2 (3.3–5.2)	11.7 (9.3–14.8)	-
**Miltefosine**	9.0 ± 0.5	22.1 ± 1.2	2.6 ± 0.3	6.5 ± 0.7	5.2 (4.8–5.5)	12.7 (11.8–13.6)	53.6 (49.7–57.6)	131.6 (122.0–141.2)	10.4
**RBH**	6.4 ± 0.4	-	7.2 ± 1.6	-	3.5 (2.9–4.2)	-	10.7 (9.2–12.3)	-	3.1

RBH: root bark hexane extract. Hemileiocarpin **(1)**, leiocarpin **(2**), connarin (**3**) rapanone (**4**), embelin (**5**) and suberonone (**6**).

^a^IC_50_: Concentration required to inhibit 50% of parasite growth after 72 h exposure.

^b^CC_50_: Cytotoxic concentration to reduce cell viability by 50% after 72 h exposure.

^c^SI: Selectivity Index.

^**d**^nt: Not tested.

^**e**^-: Not determined. Miltefosine was used as the positive control. Data reported as the average of 3 independent experiments performed in duplicate.

For the most active crude extracts, IC_50_ values of the promastigote tests were: RBH (6.4/7.2 μg/mL) (Table 3), while RWEtOAc (29.9/21.1 μg/mL) and RBEtOAc (74.3/68.0 μg/mL) against *L*. *amazonensis*/*L*. *infantum* (S2 Table). The reference drug miltefosine showed IC_50_ values of 9.0/2.6 μg/mL against *L*. *amazonensis*/*L*. *infantum* (Table 3 and S2 Table). The most promising extract against *L*. *amazonensis* amastigotes was also the root bark hexane extract (RBH) with IC_50_ 3.5 μg/mL (Table 3), while RWEtOAc (IC_50_ 26.6 μg/mL) and RBEtOAc (IC_50_ 58.6 μg/mL) only exhibited moderate activity (S2 Table).

The three isolated compounds (**1–3**) from the RBH extract exhibited antileishmanial activity (Table 3). Compound **3** showed strong activity against *L*. *amazonensis*/*L*. *infantum* promastigotes (IC_50_ 11.4 μM/13.3 μM) and *L*. *amazonensis* amastigotes (IC_50_ 2.9 μM). The latter was in fact more active against the intracellular stage than the miltefosine positive control (IC_50_ 12.7 μM). Compounds **1** and **2** exhibited moderate activity against amastigotes (IC_50_ 31.6 μM/20.4 μM) whereas compounds **4**, **5** and **6** were inactive. In addition, the most active compound, connarin (**3**), was also tested against *L*. *infantum* amastigotes, exhibiting an IC_50_ 7.7 μM (miltefosine control IC_50_ 3.7 μM). These data confirm the potent antileishmanial activity of compound **3**, with IC_50_ values < 10 μM against *Leishmania* amastigote forms.

Macrophages, *Leishmania* spp. host cells, were used to assess the cytotoxic effect of extracts and compounds **1**–**6** (Table 3 and S2 Table). The selectivity index (SI), the ratio of cytotoxicity/amastigotes (CC_50_/IC_50_), was calculated for compounds **2** (SI 5.7) and **3** (SI 6.3), both of which were more toxic to the intracellular parasites than the host cells.

Compound **3** was pinpointed by the VIP score/PCA loadings as the most important ion for group separation and, therefore, activity, demonstrating the highest activity among the samples tested. Both the RBH extract and **3** presented higher activity than the positive control against *L*. *amazonensis* promastigotes, confirming the results found in the metabolomics analysis.

Of the benzoquinones, embelin (**5**) with the shortest aliphatic chain (C11) was the most active against *L*. *amazonensis* promastigotes. Rapanone (**4**) was the most active against *L*. *infantum* promastigotes, despite its inactivity against *L*. *amazonensis* promastigotes, a result supported by the metabolomics data. Suberonone (**6**) was not considered active against either parasite (Table 3). A similar result was reported in a previous study in which no activity (IC_50_ >100 μg/mL) for a rapanone:suberonone mixture against *L*. *amazonensis* promastigotes was observed [9]_._ However, in the present study, rapanone demonstrated activity (7.2 μg/mL) against *L*. *infantum* promastigotes. The IC_50_ for *L*. *amazonensis* amastigotes was not determined for **4**–**6** due to the high degree of cytotoxicity in macrophages (Table 3).

A study of a neoflavonoid-containing dichloromethane extract from *Calophyllum brasiliense* (Calophyllaceae) leaves showed activity against *L*. *amazonensis* promastigotes (IC_50_ 40 μg/mL) and axenic amastigotes (IC_50_ 3.7 μg/mL). The authors isolated mammea A/BB, active against promastigotes (IC_50_ 3 μg/mL) and axenic amastigotes (IC_50_ 0.88 μg/mL) [26, 27]. Connarin (**3**) is a neoflavonoid with isoprenic groups and hydroxyl substituents similar to mammea A/BB. However, the former comprises a 4-aryl-coumarin structure and it has a 6-membered lactone ring fused to the aromatic ring, whereas in connarin (**3**) the lactone structure is substituted by a short link between the two aromatic rings, as it is a dalbergion derivative. In a study of mammea A/BB derivatives, the authors reported the structure-activity relationship against *L*. *amazonensis* promastigotes and amastigotes, relating that modifications made to 4-aryl-coumarin, such as the substitution of methoxy groups with hydroxyl groups, can significantly increase compound activity [26]. Moreover, some reported neoflavonoid structures had IC_50_ values similar to connarin (**3)**. This evidence suggests that groups such as isoprenic, methoxy or hydroxyl groups present in the two types of neoflavonoid are important for antileishmanial activity.

Cytotoxicity was reported for neoflavonoids with the same skeleton as **3** [28]. The authors reported that structural modifications of the “A” ring in compounds of this class, including the presence of the benzoquinone-like ring, increase neoflavonoid toxicity. The absence of this ring may account for the lower cytotoxicity observed for **3** in comparison with the benzoquinones (**4**–**6**).

Pterocarpans **1** and **2** were less toxic than the initial root bark hexane extract and compounds **3**–**6** (Table 3). Compound **1** (CC_50_ >100 μg/mL) was less toxic than the miltefosine control. Antioxidant and antimitotic activity were reported for **1** and **2**, respectively [21, 22]. Furthermore, Takahashi and co-authors [29] reported that the hydroxyl group at C-8 in 3,8-dihydroxy-9-methoxypterocarpan resulted in higher activity against *L*. *major* than compounds with no hydroxyl at this position. Another study of five pterocarpans against *L*. *donovani* related that the presence of a methoxyl substitution at C-9, in addition to a hydroxyl group at C-8, resulted in higher activity than a methoxyl group at each of the aforementioned positions [30]. Inhibitory activity was observed for hemileiocarpin (**1**) that only has the methoxyl substitution at C-9. The difference in compound toxicity could be linked to the C-9 position, with the methoxyl group (**1**) less toxic than the dioxolane ring (**2**) (Fig 3). However, this structural difference may also account for the activity of **2** against *L*. *infantum* promastigotes and *L*. *amazonensis* amastigotes (Table 3).

### Mechanism of action of compounds 2 and 3

**Fig 4:**
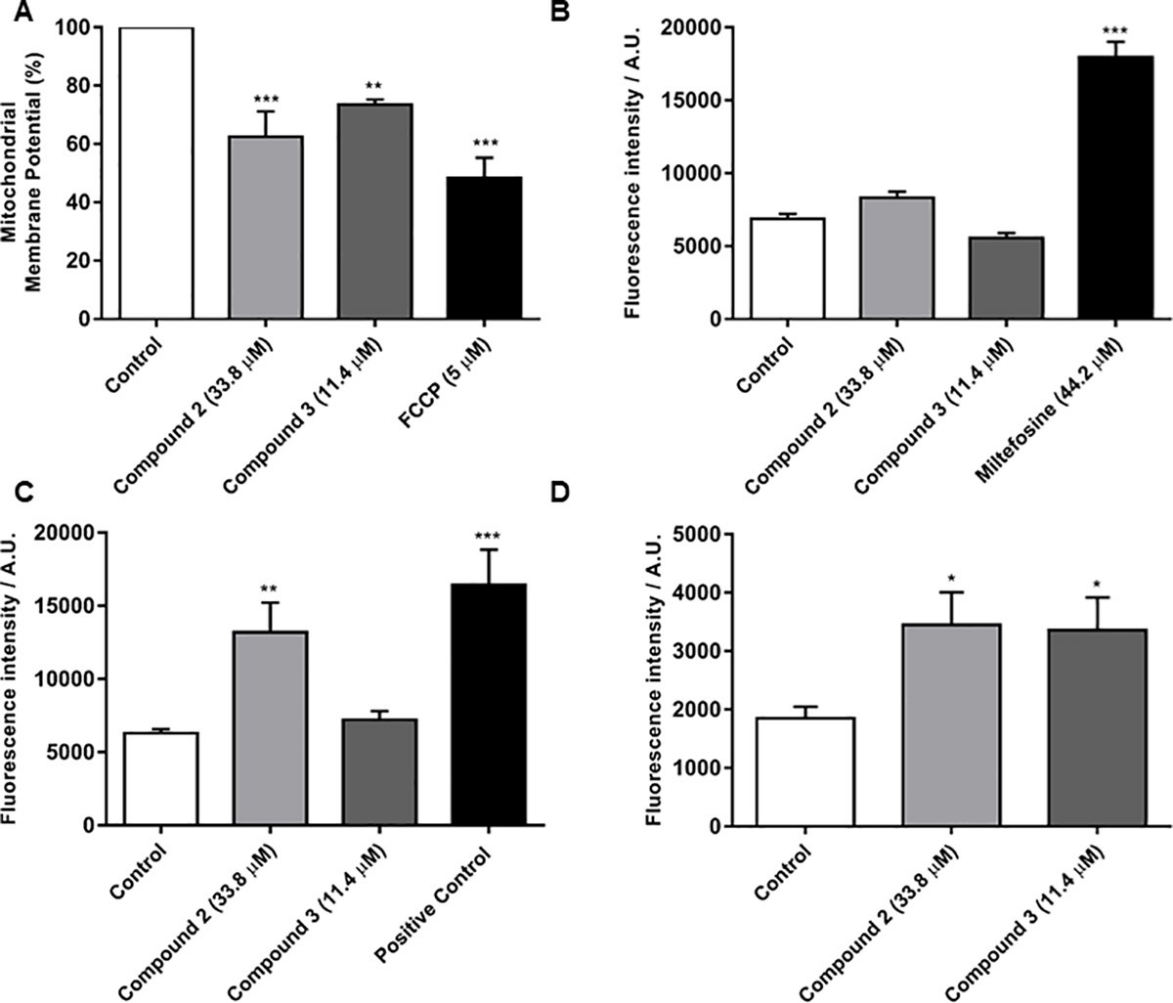
Mechanism of action of compounds 2 and 3 against *L*. *amazonensis* promastigotes after 24 h exposure. (A) Evaluation of mitochondrial membrane potential; positive control: FCCP. (B) Reactive oxygen species (ROS) detection; positive control: miltefosine. Fluorescence intensity expressed in arbitrary units (A.U.). (C) Plasma membrane integrity evaluation; positive control: parasites heated at 65°C. (D) Neutral lipid quantification; negative control: untreated parasites. P< 0.001 (***) and 0.01 (**), significant difference compared with the control (Dunnett’s test).

To better understand the antileishmanial mechanism of action of compounds **2** and **3**, some cellular targets/functions were investigated: (i) mitochondrial function, (ii) reactive oxygen species (ROS) production, (iii) plasma membrane integrity, and (iv) accumulation of lipid bodies (Fig 4A–4D).

The mitochondrial membrane potential (ΔΨm) was analyzed to determine the effect of **2** and **3** on mitochondrial function. Results showed a marked reduction in ΔΨm of 37.6% and 26.5% in *L*. *amazonensis* promastigotes treated with **2** (33.8 μM) and **3** (11.4 μM), respectively. The ΔΨm reduction after incubation with FCCP, a potent uncoupler of oxidative phosphorylation, was 51.6% (Fig 4A). Notable differences from host cells in terms of bioenergetic metabolism, antioxidant enzymes and a specific organization of mitochondrial DNA into a compartmentalized structure make this a selective target for the development of antileishmanial agents [31–33]. In the literature, different flavonoid compounds, such as epigallocatechin-3-gallate, strychnobiflavone and apigenin, induce *Leishmania* parasite death via depolarization of the mitochondrial membrane potential [34–36]. Our results revealed that compounds **2** and **3**, both flavonoid derivatives, also exert antileishmanial action by disrupting mitochondrial function, as demonstrated by the decreased ΔΨm of *L*. *amazonensis* promastigotes (Fig 3A).

One effect of mitochondrial stress triggered by membrane potential depolarization is the overproduction of reactive oxygen species (ROS). To evaluate the ROS levels, treated parasites were incubated with H_2_DCFDA, a fluorescent dye used to detect several reactive oxygen products, including superoxide, hydroxyl radical, and hydrogen peroxide. However, no alteration in the ROS level was detected in promastigotes treated with compounds **2** and **3** after 24 h. Miltefosine treatment induced a significant increase in ROS production of 162.3% (Fig 4B). Quercetin and resveratrol are flavonoids which possess effective antioxidant properties and are capable of neutralizing pathogens by scavenging ROS/acting in pathways that activate the parasite detoxification system, culminating in ROS degradation [37]. In the present study, **2** and **3** did not interfere with the ROS levels (Fig 4B), suggesting that the antioxidant metabolism is able to regulate these levels in the parasites tested.

Since the promastigote mitochondrial membrane was strongly affected by **2** and **3**, plasma membrane integrity was therefore analyzed using a propidium iodide (PI) fluorescent probe. Fig 4(C) indicates a significant increase in the percentage of PI-positive parasites after treatment with **2**, indicative of plasma membrane rupture whereas **3** did not interfere with plasma membrane permeation. Plasma membrane damage causes parasite death, as it interferes with ion/nutrient transportation and pH homeostasis [38].

Flavonoids have been reported for their anticancer/antioxidant properties by interacting with plasma membrane surface lipids. Consequently, membrane structural/functional integrity is compromised, culminating in cell death [39, 40]. The effect of **2** and **3** on the accumulation of neutral lipid bodies was assessed to evaluate changes in the parasite lipid profile (Fig 4D). *L*. *amazonensis* promastigotes treated with **2** and **3** showed an increase in neutral lipid levels of 86.9% and 82.0% in relation to the untreated control, respectively. Flavonoid-lipid membrane component interactions suggested that the increase in neutral lipid bodies post-treatment may be associated with alterations in lipid composition of the parasite [40]. Moreover, studies have closely-associated the increase in neutral lipid formation in trypanosomatids as a hallmark of cellular stress conditions and mitochondrial dysfunction [41, 42].

### Transmission Electron Microscopy (TEM)

**Fig 5:**
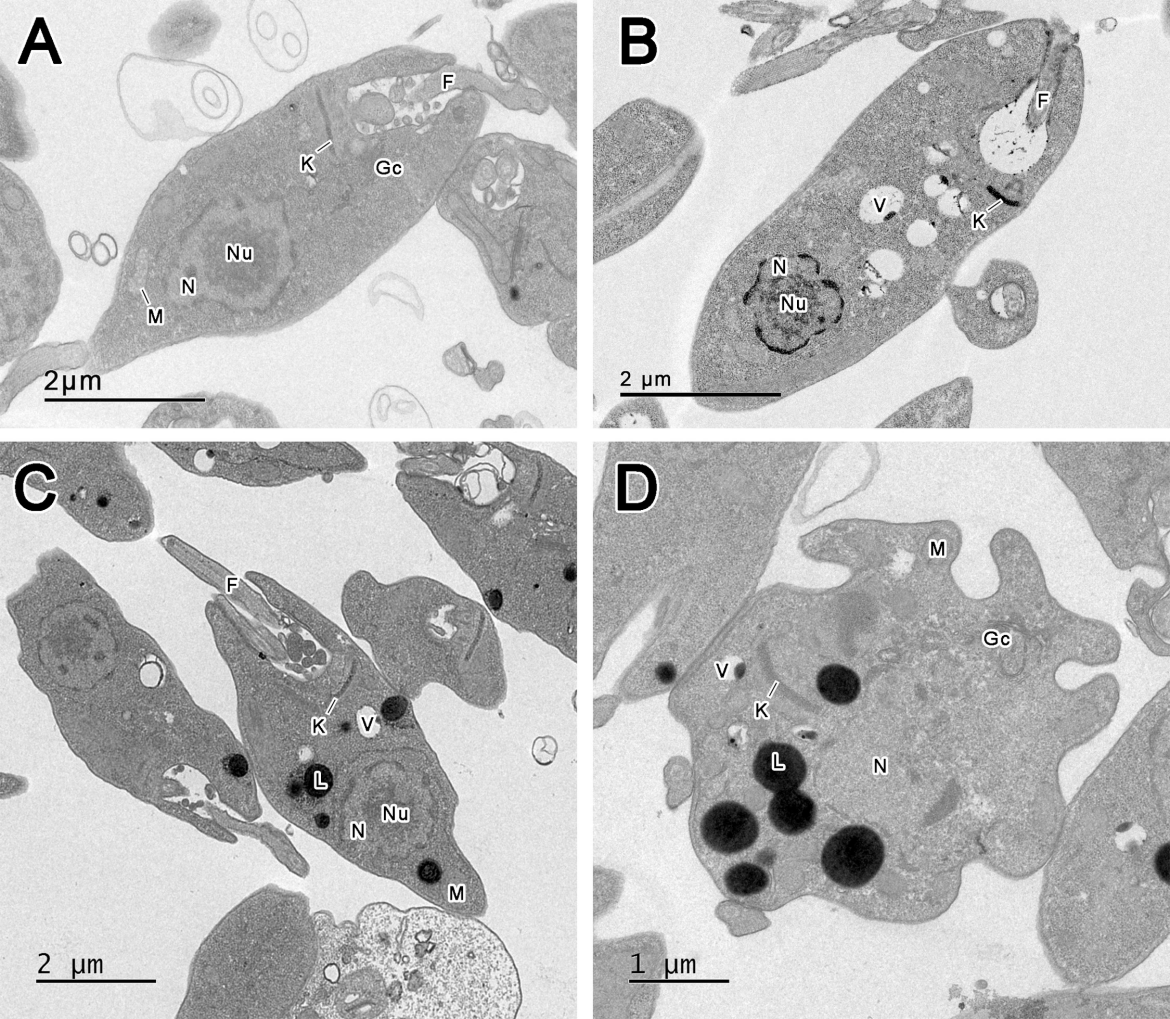
Micrographs of *Leishmania amazonensis* promastigotes. (A*)* Control parasites incubated with Warren’s medium; (B) Control parasites incubated with 0.01% DMSO; (C-D) *L*. *amazonensis* treated with connarin (**3**) showing (L) lipid bodies. (F) Flagellum; (Gc) Golgi complex; (K) Kinetoplast; (M) Mitochondria; (N) Nucleus; (Nu) Nucleolus; (V) Vacuole.

Connarin (**3**), an unreported compound, induced a marked increase in neutral lipid bodies in *L*. *amazonensis* promastigotes. TEM confirmed ultrastructural modification and supported the neutral lipid quantification (Nile red stain) experiment. Fig 5 shows normal organelle structure (5A), with some vacuoles visible in promastigotes in the presence of 0.01% DMSO (5B). Promastigotes exposed to connarin (IC_50_ 11.4 μM, for 24 h) showed lipid bodies and the presence of vacuoles (5C/5D). Since the treatment was for 24 h at the IC_50_ concentration, it is possible that the connarin mechanism of action commences with intense lipid body (LB) formation and culminates in other cell disorders. Many studies point to the formation of LB as the start of apoptosis [43, 44].

## Conclusions

In this contribution, 9 *C*. *suberosus* extracts tested against *Leishmania* species were analyzed using non-target metabolomics, revealing a potentially new compound pinpointed as the most important for the differentiation of active samples. This unreported compound was isolated and identified as connarin (**3**), which presented activity against *L*. *amazonensis* and *L*. *infantum*. Of the 6 compounds tested, connarin (**3**) was the most active against *L*. *amazonensis* and *L*. *infantum* promastigotes and amastigotes. Mechanistic studies of this compound against *L*. *amazonensis* promastigotes demonstrated impaired mitochondrial function and parasite lipid profile alterations. Leiocarpin (**2**), a pterocarpan, also demonstrated antileishmanial activity with a similar mechanism of action, including plasma membrane rupture. Transmission electron microscopy of compound **3** supported the Nile Red staining data, exhibiting a high number of lipid bodies, suggesting the onset of apoptosis. There are few studies of pterocarpans (**1** and **2**), however, this is the first study of connarin (**3**) (neoflavonoid—dalbergion derivative) with activity against *Leishmania* species. This work showed the importance and efficacy of non-target metabolomics and proved that it is a more streamlined isolation strategy. Furthermore, it described the activity of extracts and compounds from *C*. *suberosus* and the mechanism of action of connarin (**3**) and leiocarpin (**2**), adding to knowledge to enable the development of potential prototypes with antileishmanial activity, to fight against this neglected tropical disease.

## Materials and methods

### General experimental procedures

IR spectra were measured on a Varian 640-IRFT-IR spectrophotometer. NMR spectra were run on a 300 MHz Varian Magnet Oxford YH300 Console Mercury Plus 300 (^13^C) and Bruker Avance III 600 MHz (^1^H, COSY, HMBC, HSQC). The chemical shift (δ) values are in ppm (parts per million) referenced with TMS (tetramethylsilane). High-resolution mass spectra were obtained using an AB SCIEX TripleTOF 5600+ mass spectrometer in the positive mode by direct infusion. Diol SPE fractionation of the extract was performed in DIO Spe-ed SPE cartridges (Applied Separations, Allentown, PA, USA) using hexane/CH_2_Cl_2_ (9:1—A), CH_2_Cl_2_/EtOAc (20:1—B), EtOAc (C), EtOAc/MeOH (5:1—D) and MeOH (E). Silica gel 60 (0.04 mm—0.063 mm, 230–400 mesh) using hexane with 3% EtOAc increasing polarity until 100% MeOH and Sephadex LH-20(CH_2_Cl_2_/4MeOH 1:1) were used for column chromatography. Silica gel plates were used for TLC. For PTLC, preparative layer with UV_254_, 500 microns were used with hexane/CH_2_Cl_2_ (4:6).

### Plant material

*Connarus suberosus* Planch. (Connaraceae) was collected and identified in 2010, at Lagoa Formosa, Planaltina, Distrito Federal, 15° 27’ 34.2” S; 47° 92’ 3.3” W; 1071 m together with the botanist Prof. José Elias de Paula. A specimen voucher was deposited at the Herbarium of Universidade de Brasília, under the access number (UB) 3820. Plant access was authorized by the Genetic Heritage Management Council (CGen)/ Brazilian Institute of the Environment and Renewable Natural Resources (IBAMA) n. 06/2012-Process 02000.002272/2006-73.

### Extraction and isolation

*Connarus suberosus* Planch. extracts were selected from the “Brazilian Cerrado Biome Plant Extract Bank” (Laboratório de Farmacognosia, Universidade de Brasília) for this study. These extracts were previously prepared from separated plant organs that were dried, powdered and extracted by maceration with: hexane, ethyl acetate or ethanol and stored at—20°C. The activity of 9 different extracts was investigated against *Leishmania* spp.: root wood hexane (RWH); root bark hexane (RBH); stem wood hexane (SWH); stem bark ethyl acetate (SBEtOAc); stem wood ethyl acetate (SWEtOAc); leaf ethyl acetate (LEtOAc); root wood ethyl acetate (RWEtOAc); root bark ethyl acetate (RBEtOAc); root wood ethanol (RWEtOH). The most active extract, root bark hexane (1.8 g), was pre-fractionated in solid phase extraction cartridges (2 g, 6 mL Diol cartridge). The extract was solubilized in 19.2 mL of 1:1 CH_2_Cl_2_/MeOH and applied to 12 different cartridges. After overnight drying, the cartridges were eluted with 6 mL/cartridge of 5 different solvent systems: A—9:1 hexane/CH_2_Cl_2_ (60.6%); B—20:1 CH_2_Cl_2_/EtOAc (28.3%); C—EtOAc (2.8%); D—5:1 EtOAc/MeOH (0.4%), and E—MeOH (1.1%). Based on the yield and biological results, Fraction A (1.09 g) was submitted to a silica gel column with a gradient of hexane/EtOAc/MeOH from 97:3:0 to 0:0:100, resulting in 29 fractions (A1-A29). Fractions A10 (1.6%) and A11 (5.0%) were submitted to PTLC eluted with 1:1 hexane/CH_2_Cl_2_, yielding compounds **1** and **2,** respectively. Fraction A22 (42.1%) was fractionated in a Sephadex LH-20 (Sigma®) column eluted with 1:1 CH_2_Cl_2_/MeOH, yielding 5 fractions: A22.1 (2.4%), A22.2 (2.9%), A22.3 (20.1%), A22.4 (58.0%) and A22.5 (0.3%). Fraction A22.4 was characterized as compound **3**.

### Metabolomics

Extracts (25 mg) were diluted in 800 μL CH_2_Cl_2_:MeOH (1:1) and added to SPE LC-18 cartridges (Supelclean LC-18, Supelco). After sample incorporation and drying, 3 mL of MeOH were used for extraction. Samples were subsequently dried, resuspended in MeOH (1 mg/mL), filtered (0.22 μm) and submitted for UHPLC-MS/MS analysis (Bruker Daltonics, Elute pump UHPLC, Elute autosampler UHPLC, Elute DAD) with an ESI-qTOF mass spectrometer (Compact otofControl Bruker Daltonics). For chromatographic separation, a C18 ODS column (100 x 2.1 mm, 1.8 μm particle size) was used; HPLC grade acetonitrile (JTBaker®) and ultrapure water acidified with 0.1% formic acid as mobile phases. The gradient elution method started with 5% acetonitrile and increased to 98% in 13 min; with a final 3 min wash to stabilize the column. The column temperature was 40°C with a 0.5 mL/min flow rate. Source parameters: 4500V non-capillary, nebulizer 4 bar and dry gas 9 L/min. The source temperature was 200°C. Metabolomic analysis of crude extracts was conducted using both the UHPLC-MS data and the activity observed against *L*. *amazonensis* promastigotes at 100 μg/mL. A crude extract causing >80% mortality was considered active. The data obtained from the analysis (.d format) were converted to mzXML format using the MsConvert software. Data were then preprocessed with the MZmine software using the following modules: mass detection (RT 2.0–11.5 min, centroid); chromatogram builder (MS level 1; minimum height 5 × 10^3^; minimum time span 0.05 min; *m/z* tolerance 20 ppm); deconvolution of the spectra (Algorithm Noise amplitude); isotopic peaks grouper (*m/z* tolerance 20 ppm; RT tolerance 2 min); duplicate peak filtering; smoothing; data alignment (Join aligner; *m/z* tolerance 20 ppm; RT tolerance 2 min); gap-filling (intensity tolerance 5%; *m/z* tolerance 20 ppm, RT tolerance 2 min); and peak filtering range (0.0–2.0 min). The data were exported and uploaded to the MetaboAnalyst® platform as a table containing the code name, active/inactive label and ion intensity of each extract. The data integrity check was default, data filtering was performed by mean intensity value and normalization performed by Pareto data scaling.

### Absolute configuration of compound 3 determined by VCD spectroscopy

The infrared and vibrational CD spectra of **3** were acquired on a Bruker Vertex 70 equipped with a PMA 50 unit for modulated polarization measurements [17, 18]. A solution of (**3**) was prepared at 65 mg/mL (~0.16 M) and spectra recorded using a 100 μm cell with BaF_2_ windows. The VCD spectrum was recorded at a spectral resolution of 4 cm^-1^ by accumulating 42000 scans (10 h). Baseline correction of the VCD spectrum was achieved by subtraction of the solvent spectrum (CDCl_3_) recorded under identical conditions. Density functional theory (DFT)-based spectra calculations were performed using the Gaussian09 Rev E.01 [45] at the b3lyp/6-311+g(2d,p)/IEFPCM(CHCl_3_) level of theory. A systematic conformational search was performed for (**3**) around C-7/C-8 (starting values of ƙ = -140°, 10°, 120°), C-7’/C-1”‘ (δ_1_ = 0°, 180°), C-1”‘/C-2”‘ (δ_2_ = -90°, 90°, 180°), C-5/C-1” (δ_3_ = 0°, 180°) and C-1”/C-2” (δ_4_ = -90°, 90°, 180°). For the two hydroxyl groups, we considered intramolecular hydrogen bonding in two orientations: C2’–O (β = 12°) and C2–O (α = -175), or C2’–O (β = 175°) and C2–O (α = -20°). The total number of conformers was 216.

### Biological assays

#### Parasites

*Leishmania amazonensis* promastigotes (IFLA/BR/1967/PH8) for the 24 h exposure test were cultivated in RPMI 1640, supplemented with 20% *v/v* heat inactivated fetal bovine serum (FBS) and incubated at 26°C until near the end of the log growth phase. *Leishmania amazonensis* (IFLA/BR/1967/PH8) and *L*. *infantum* (MCAN/BR/2008/1112) promastigotes used in the 72 h exposure test were cultivated in Warren’s medium (containing brain heart infusion- BHI plus hemin and folic acid), supplemented with 10% FBS, 0.1% of the antibiotics: 100 UI/mL penicillin G and 0.1 mg/mL streptomycin. *Leishmania amazonensis* (IFLA/BR/1967/PH8) transfected with the red fluorescent protein (RFP) gene was cultured in M199 medium (containing 0.1 mmol/L adenine, 40 mmol/L hepes, 0.0005% hemin and 0.0002% biotin), supplemented with 10% FBS, and 0.1% of the antibiotic. Parasites were maintained at 25°C in a BOD incubator.

#### Antiproliferation assay: *L*. *amazonensis* promastigotes (24 h)

Extracts and fractions were tested against *L*. *amazonensis* (IFLA/BR/1967/PH8) promastigote cells in a 96-well plate at a concentration of 10^6^ cells/well. Each sample was solubilized in DMSO (<1%) since it was not soluble directly in the medium. Extracts were tested at 100 μg/mL and 50 μg/mL. Diol SPE fractions were subsequently serially diluted from 100 μg/mL to 3.125 μg/mL. Silica gel column collected fractions were tested at 100 μg/mL. Active samples were those which caused >80% mortality. Amphotericin B and DMSO were used as positive and negative controls, respectively. After 24 h of incubation at 26°C, 20 μL resazurin solution (1.5 mM) was added to each well to assess viability. Plates were read at 570 nm and 595 nm in a microplate reader (BiochromAsys UVM 340) after cell lysis with sodium dodecyl sulfate (SDS). Viability was calculated based on the equations described by Kulshrestha [46]. The most active fractions were fractionated and tested until isolation of the active compounds.

#### Antiproliferation assay: *L*. *amazonensis* and *L*. *infantum* promastigotes (72 h)

Extracts and isolated compounds were tested against *L*. *amazonensis* promastigotes (IFLA/BR/1967/PH8 strain - 2x10^5^ cells/well) and *L*. *infantum* promastigotes (MCAN/BR/2008/1112 strain - 3x10^5^ cells/well). The samples were solubilized in DMSO (<1%), serially diluted (100 μg/mL to 1.56 μg/mL) and tested in a 96-well plate to obtain a dose-response curve. Miltefosine and DMSO were used as positive and negative controls, respectively. After 72 h, 10 μL of 3-(4,5-dimethylthiazol-2-yl)-2,5-diphenyl tetrazolium bromide (5 mg/mL—MTT, Sigma-Aldrich) solution was added to each well and the plate returned to the incubator for an additional 4 h. To halt the reaction, 100 μL acidified isopropanol was added to each well. Plates were read at 570 nm in a microplate reader (Multiskan MS, LabSystems Oy, Helsinki, Finland) [47].

#### *L*. *amazonensis* amastigotes assay

Peritoneal macrophages obtained from BALB/c mice were transferred to a 24-well plate (6x10^5^ cells/well) in RPMI 1640 medium supplemented with 10% inactivated FBS and 20 mM L-glutamine, pH 7.2. The plate was incubated at 37°C in 5% CO_2_ for 24 h. After this period, macrophages were infected with *L*. *amazonensis* (IFLA/BR/1967/PH8) transfected with RFP in the stationary growth phase using a ratio 1:10 (macrophages/parasites) at 33°C for 4 h. The non-phagocytosed parasites were subsequently removed by extensive PBS wash, and samples added at non-toxic concentrations, in accordance with the cytotoxicity tests (100 μg/mL to 1.56 μg/mL) to determine the IC_50_ for the intracellular parasite. After 72 h, the supernatant was removed from the wells and 200 μL of deionized water added to each well to lyse the cell membrane. After lysis, the resulting supernatants were transferred to a black 96-well plate. The plate was read in a fluorescence spectrophotometer (FLx800, BioTek Instruments, Inc., Winooski, VT, USA) with 540/600 nm of excitation/emission. Wells containing macrophages infected with *L*. *amazonensis* (RFP) (25 μg/mL to 1.56 μg/mL) only were used as negative controls. The positive controls were similar, but with the addition of miltefosine.

#### *L*. *infantum* amastigotes assay

Peritoneal macrophages (6 x 10^5^ cells) were plated on round glass coverslips within 24-well plates in RPMI 1640 medium supplemented with FBS and 20 mM L-glutamine, pH 7.2, and incubated for 24 h at 37°C in 5% CO_2_. Stationary-phase promastigotes (6 x 10^6^ cells, at a ratio of 10 parasites per macrophage) were then added to the wells, and cultures incubated for 24 h at 37°C in 5% CO_2_. Non-internalized parasites were removed by extensive PBS solution washing and infected macrophages subsequently treated with nontoxic concentrations of: compound **3** (6.25 μg/mL to 0.18 μg/mL) or miltefosine (10.0 μg/mL to 0.6 μg/mL, positive control) for 72 h at 37°C in 5% CO_2_. After compound exposure, cells were fixed with absolute ethanol and stained with Giemsa. A total of 200 macrophages were counted under a light microscope. The infection index was determined by the ratio of percentage of infected macrophages multiplied by the mean number of amastigotes per macrophage out of 200 macrophages.

#### Cytotoxicity assay

Female BALB/c mice pre-inoculated with 3% thioglycollate medium were used to obtain macrophages from the peritoneal cavity. Peritoneal exudate was collected 72 h post-inoculation using cold Hank´s Balanced Salt Solution and the assay performed as previously reported [36]. Briefly, peritoneal macrophages in RPMI 1640 medium were transferred to a 96-well plate (2x10^5^ cells/well) and incubated at 37°C in 5% CO_2_ for 24 h to allow adhesion. Samples were serially diluted (100 μg/mL to 0.78 μg/mL), added to the plate with the cells and incubated under the same conditions for 72 h. MTT (10 μL/well) was subsequently added and the plates re-incubated for a further 2 h. To halt the reaction, 100 μL of acidified isopropanol was added to each well. The plate was then read at 570 nm in a microplate reader (Multiskan MS, LabSystems Oy, Helsinki, Finland).

### Mechanism of action assays

#### Mitochondrial membrane potential

The mitochondrial membrane potential (ΔΨm) of *L*. *amazonensis* promastigotes (10^7^ cells/mL) was analyzed after individual treatment with compound **2** (33.8 μM) or **3** (11.4 μM), both concentrations corresponded to the IC_50_ obtained for promastigotes. After 24 h of incubation at 25°C, the parasite concentration was adjusted to 5x10^6^ cells/mL and stained with 500 nM MitoTracker (Invitrogen, Eugene, OR, USA) in the dark for 40 min at 37°C. After washing twice with PBS, samples were transferred in triplicate to a black 96-well plate, and fluorescence measured in a spectrofluorometer (FLx800, BioTek Instruments, Inc., Winooski, VT, USA) at 540/600 nm excitation/emission. Promastigotes incubated with 5.0 μM carbonyl cyanide 4-(trifluoro-methoxyl)phenylhydrazone (FCCP, Sigma-Aldrich, St. Louis, MO, USA) for 10 min were used to obtain maximal mitochondrial depolarization (positive control) [48].

#### Reactive Oxygen Species (ROS) detection

*Leishmania amazonensis* promastigotes (10^7^ cells/mL) were untreated or treated with compound **2** (33.8 μM) or **3** (11.4 μM) for 24 h at 25°C. Parasite concentrations were subsequently adjusted (1x10^7^ promastigotes/well) and plates incubated with 20 μM H_2_DCFDA (2’,7’-dichlorodihydrofluorescein diacetate—Molecular Probes, Eugene, OR, USA) for 30 min in the dark at RT (room temperature). Fluorescence intensity was detected using a fluorimetric microplate reader (FLx800, BioTek Instruments, Inc., Winooski, VT, USA), at 485/528 nm excitation/emission. Promastigotes incubated with miltefosine (44.2 μM) were used as a positive control [49].

#### Cell membrane integrity

*Leishmania amazonensis* promastigotes (10^7^ cells/mL) were treated with compound **2** (33.8 μM) or **3** (11.4 μM) at 25°C. After 24 h of exposure to compounds, parasites were washed with PBS and concentrations adjusted to 1x10^7^ promastigotes/well. Plates were incubated with propidium iodide (PI) (1.0 μg/mL—Sigma-Aldrich, USA, St. Louis, MO, USA) for 15 min in the dark at RT. Fluorescence was measured using a spectrofluorometer (FLx800, BioTek Instruments, Inc., Winooski, VT, USA) with 540/600 nm excitation/emission. Parasites heated at 65 ^o^C were used as the positive control [50].

#### Neutral lipid quantification

*Leishmania amazonensis* promastigotes (10^7^ cells/mL) were incubated with compound **2** (33.8 μM) or **3** (11.4 μM). After 24 h of exposure, the parasites (1x10^7^ cells/well) were stained with 10 μg/mL Nile Red (Sigma-Aldrich, St. Louis, MO, USA) in the dark for 20 min at 25°C. Fluorescence intensity was analysed in a spectrofluorometer (BioTek Instruments, Inc., Winooski, VT, USA) using 485/528 nm excitation/emission [48].

#### Transmission Electron Microscopy (TEM)

*Leishmania amazonensis* promastigotes (10^7^ cells/mL) were treated with compound **3** (11.4 μM) or incubated with controls (medium alone or medium plus 0.01% DMSO) for 24 h at 26°C. The parasites were subsequently washed in PBS, fixed in 2.5% glutaraldehyde (in 0.1 M sodium cacodylate buffer) at pH 7.2 for 45 min at room temperature. After fixation, the specimens were washed three times successively with 0.1 M sodium cacodylate buffer, and postfixed in 1% osmium tetroxide, 0.8% potassium ferricyanide, and 5 mM of the CaCl_2_ in 0.1 M of the sodium cacodylate buffer (0.1 M, pH 7.2). Dehydration was performed in acetone, followed by the embedding resin (Spur, Sigma-Aldrich^®^). Ultrathin sections were contrasted with uranyl acetate and lead citrate and observed under a transmission electron microscope (JEOL 1011).

#### Ethics and mice

Female 6–8 week-old BALB/c mice were purchased from the Reproductive Biology Center of the Universidade Federal de Juiz de Fora (UFJF) and maintained under specific pathogen-free conditions. The study was approved by the Committee of Ethical Handling of Research Animals (CEUA) of UFJF under Protocol Numbers: 007/2018 and 008/2018.

#### Statistical analyses

The IC_50_ and CC_50_ values were calculated using the GraFit version 5.0 (Erithacus Software Ltd., Horley, UK) and Probit programs. Data obtained from the mechanism of action assays were analysed by One-way ANOVA followed by Dunnett’s post-test to compare the group using Prism 5 software (GraphPad Software, San Diego, CA, USA). Results were expressed as the mean ± standard error of the mean of 3 independent experiments, which presented similar results. P values < 0.05 (*), < 0.01 (**) and < 0.001 (***) were considered statistically significant.

## Supporting information

S1 Fig Activity of silica column fractions of *C*. *suberosus* Root Bark Hexane (RBH) extract. (A) Inhibitory effect of fractions A17-A27 (100 μg/mL) against *L*. *amazonensis* promastigotes. *Statistically significant (p < 0.05) when compared to the DMSO negative control using the Dunnett's test. (B) Dose-response curve of connarin (**3**) against *L*. *amazonensis* promastigotes after 24 h exposure.
(PDF)

S2 Fig Vibrational circular dichroism and infrared experimental (exptl) spectra of connarin (3) in comparison with calculated (calc) spectra for (S) configuration. (PDF)

S3 Fig ^1^H NMR spectrum (600 MHz, CDCl_3_) of hemileiocarpin (1). (PDF)

S4 Fig ^13^C NMR spectrum (75 MHz, CDCl_3_) of hemileiocarpin (1). (PDF)

S5 Fig Edited HSQC spectrum (CDCl_3_) of hemileiocarpin (1). (PDF)

S6 Fig HMBC spectrum (CDCl_3_) of hemileiocarpin (1). (PDF)

S7 Fig COSY spectrum (CDCl_3_) of hemileiocarpin (1). (PDF)

S8 Fig HRESIMS spectrum of hemileiocarpin (1). (PDF)

S9 Fig 1H NMR spectrum (600 MHz CDCl_3_) of leiocarpin (2). (PDF)

S10 Fig 13C NMR spectrum (75 MHz, CDCl_3_) of leiocarpin (2). (PDF)

S11 Fig Edited HSQC spectrum (CDCl_3_) of leiocarpin (2). (PDF)

S12 Fig HMBC spectrum (CDCl_3_) of leiocarpin (2). (PDF)

S13 Fig COSY spectrum (CDCl_3_) of leiocarpin (2). (PDF)

S14 Fig HRESIMS spectrum of leiocarpin (2). (PDF)

S15 Fig 1H NMR spectrum (600 MHz CDCl_3_) of connarin (3). (PDF)

S16 Fig 13C NMR spectrum (150 MHz, CDCl_3_) of connarin (3). (PDF)

S17 Fig Edited HSQC spectrum (CDCl_3_) of connarin (3). (PDF)

S18 Fig HMBC selected correlations of connarin (3). (PDF)

S19 Fig HMBC spectrum (CDCl_3_) of connarin (3). (PDF)

S20 Fig COSY spectrum (CDCl_3_) of connarin (3). (PDF)

S21 Fig DEPT spectrum (150 MHz, CDCl_3_) of connarin (3). (PDF)

S22 Fig IR spectrum of connarin (3). (PDF)

S23 Fig HRESIMS spectrum of connarin (3). (PDF)

S24 Fig Systematic conformational search performed for connarin (3). (PDF)

S1 Table Viability of *L*. *amazonensis* promastigotes after 24 h exposure to *C*. *suberosus* crude extracts at 100 μg/mL and 50 μg/mL. (PDF)

S2 Table *In vitro* antileishmanial activity, cytotoxicity in murine peritoneal macrophages, and selectivity index of *C*. *suberosus* extracts. (PDF)

S3 Table ^1^H (600 MHz) and ^13^C (75 MHz) NMR data assignments for hemileiocarpin (1) and leiocarpin (2). Chemical shifts (ppm) and coupling constants (J, Hz, in parenthesis).
(PDF)

S4 Table Starting angles of selected conformers for connarin (3). (PDF)

S1 File Cartesian coordinates of selected conformers for connarin (3). (PDF)

S1 Data (PDF)
