# Supplementary material for: Antileishmanial compounds from Connarus suberosus: Metabolomics, isolation and mechanism of action
Source: PLoS One. 2020 Nov 6;15(11):e0241855. doi: 10.1371/journal.pone.0241855 (PMC7647111; doi:10.1371/journal.pone.0241855)
Supplement: S1 Fig — (A) Inhibitory effect of fractions A17-A27 (100 μg/mL) against L. amazonensis promastigotes. *Statistically significant (p < 0.05) when compared to the DMSO negative control using the Dunnett's test. (B) Dose-response curve of connarin (3) against L. amazonensis promastigotes after 24 h exposure. (PDF) [file pone.0241855.s001.pdf]

(A)

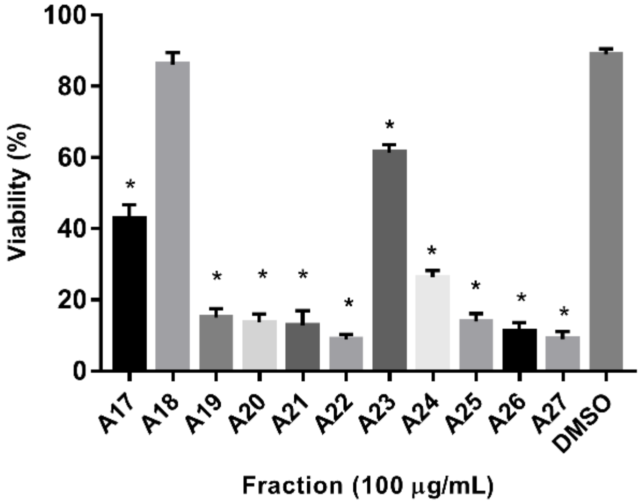

(B)

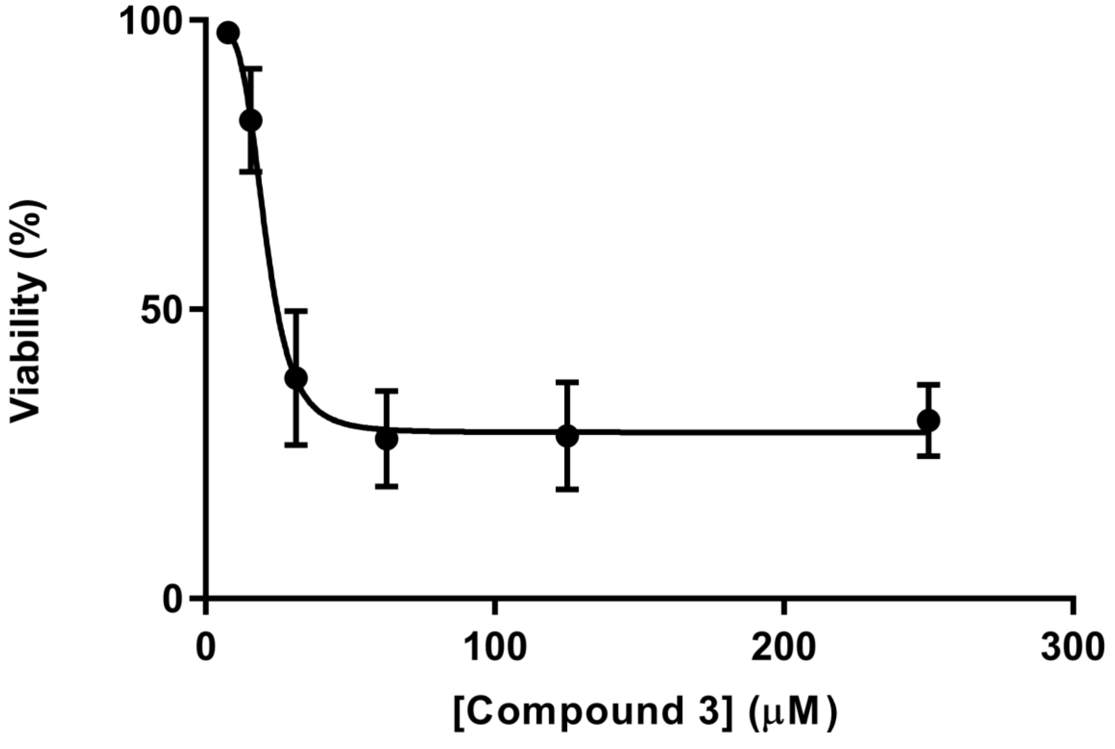

S1 Fig. Activity of silica column fractions of *C. suberosus* root bark hexane (RBH) extract. (A) Inhibitory effect of fractions A17-A27 (100 μg/mL) against *L. amazonensis* promastigotes. *Statistically significant ($p < 0.05$) when compared to the DMSO negative control using the Dunnett's test. **(B)** Dose-response curve of connarin (**3**) against *L. amazonensis* promastigotes after 24 h exposure
