# Supplementary material for: Antileishmanial compounds from Connarus suberosus: Metabolomics, isolation and mechanism of action
Source: PLoS One. 2020 Nov 6;15(11):e0241855. doi: 10.1371/journal.pone.0241855 (PMC7647111; doi:10.1371/journal.pone.0241855)
Supplement: S2 Fig — (PDF) [file pone.0241855.s002.pdf]

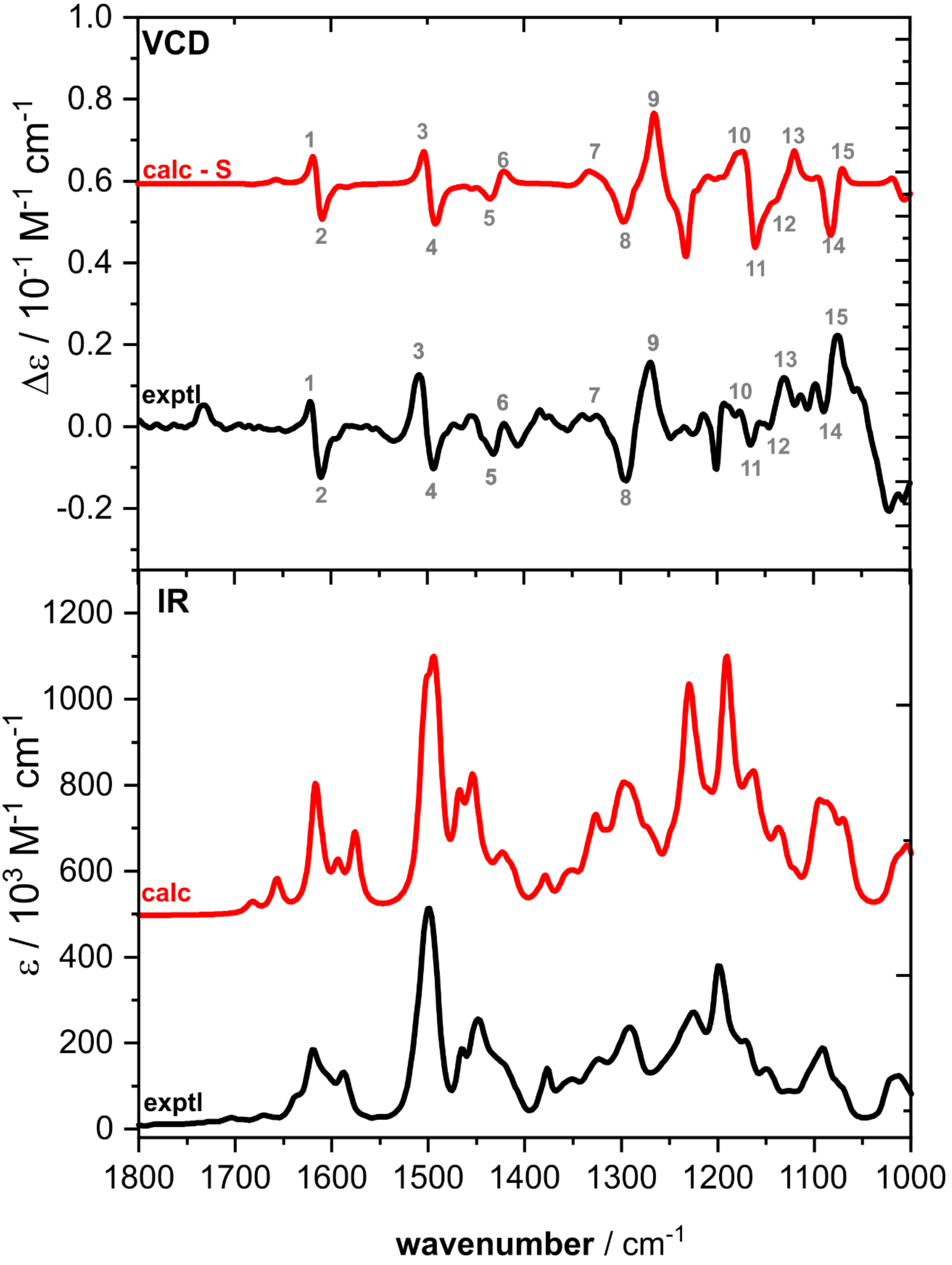

S2 Fig. Vibrational circular dichroism and infrared experimental (exptl) spectra of connarin (3) in comparison with calculated (calc) spectra for (S) configuration
