# Supplementary figures and images for: Antileishmanial compounds from Connarus suberosus: Metabolomics, isolation and mechanism of action
Source: PLoS One. 2020 Nov 6;15(11):e0241855. doi: 10.1371/journal.pone.0241855 (PMC7647111; doi:10.1371/journal.pone.0241855)

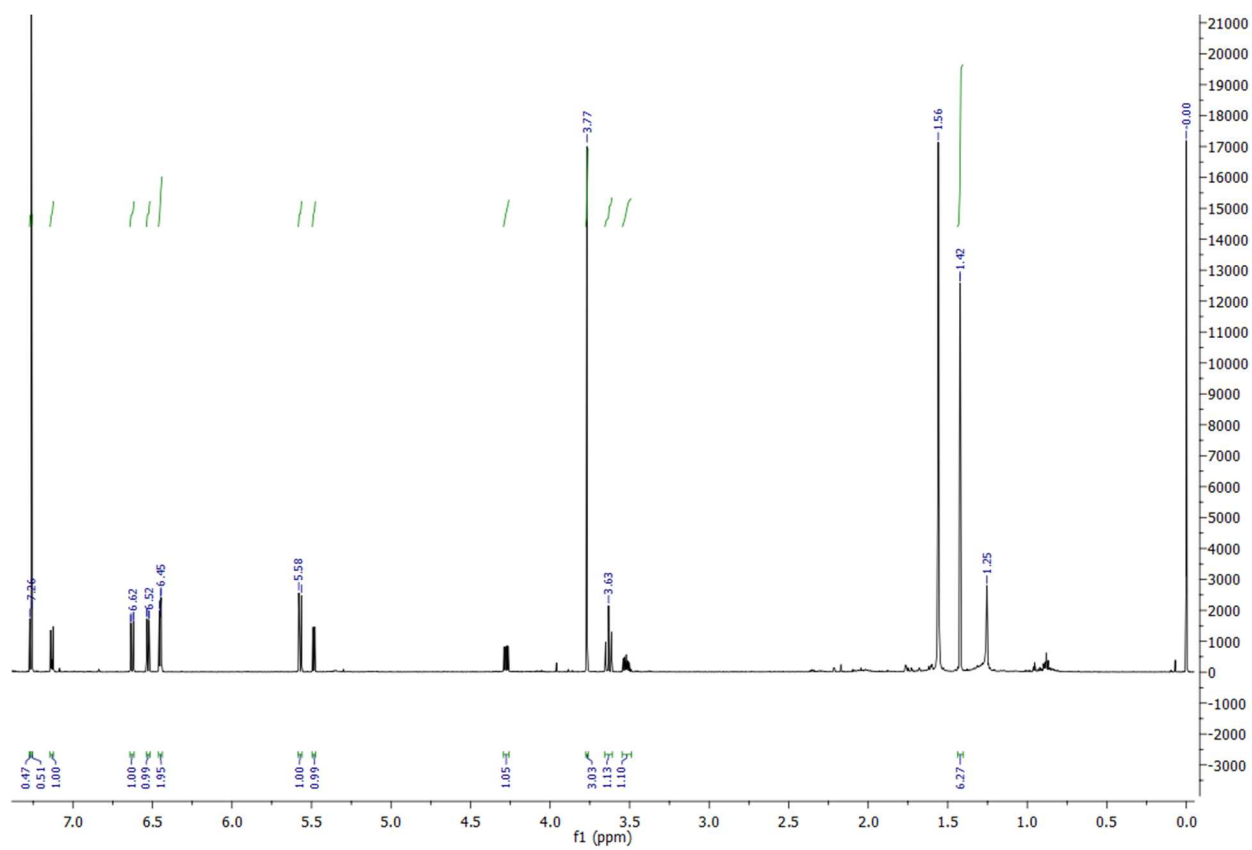

**S3 Fig.  $^1\text{H}$  NMR spectrum (600 MHz,  $\text{CDCl}_3$ ) of hemileiocarpin (1)**

Supplement: S3 Fig — (PDF) [file pone.0241855.s003.pdf]

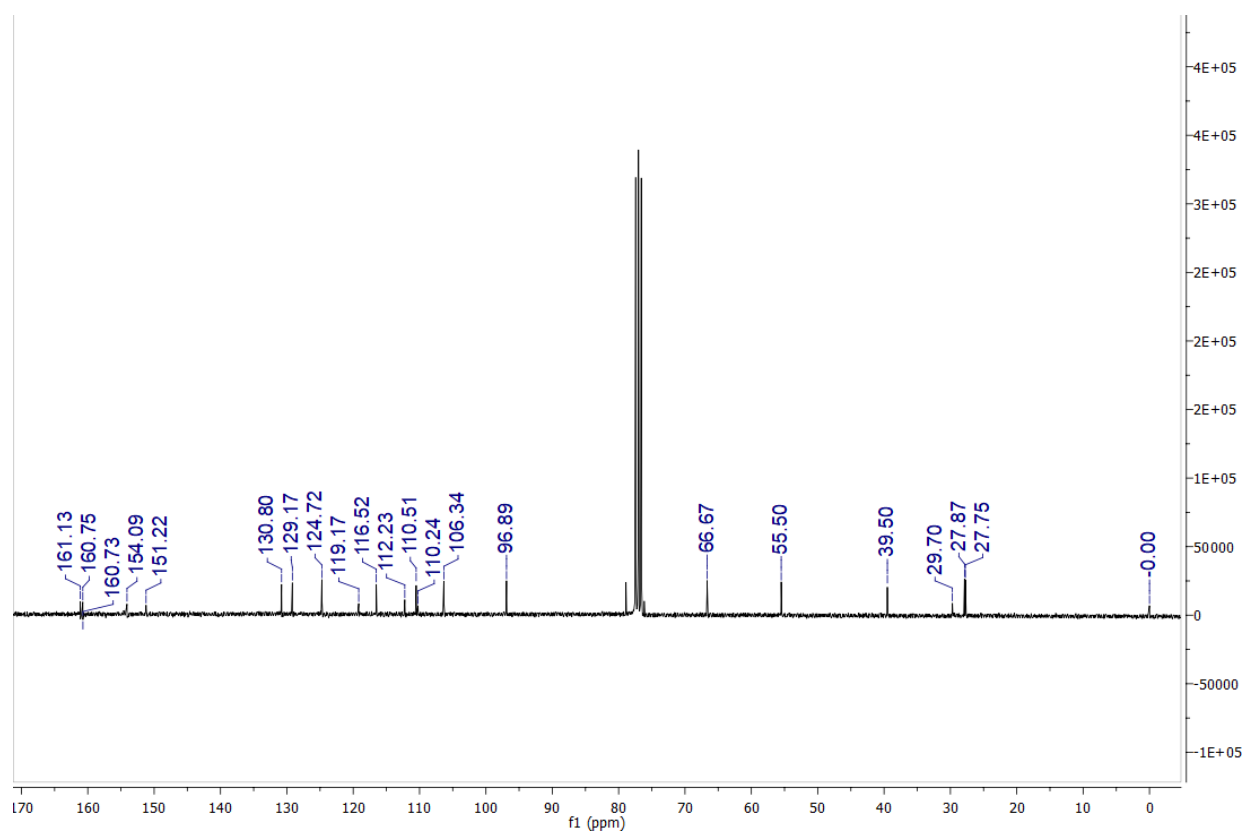

**S4 Fig.** <sup>13</sup>C NMR spectrum (75 MHz, CDCl<sub>3</sub>) of hemileiocarpin (1)

Supplement: S4 Fig — (PDF) [file pone.0241855.s004.pdf]

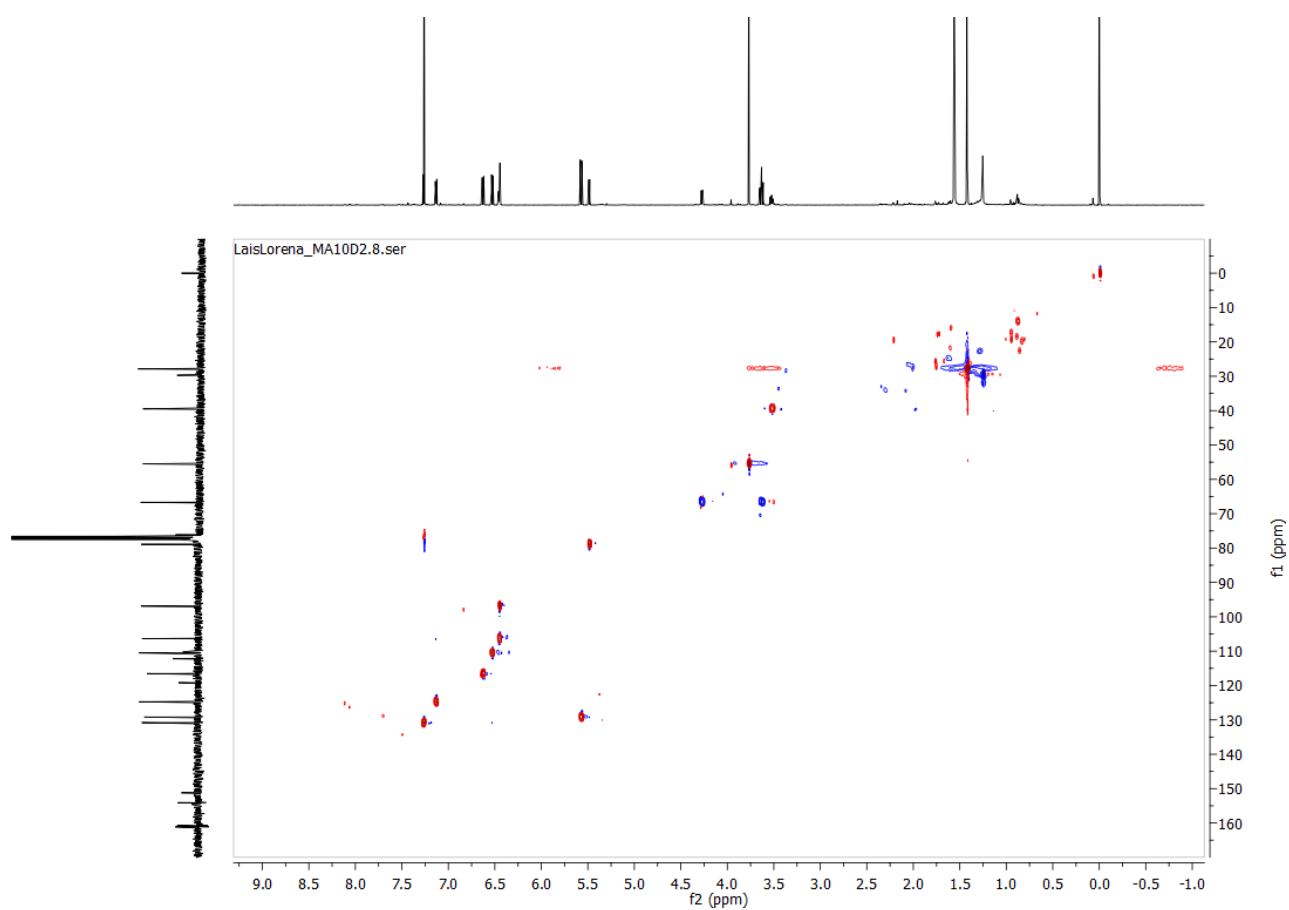

**S5 Fig. Edited HSQC spectrum (CDCl<sub>3</sub>) of hemileiocarpin (1)**

Supplement: S5 Fig — (PDF) [file pone.0241855.s005.pdf]

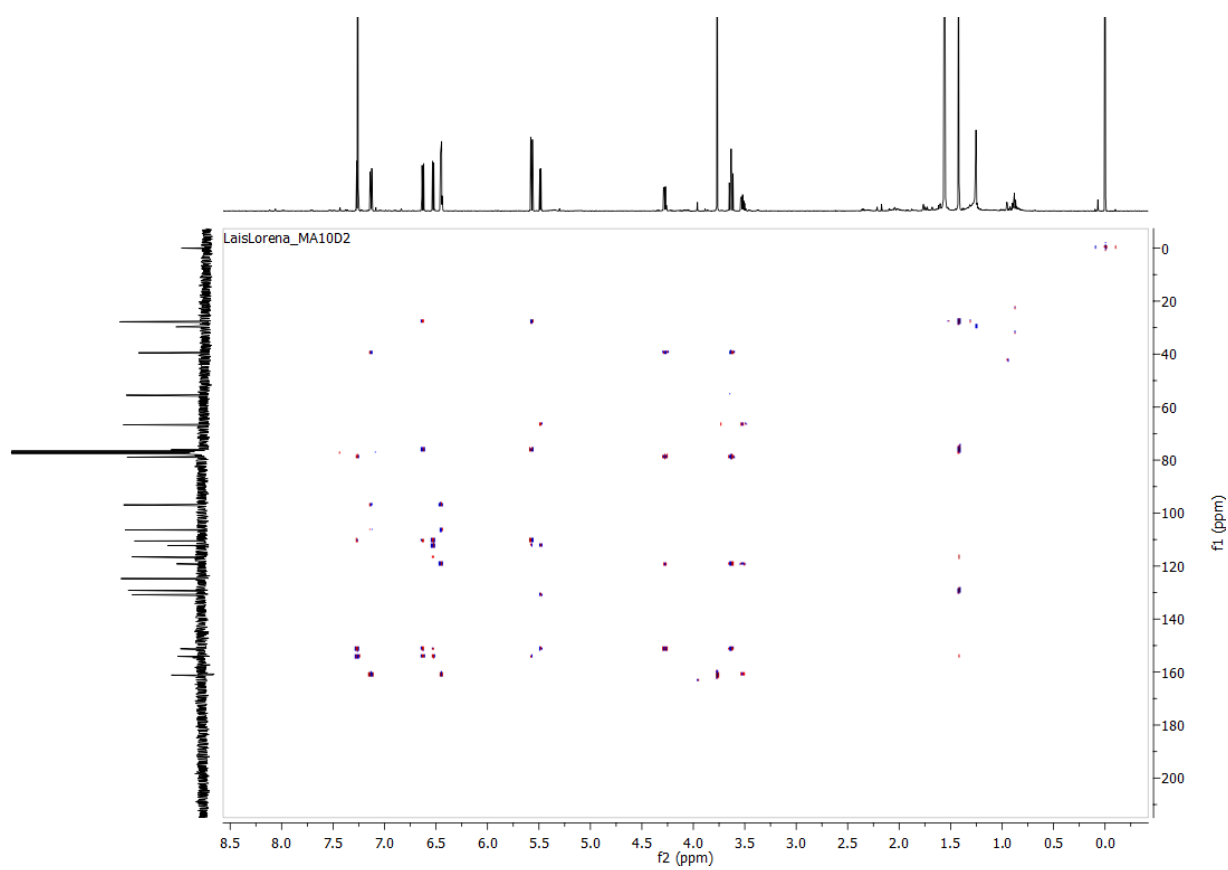

**S6 Fig. HMBC spectrum ( $\text{CDCl}_3$ ) of hemileiocarpin (1)**

Supplement: S6 Fig — (PDF) [file pone.0241855.s006.pdf]

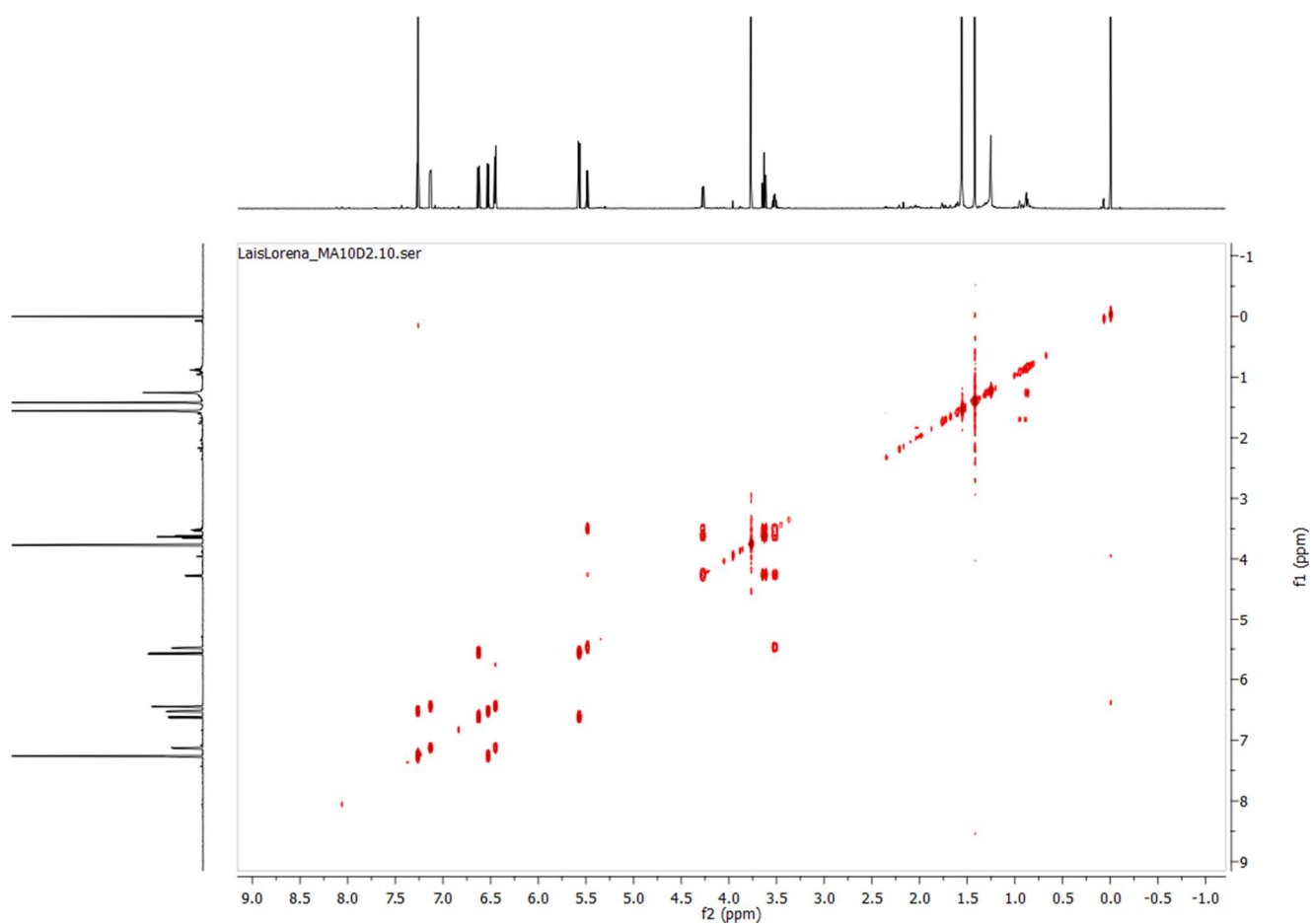

**S7 Fig. COSY spectrum (CDCl<sub>3</sub>) of hemileiocarpin (1)**

Supplement: S7 Fig — (PDF) [file pone.0241855.s007.pdf]

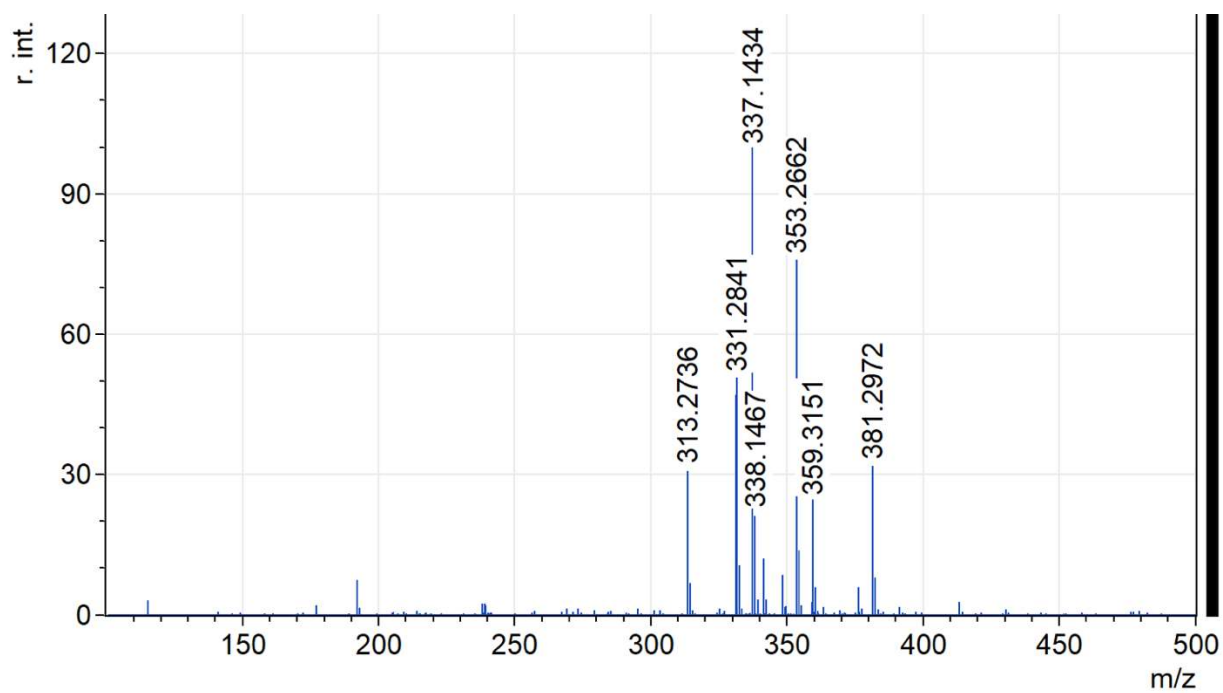

**S8 Fig. HRESIMS spectrum of hemileiocarpin (1)**

Supplement: S8 Fig — (PDF) [file pone.0241855.s008.pdf]

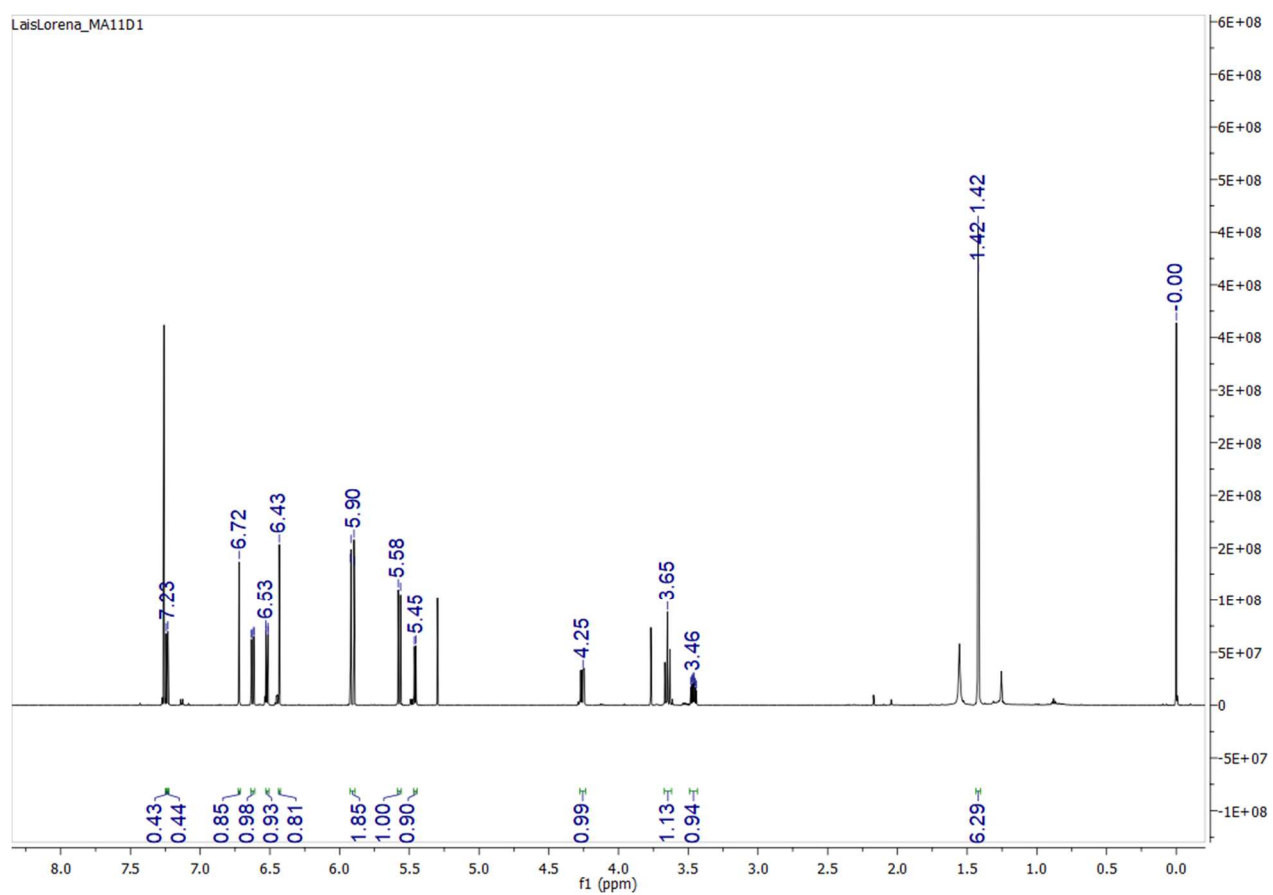

**S9 Fig.  $^1\text{H}$  NMR spectrum (600 MHz  $\text{CDCl}_3$ ) of leiocarpin (2)**

Supplement: S9 Fig — (PDF) [file pone.0241855.s009.pdf]

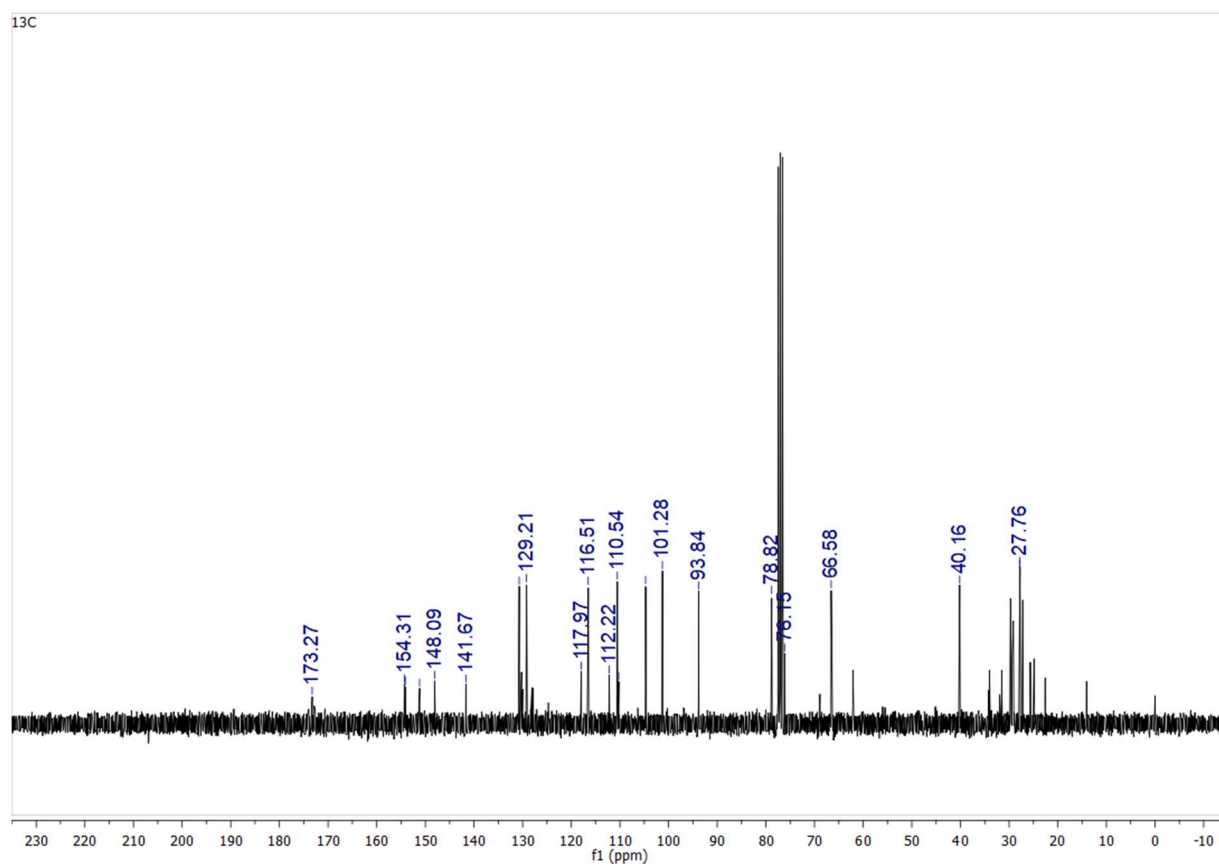

**S10 Fig.** <sup>13</sup>C NMR spectrum (75 MHz, CDCl<sub>3</sub>) of leiocarpin (2)

Supplement: S10 Fig — (PDF) [file pone.0241855.s010.pdf]

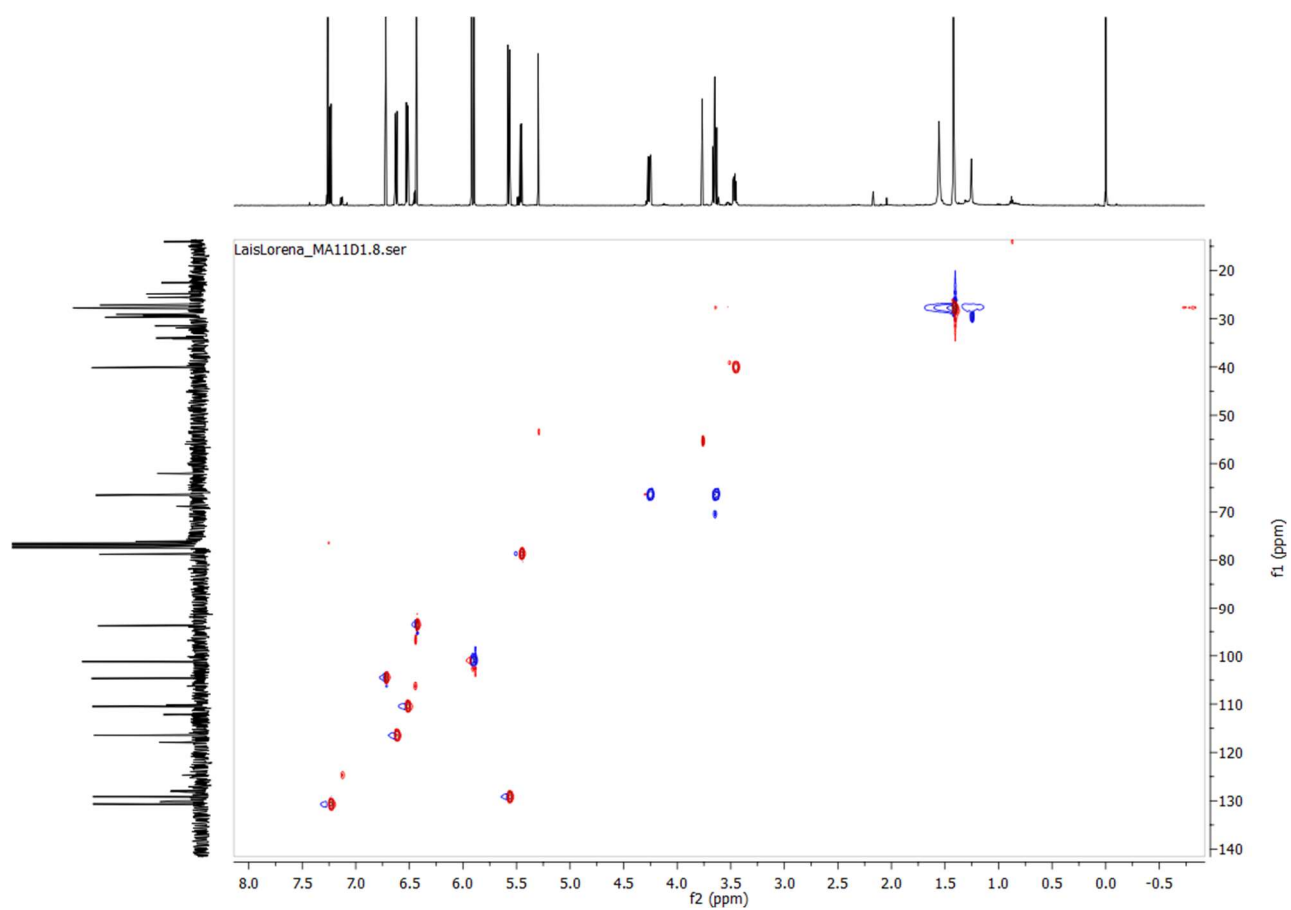

**S11 Fig. Edited HSQC spectrum (CDCl<sub>3</sub>) of leiocarpin (2)**

Supplement: S11 Fig — (PDF) [file pone.0241855.s011.pdf]

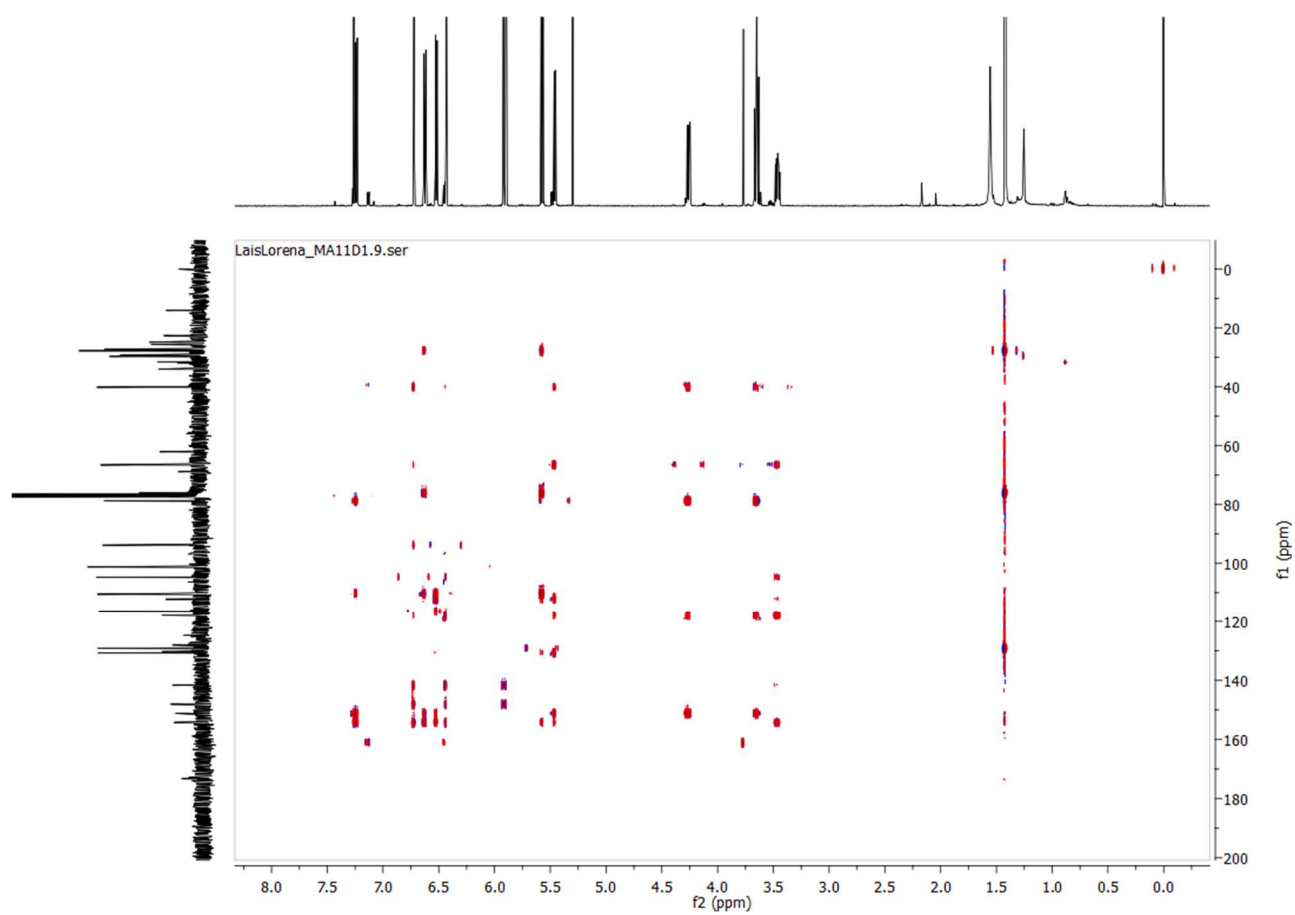

**S12 Fig. HMBC spectrum (CDCl<sub>3</sub>) of leiocarpin (2)**

Supplement: S12 Fig — (PDF) [file pone.0241855.s012.pdf]

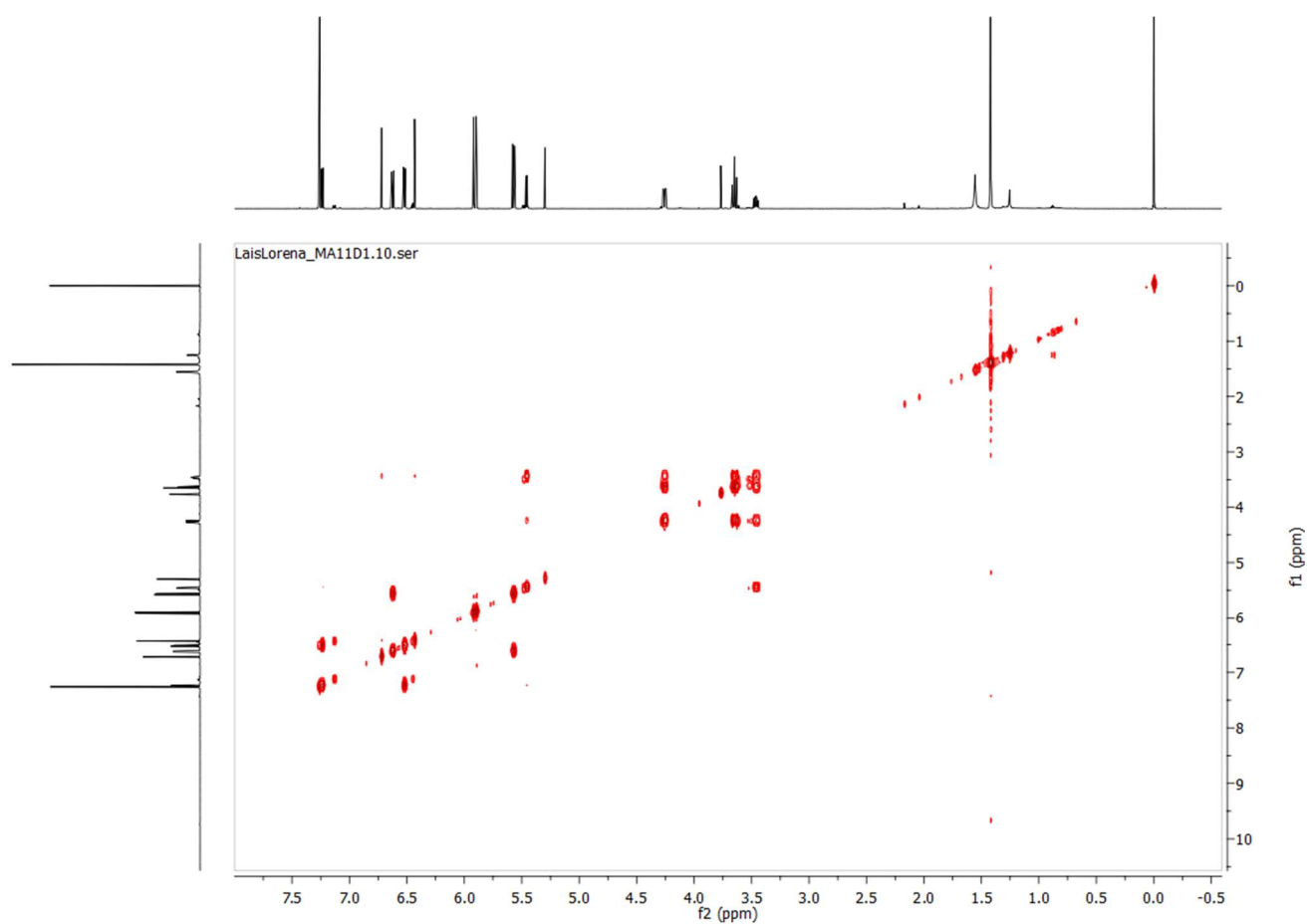

**S13 Fig. COSY spectrum (CDCl<sub>3</sub>) of leiocarpin (2)**

Supplement: S13 Fig — (PDF) [file pone.0241855.s013.pdf]

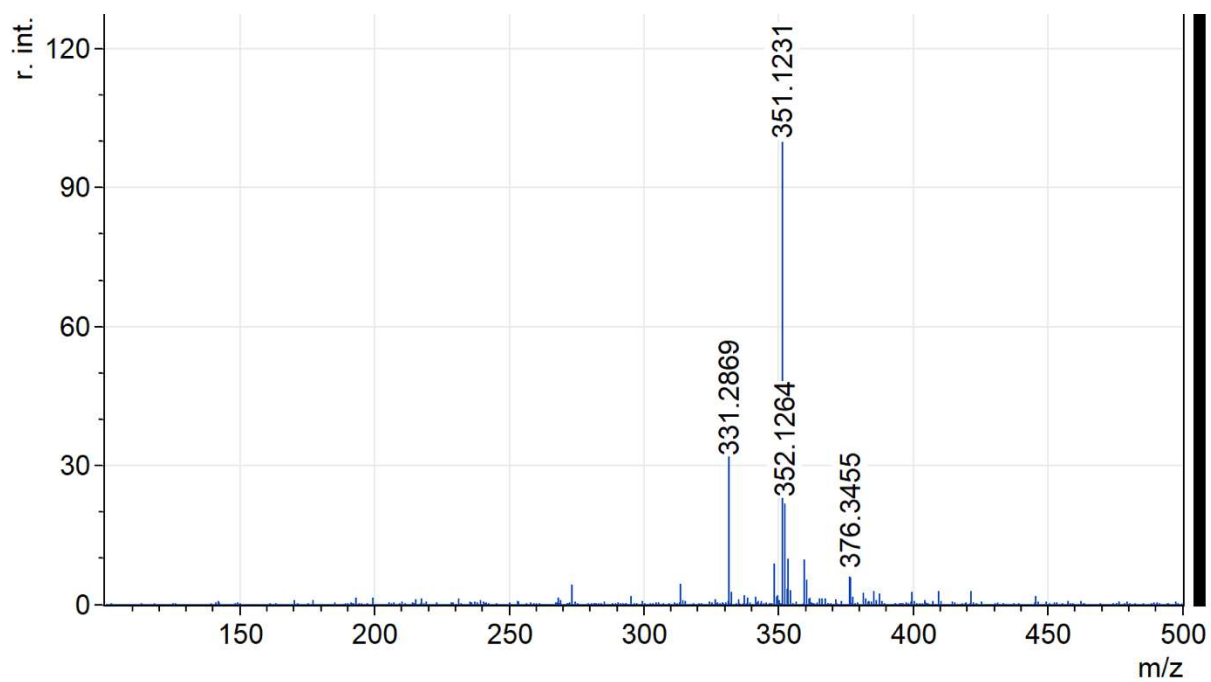

**S14 Fig. HRESIMS spectrum of leiocarpin (2)**

Supplement: S14 Fig — (PDF) [file pone.0241855.s014.pdf]

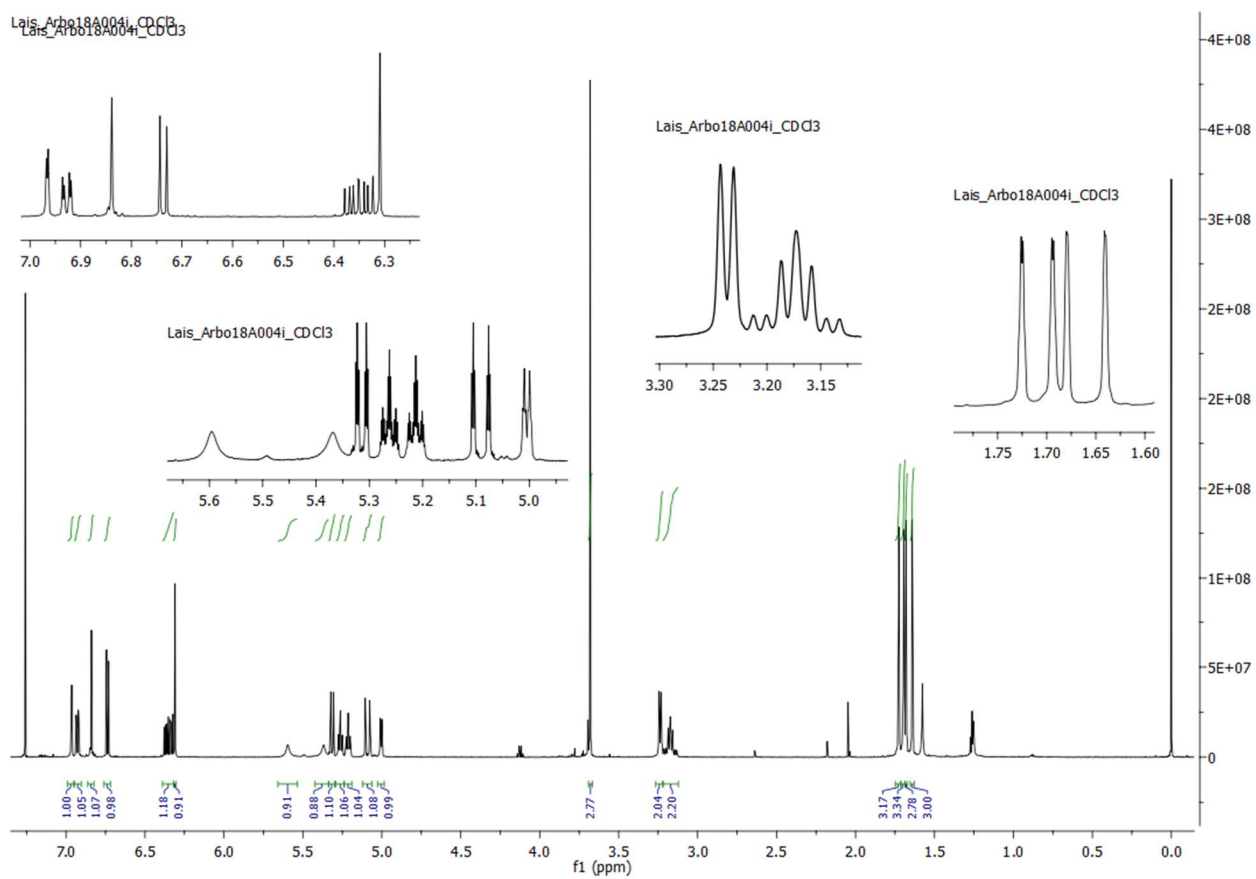

**S15 Fig. <sup>1</sup>H NMR spectrum (600 MHz CDCl<sub>3</sub>) of connarin (3)**

Supplement: S15 Fig — (PDF) [file pone.0241855.s015.pdf]

Lais\_Arbo18A004i\_CDCl3

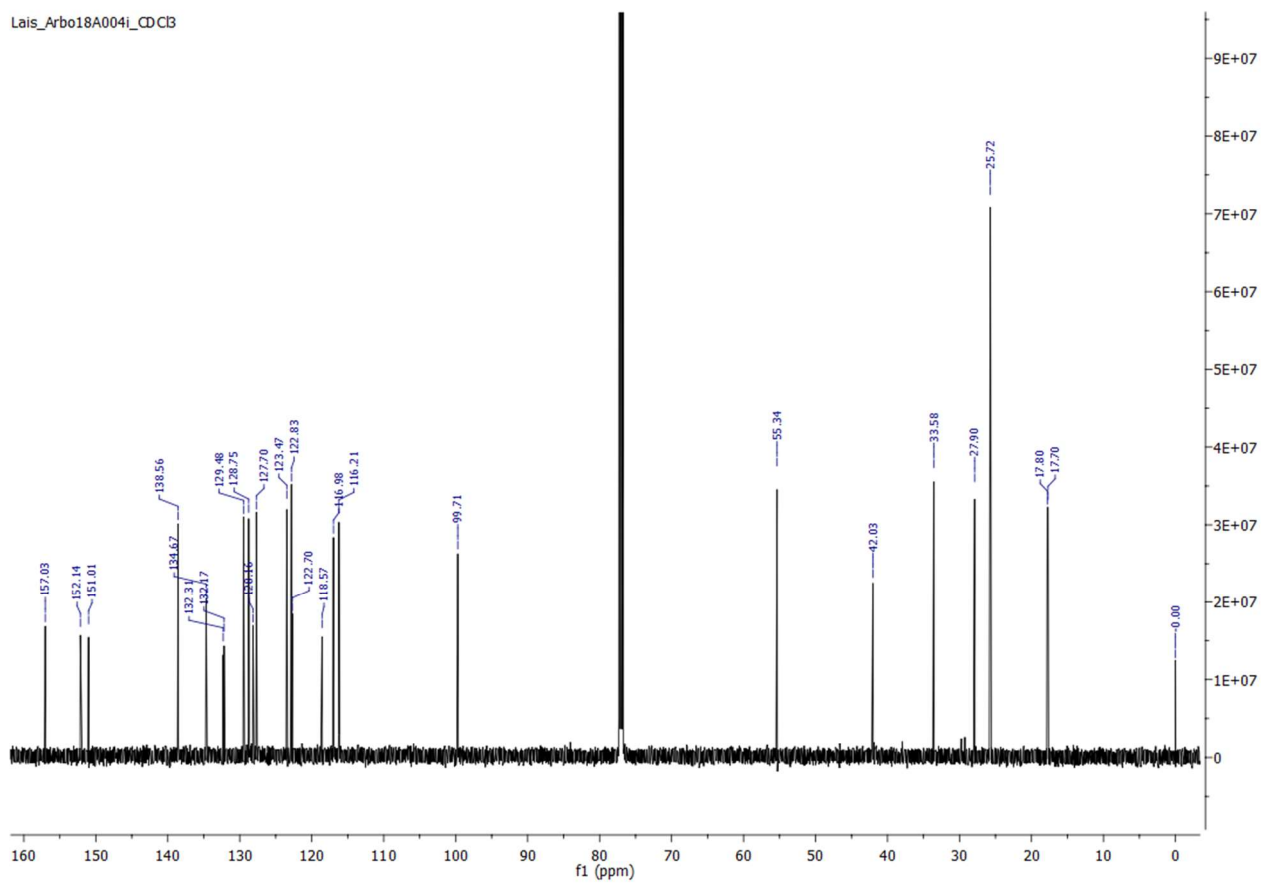

S16 Fig. <sup>13</sup>C NMR spectrum (150 MHz, CDCl<sub>3</sub>) of connarin (3)

Supplement: S16 Fig — (PDF) [file pone.0241855.s016.pdf]

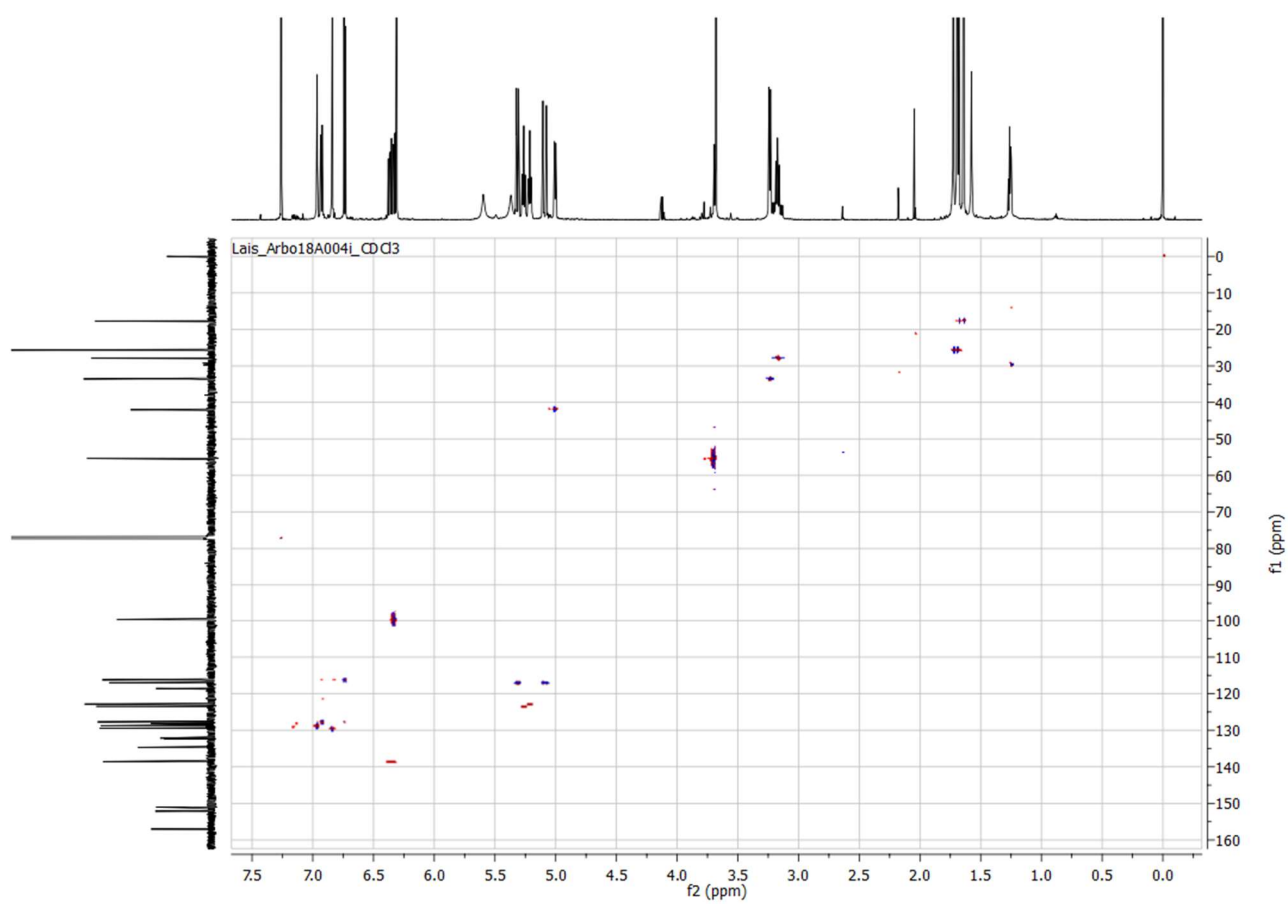

**S17 Fig. Edited HSQC spectrum (CDCl<sub>3</sub>) of connarin (3)**

Supplement: S17 Fig — (PDF) [file pone.0241855.s017.pdf]

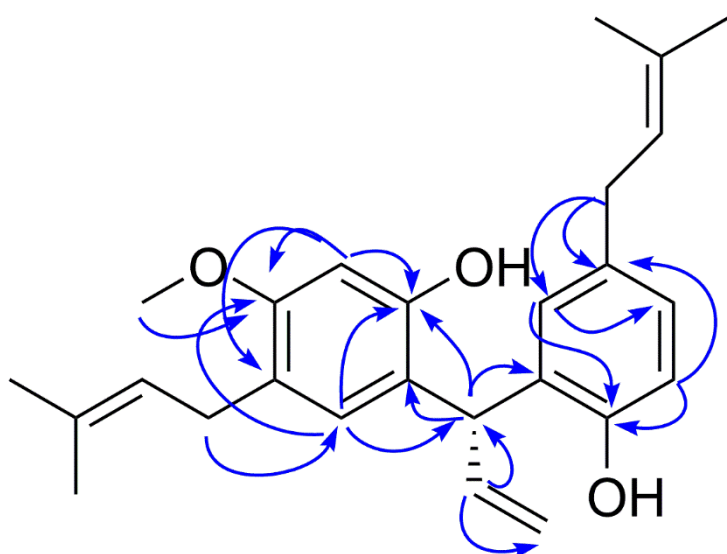

**S18 Fig. HMBC selected correlations of connarin (3)**

Supplement: S18 Fig — (PDF) [file pone.0241855.s018.pdf]

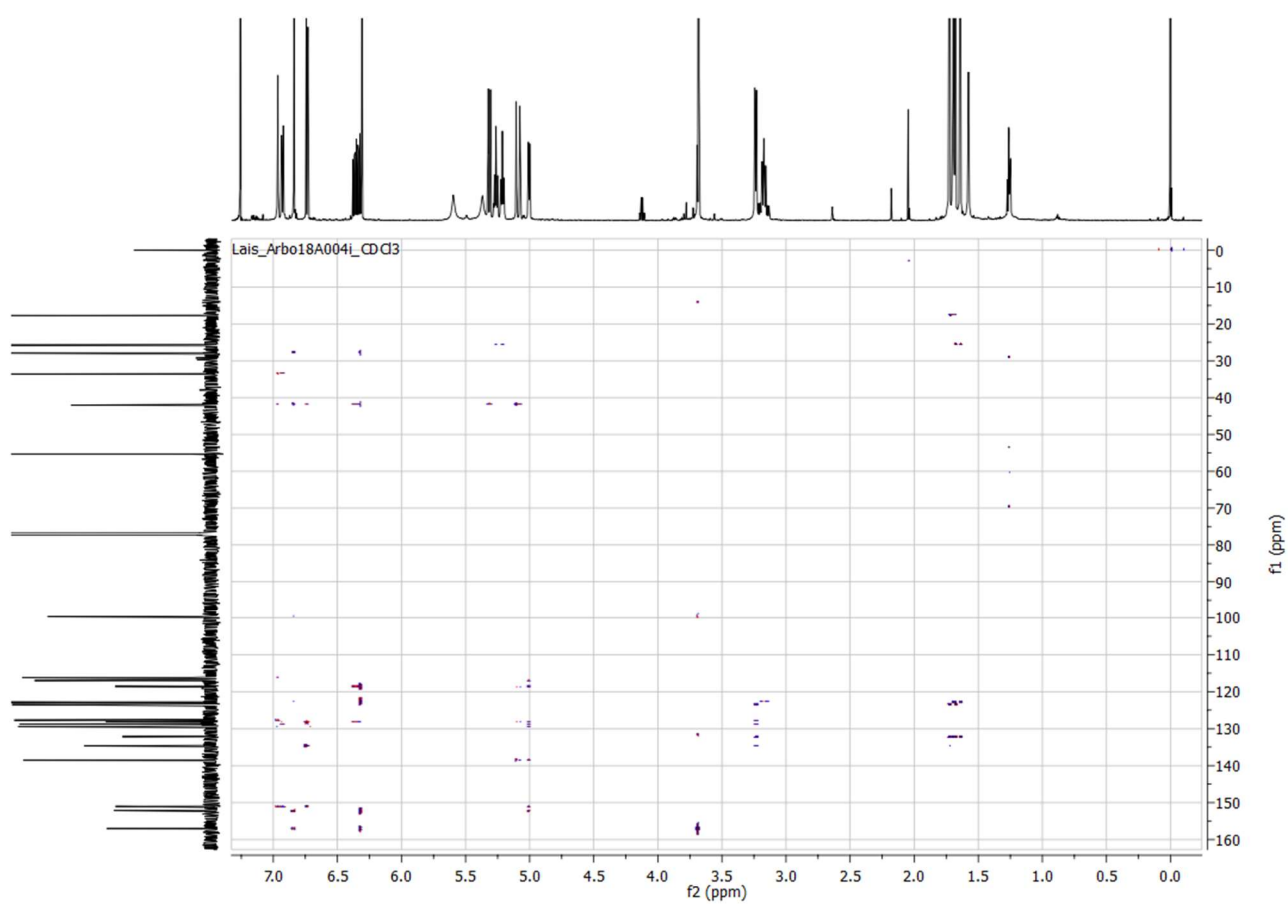

S19 Fig. HMBC spectrum (CDCl<sub>3</sub>) of connarin (3)

Supplement: S19 Fig — (PDF) [file pone.0241855.s019.pdf]

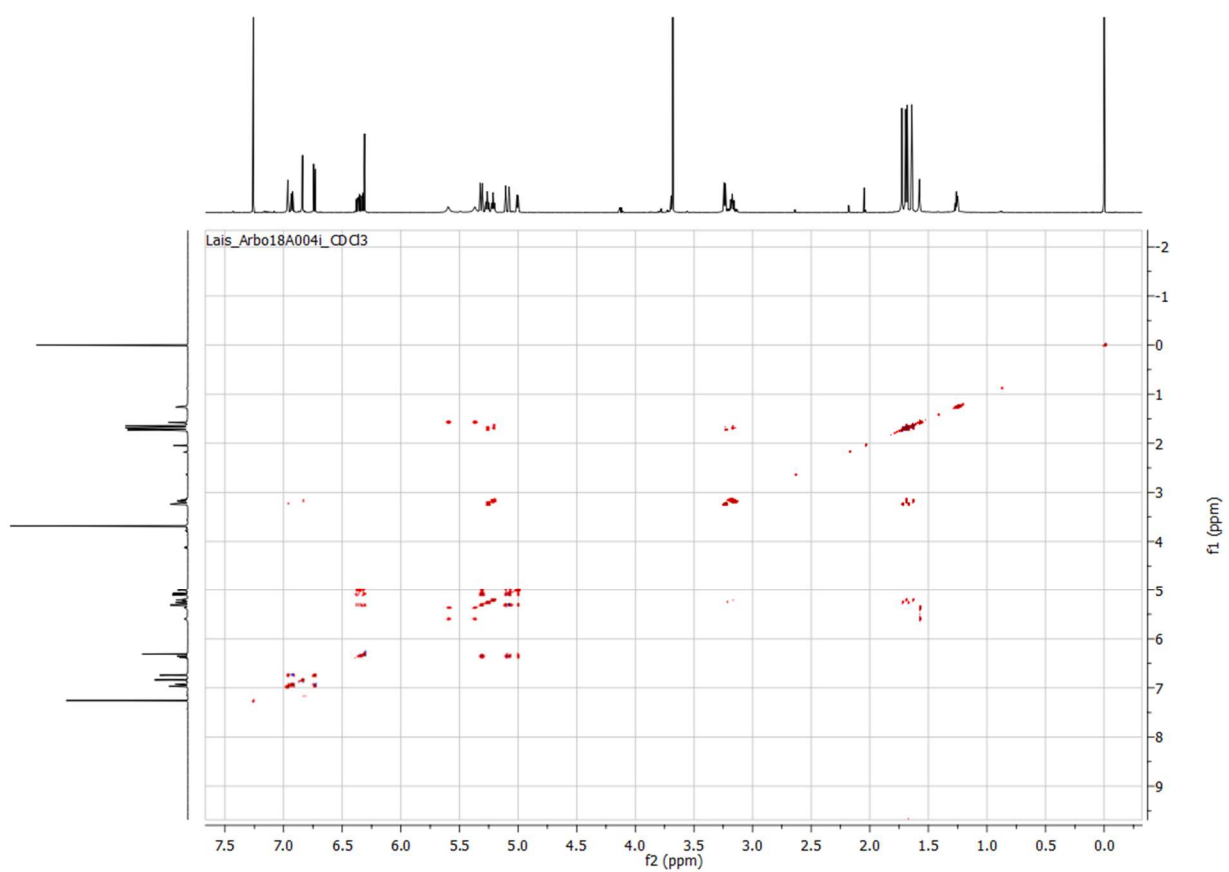

**S20 Fig. COSY spectrum (CDCl<sub>3</sub>) of connarin (3)**

Supplement: S20 Fig — (PDF) [file pone.0241855.s020.pdf]

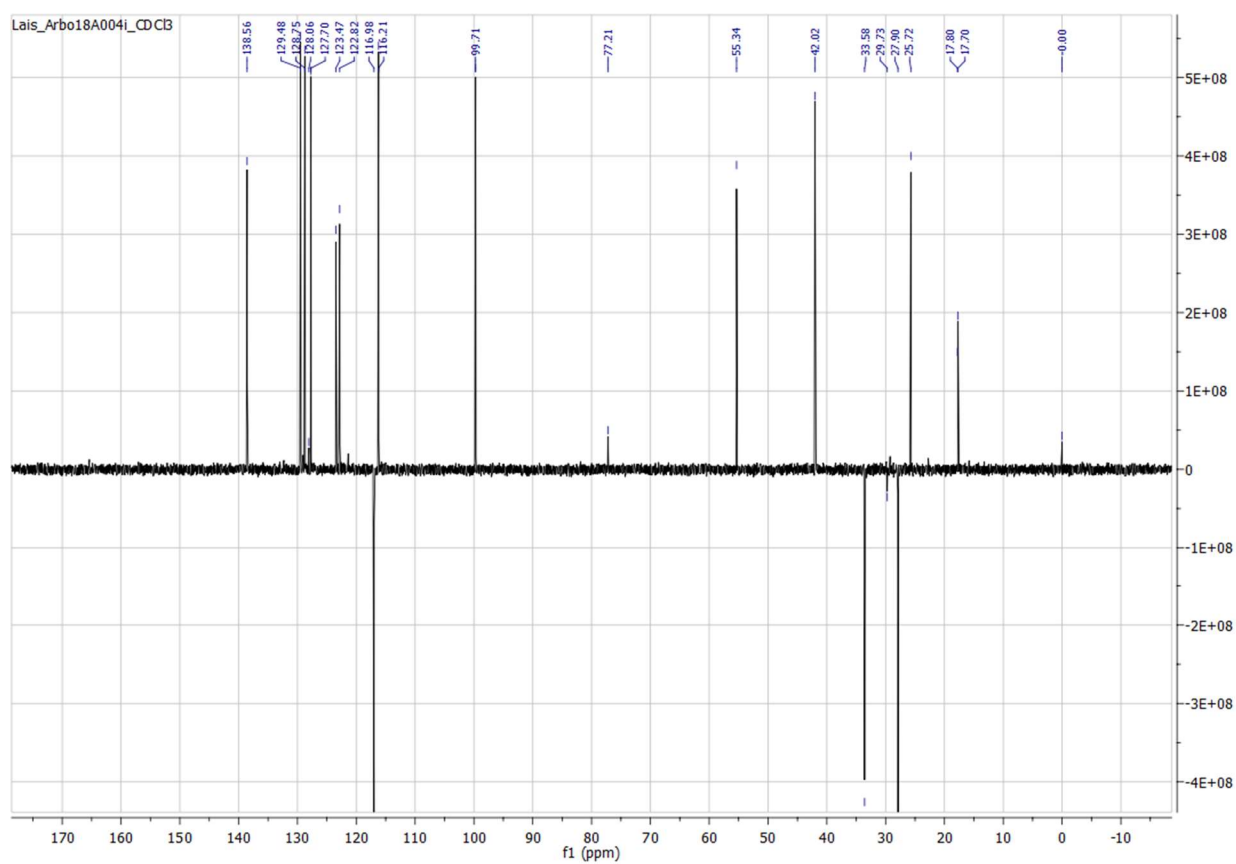

**S21 Fig. DEPT spectrum (150 MHz,  $\text{CDCl}_3$ ) of connarin (3)**

Supplement: S21 Fig — (PDF) [file pone.0241855.s021.pdf]

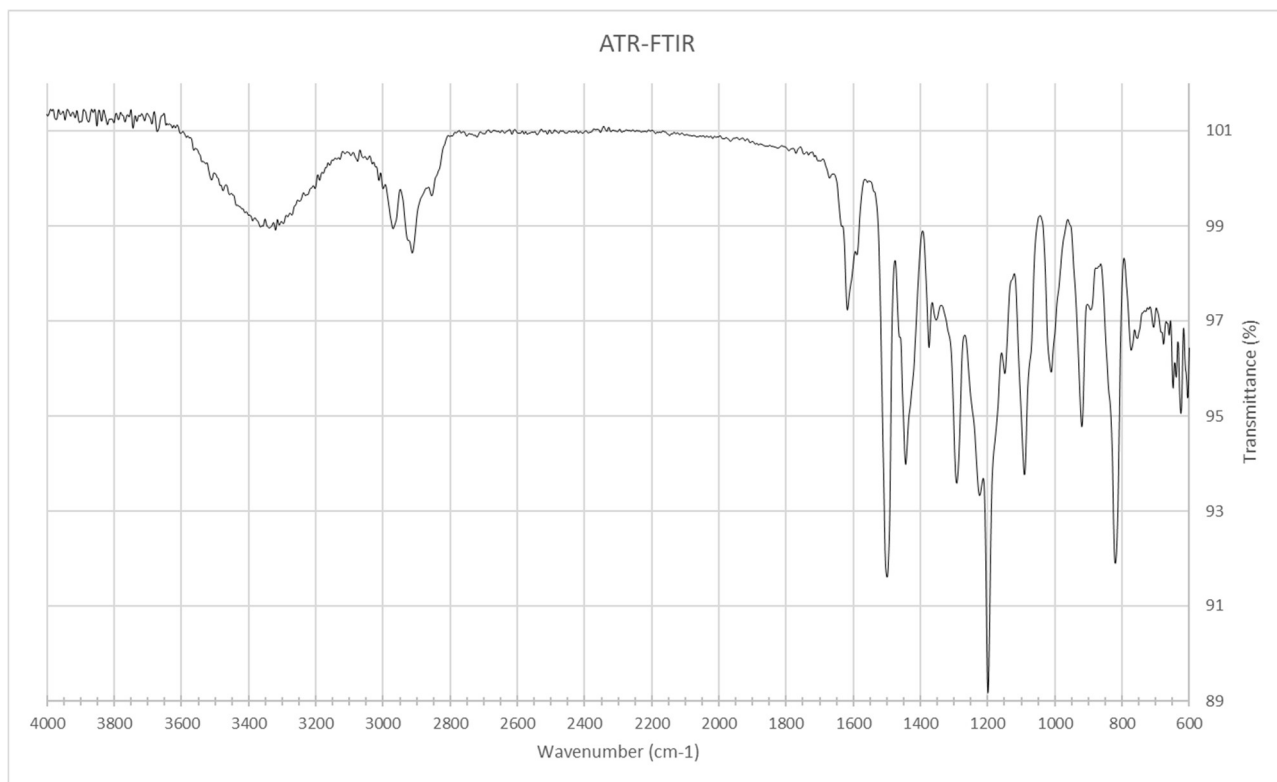

**S22 Fig. IR spectrum of connarin (3)**

Supplement: S22 Fig — (PDF) [file pone.0241855.s022.pdf]

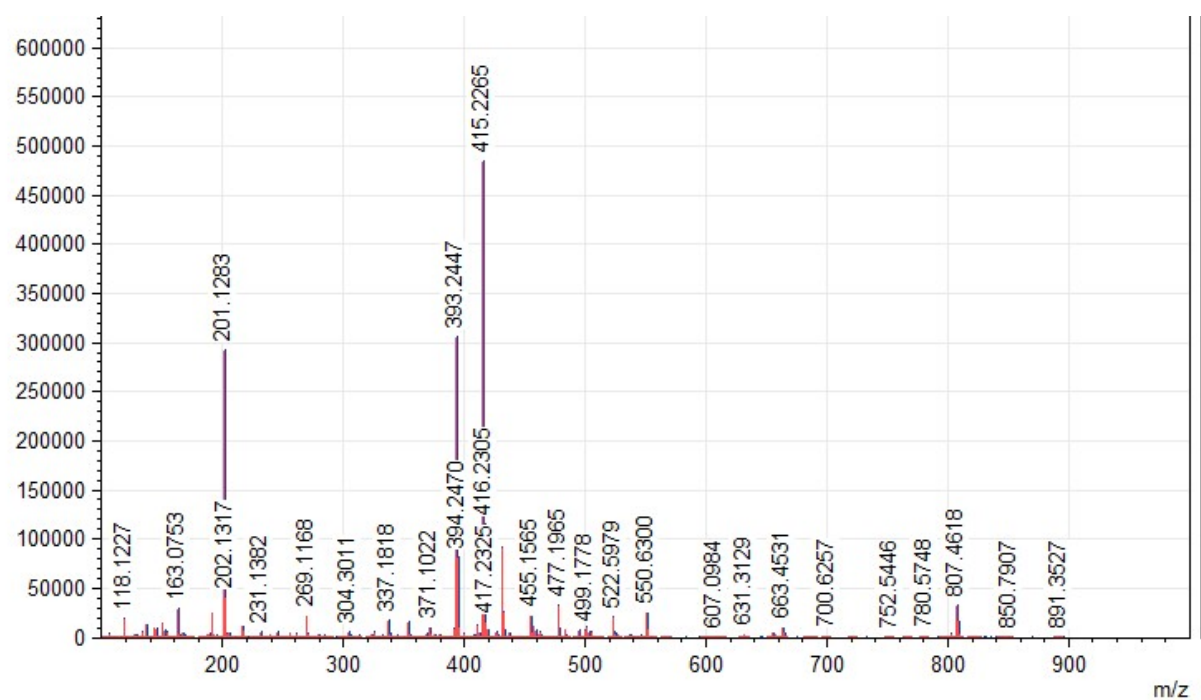

**S23 Fig. HRESIMS spectrum of connarin (3)**

Supplement: S23 Fig — (PDF) [file pone.0241855.s023.pdf]

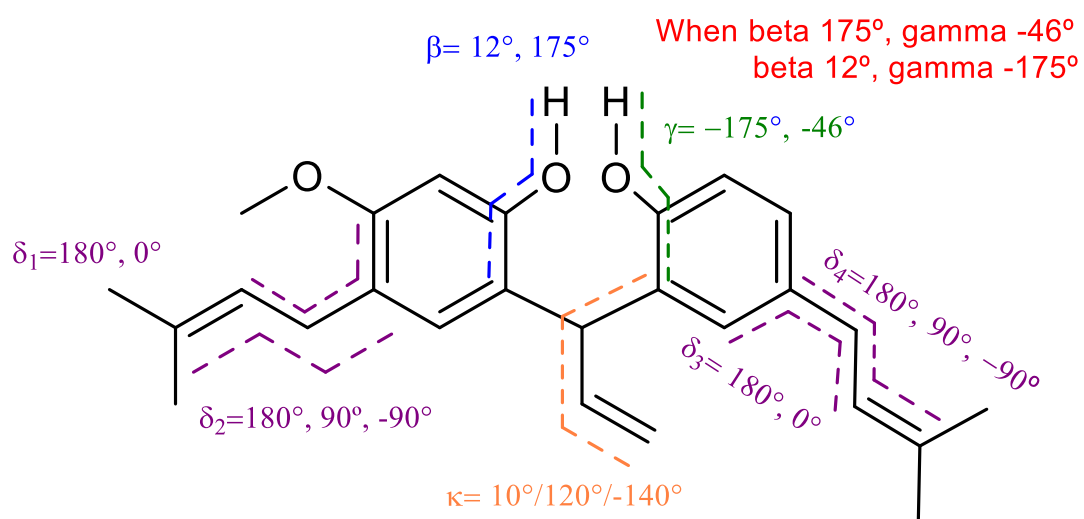

**S24 Fig. Systematic conformational search performed for connarin (3)**

Supplement: S24 Fig — (PDF) [file pone.0241855.s024.pdf]
