# Supplementary material for: Antileishmanial compounds from Connarus suberosus: Metabolomics, isolation and mechanism of action
Source: PLoS One. 2020 Nov 6;15(11):e0241855. doi: 10.1371/journal.pone.0241855 (PMC7647111; doi:10.1371/journal.pone.0241855)
Supplement: S1 Table — (PDF) [file pone.0241855.s025.pdf]

S1 Table. Viability of *L. amazonensis* promastigotes after 24 h exposure to *C. suberosus* crude extracts at 100 µg/mL and 50 µg/mL

**Sample**	***L. amazonensis*** **100 µg/mL**	***L. amazonensis*** **50 µg/mL**
SWH	14.0 (1.22 – 26.72)^S	83.6 (54.0 – 162.20)
SBEtOAc	45.5 (23.98 – 67.0)^S	58.1(28.57 – 87.66)^S
SWEtOAc	79.1 (72.38 – 85.85)	93.7 (91.33 – 95.99)
RBH	10.8 (1.15 – 20.47)^S	15.1 (-3. 071 – 33.16)^S
RWH	83.7 (79.53 – 87.94)	92.8 (85.28 – 100.30)
RWEtOAc	17.7 (14.03 – 21.35)^S	81.1 (50.64 – 111.50)
RBEtOAc	92.2 (88.65 – 95.79)	90.4 (78.16 – 102.60)
LEtOAc	78.9 (60.26 – 97.60)	94.7 (86.08 – 102.20)
RWEtOH	76.9 (65.43 – 88.30)^S	94.0 (90.82 – 97.14)
Amphotericin B	0.00*	0.00*

SW: stem wood, SB: stem bark, RB: root bark, RW: root wood, L: leaf. H: hexane, EtOAc: ethyl acetate, EtOH: ethanol.

Amphotericin B was used as positive control. *The values for Amphotericin B were low comparing to the others, the viability was considered null. ^{S}Samples that are statistically significant ($p < 0.05$) when compared to DMSO in the Dunnet's test comparison of the means.

Data reported as the average of 3 independent experiments performed in duplicate.
