# Supplementary material for: Antileishmanial compounds from Connarus suberosus: Metabolomics, isolation and mechanism of action
Source: PLoS One. 2020 Nov 6;15(11):e0241855. doi: 10.1371/journal.pone.0241855 (PMC7647111; doi:10.1371/journal.pone.0241855)
Supplement: S2 Table — (PDF) [file pone.0241855.s026.pdf]

S2 Table. *In vitro* antileishmanial activity, cytotoxicity in murine peritoneal macrophages, and selectivity index of *C. suberosus* extracts

	Promastigotes $^{a}IC_{50}$		Amastigotes IC_{50}	Cytotoxicity $^{b}CC_{50}$	
**Sample**	***L. amazonensis***	***L. infantum***	***L. amazonensis***	**Murine macrophages**	**[c]SI**
	**µg/mL**	**µg/mL**	**µg/mL**	**µg/mL**	
**RWH**	>100.0	>100.0	>100.0	>100.0	[d]-
**RWEtOAc**	29.9 ± 1.1	21.1 ± 2.0	26.6 (23.3-30.2)	90.8 (65.7-125.4)	3.4
**RWEtOH**	>100.0	>100.0	>100.0	>100.0	-
**RBEtOAc**	74.3 (65.8-84.0)	68.0 ± 6.6	58.6 (50.8-67.7)	>100.0	-
**LEtOAc**	>100.0	>100.0	>100.0	>100.0	-
**SWH**	>100.0	>100.0	>100.0	37.1 (30.7-45.0)	-
**SBEtOAc**	>100.0	>100.0	>100.0	>100.0	-
**SWEtOAc**	>100.0	>100.0	>100.0	>100.0	-
**Miltefosine**	9.0 ± 0.5	2.6 ± 0.3	5.2 (4.8-5.5)	53.6 (49.7-57.6)	10.4

RW: root wood, RB: root bark, L: leaf, SW: stem wood, SB: stem bark. H: hexane, EtOAc: ethyl acetate, EtOH: ethanol.

$^{a}IC_{50}$: Concentration required to inhibit 50% of parasite growth after 72 h exposure.

$^{b}CC_{50}$: Cytotoxic concentration to reduce cell viability by 50% after 72 h exposure.

[c]SI: Selectivity Index.

[d]-Not determined.

Miltefosine was used as reference compound. Data reported as the average of 3 independent experiments performed in duplicate.
