# Supplementary material for: Antileishmanial compounds from Connarus suberosus: Metabolomics, isolation and mechanism of action
Source: PLoS One. 2020 Nov 6;15(11):e0241855. doi: 10.1371/journal.pone.0241855 (PMC7647111; doi:10.1371/journal.pone.0241855)
Supplement: S3 Table — Chemical shifts (ppm) and coupling constants (J, Hz, in parenthesis). (PDF) [file pone.0241855.s027.pdf]

S3 Table. ^{1}H (600 MHz) and ^{13}C (75 MHz) NMR data assignments for hemileiocarpin (1) and leiocarpin (2). Chemical shifts (ppm) and coupling constants (J, Hz, in parenthesis)

$^{\delta}$C Type and position	hemileiocarpin (**1**)		leiocarpin (**2**)	
	**δC**	**δH**	**δC**	**δH**
**CH**				
1	130.8	7.27 (1; d)	130.7	7.24 d (7.0 Hz)
2	110.5	6.53 dd (8.3 Hz; 0.6 Hz)	110.5	6.52 dd (8.5 Hz; 0.7 Hz)
6a	39.5	3.52 m	40.2	3.46 m
8	124.7	7.13 dd (8.7 Hz; 0.5 Hz)		
7	106.3	6.45 m/ 6.46 m	104.7	6.72 bs
10	96.9	6.45 m/ 6.46 m	93.8	6.43 bs
11a	78.9	5.49 bd (11.0 Hz)	78.8	5.46 bd (7.0 Hz)
1'	116.5	6.63 dd (10.0 Hz; 0.6 Hz)	116.5	6.62 dd (10.0 Hz; 0.6 Hz)
2'	129.2	5.57 d (10.0 Hz)	129.2	5.57 d (10.0 Hz)
**CH2**				
			101.3	5.91 dd (14.5 Hz; 1.4 Hz)
6	66.7	3.63 m (11.0 Hz) 4.28 ddd (11.0 Hz; 5.1 Hz; 0.6 Hz)	66.6	4.26 ddd (11 Hz; 5.0 Hz; 0.6 Hz) 3.65 m
**CH3**				
5'	27.8/ 27.9	1.42 bs	27.8	1.42 s
6'	27.8/ 27.9	1.42 bs	27.9	1.42 s
**OCH3**	55.5	3.77 s		
C				
4a	154.1		154.1	
1a	110.2		110.2	
3	151.2		151.2	
7a	119.2		117.9	
9	161.1		148.1	
10a	160.8		154.3	
4	112.2		112.2	
3'	76.1		76.1	
8			141.7	
