# Supplementary material for: Antileishmanial compounds from Connarus suberosus: Metabolomics, isolation and mechanism of action
Source: PLoS One. 2020 Nov 6;15(11):e0241855. doi: 10.1371/journal.pone.0241855 (PMC7647111; doi:10.1371/journal.pone.0241855)
Supplement: S4 Table — (PDF) [file pone.0241855.s028.pdf]

S4 Table. Starting angles of selected conformers for connarin (3)

conformer	β	γ	κ	δ1	δ2	δ3	δ4
g1a_c27	12	-175	10	0	90	180	-90
g1a_c21	12	-175	10	0	180	180	-90
g1a_c19	12	-175	10	0	180	180	180
g1a_c31	12	-175	10	0	-90	180	180
g2a_c15	12	-175	120	180	-90	180	-90
