# Supplementary material for: Antileishmanial compounds from Connarus suberosus: Metabolomics, isolation and mechanism of action
Source: PLoS One. 2020 Nov 6;15(11):e0241855. doi: 10.1371/journal.pone.0241855 (PMC7647111; doi:10.1371/journal.pone.0241855)
Supplement: S1 File — (PDF) [file pone.0241855.s029.pdf]

S1 File. Cartesian coordinates of selected conformers for connarin (3)

Conformer g1a_c27

C	-2.77302	-2.7694	-0.14177
C	-1.57425	-2.73049	0.56523
C	-0.94201	-1.51972	0.8487
C	-1.54128	-0.35296	0.36613
C	-2.7363	-0.35874	-0.34816
C	-3.35122	-1.59042	-0.58676
H	-3.2465	-3.72391	-0.34695
H	-1.05021	0.5948	0.55537
C	0.38433	-1.49281	1.60415
C	0.4029	-0.62052	2.84365
H	1.38963	-0.5284	3.2908
C	-0.62533	-0.03995	3.45076
H	-1.63609	-0.09324	3.06532
H	-0.47955	0.5124	4.37164
O	-0.95201	-3.89346	0.98796
H	-1.45064	-4.66217	0.68357
C	1.55724	-1.19782	0.66832
C	2.08489	0.09171	0.53542
C	2.138	-2.20986	-0.10107
C	3.14874	0.40787	-0.29712
H	1.64368	0.89024	1.12034
C	3.21421	-1.93321	-0.94658
C	3.7145	-0.64119	-1.04575
H	3.62938	-2.75026	-1.5187
O	1.72742	-3.51723	-0.08205
O	4.75733	-0.30005	-1.86101
C	5.38491	-1.32264	-2.62806
H	6.18272	-0.83221	-3.18136
H	5.81106	-2.09653	-1.98397
H	4.68242	-1.77875	-3.33082
H	0.87429	-3.63196	0.36893
H	0.54848	-2.50092	1.99754
C	3.70091	1.81761	-0.43596
H	2.88312	2.46567	-0.77046
H	3.9704	2.1938	0.55465
C	4.84526	1.96234	-1.39899
H	5.82903	1.80825	-0.95897
C	4.79901	2.2312	-2.70768
C	6.06313	2.29548	-3.52716
H	6.95033	2.105	-2.92147
H	6.03946	1.56112	-4.33995
H	6.17383	3.27767	-3.99963
C	3.53873	2.48896	-3.49131
H	2.63908	2.48314	-2.87826
H	3.59733	3.45969	-3.99512
H	3.41638	1.73843	-4.27993
H	-4.28802	-1.63038	-1.13066
C	-3.35316	0.93133	-0.86171
H	-3.39171	0.9031	-1.95278
H	-2.6823	1.75742	-0.60238
C	-4.71794	1.20717	-0.28531
H	-4.7044	1.78658	0.63601
C	-5.9116	0.83398	-0.75822
C	-7.17835	1.2179	-0.03584
H	-7.74696	0.32781	0.25513
H	-6.97297	1.80142	0.8627
H	-7.83504	1.80744	-0.68498
C	-6.13382	0.02876	-2.01154
H	-6.7289	0.6005	-2.73187
H	-5.21096	-0.27205	-2.50464
H	-6.70802	-0.87629	-1.78612

Conformer g1a_c21

C	-2.77302	-2.7694	-0.14177
C	-1.57425	-2.73049	0.56523
C	-0.94201	-1.51972	0.8487
C	-1.54128	-0.35296	0.36613
C	-2.7363	-0.35874	-0.34816
C	-3.35122	-1.59042	-0.58676
H	-3.2465	-3.72391	-0.34695
H	-1.05021	0.5948	0.55537
C	0.38433	-1.49281	1.60415
C	0.4029	-0.62052	2.84365
H	1.38963	-0.5284	3.2908
C	-0.62533	-0.03995	3.45076
H	-1.63609	-0.09324	3.06532
H	-0.47955	0.5124	4.37164
O	-0.95201	-3.89346	0.98796
H	-1.45064	-4.66217	0.68357
C	1.55724	-1.19782	0.66832
C	2.08489	0.09171	0.53542
C	2.138	-2.20986	-0.10107
C	3.14874	0.40787	-0.29712
H	1.64368	0.89024	1.12034
C	3.21421	-1.93321	-0.94658
C	3.7145	-0.64119	-1.04575
H	3.62938	-2.75026	-1.5187
O	1.72742	-3.51723	-0.08205
O	4.75733	-0.30005	-1.86101
C	5.38491	-1.32264	-2.62806
H	6.18272	-0.83221	-3.18136
H	5.81106	-2.09653	-1.98397
H	4.68242	-1.77875	-3.33082
H	0.87429	-3.63196	0.36893
H	0.54848	-2.50092	1.99754
C	3.70091	1.81761	-0.43596
H	2.88312	2.46567	-0.77046
H	3.9704	2.1938	0.55465
C	4.84526	1.96234	-1.39899
H	4.5932	1.74909	-2.43637
C	6.10344	2.33402	-1.14221
C	7.11152	2.46554	-2.25583
H	6.67821	2.22256	-3.22715
H	7.50948	3.48525	-2.304
H	7.96939	1.80539	-2.08725
C	6.64585	2.65521	0.22581
H	5.91916	2.51294	1.02388
H	7.51578	2.02723	0.44584
H	6.99542	3.69267	0.26666
H	-4.28802	-1.63038	-1.13066
C	-3.35316	0.93133	-0.86171
H	-3.39171	0.9031	-1.95278
H	-2.6823	1.75742	-0.60238
C	-4.71794	1.20717	-0.28531
H	-4.7044	1.78658	0.63601
C	-5.9116	0.83398	-0.75822
C	-7.17835	1.2179	-0.03584
H	-7.74696	0.32781	0.25513
H	-6.97297	1.80142	0.8627
H	-7.83504	1.80744	-0.68498
C	-6.13382	0.02876	-2.01154
H	-6.7289	0.6005	-2.73187
H	-5.21096	-0.27205	-2.50464
H	-6.70802	-0.87629	-1.78612

Conformer g1a_c19

C	-2.77302	-2.7694	-0.14177
C	-1.57425	-2.73049	0.56523
C	-0.94201	-1.51972	0.8487
C	-1.54128	-0.35296	0.36613
C	-2.7363	-0.35874	-0.34816
C	-3.35122	-1.59042	-0.58676
H	-3.2465	-3.72391	-0.34695
H	-1.05021	0.5948	0.55537
C	0.38433	-1.49281	1.60415
C	0.4029	-0.62052	2.84365
H	1.38963	-0.5284	3.2908
C	-0.62533	-0.03995	3.45076
H	-1.63609	-0.09324	3.06532
H	-0.47955	0.5124	4.37164
O	-0.95201	-3.89346	0.98796
H	-1.45064	-4.66217	0.68357
C	1.55724	-1.19782	0.66832
C	2.08489	0.09171	0.53542
C	2.138	-2.20986	-0.10107
C	3.14874	0.40787	-0.29712
H	1.64368	0.89024	1.12034
C	3.21421	-1.93321	-0.94658
C	3.7145	-0.64119	-1.04575
H	3.62938	-2.75026	-1.5187
O	1.72742	-3.51723	-0.08205
O	4.75733	-0.30005	-1.86101
C	5.38491	-1.32264	-2.62806
H	6.18272	-0.83221	-3.18136
H	5.81106	-2.09653	-1.98397
H	4.68242	-1.77875	-3.33082
H	0.87429	-3.63196	0.36893
H	0.54848	-2.50092	1.99754
C	3.70091	1.81761	-0.43596
H	2.88312	2.46567	-0.77046
H	3.9704	2.1938	0.55465
C	4.84526	1.96234	-1.39899
H	4.5932	1.74909	-2.43637
C	6.10344	2.33402	-1.14221
C	7.11152	2.46554	-2.25583
H	6.67821	2.22256	-3.22715
H	7.50948	3.48525	-2.304
H	7.96939	1.80539	-2.08725
C	6.64585	2.65521	0.22581
H	5.91916	2.51294	1.02388
H	7.51578	2.02723	0.44584
H	6.99542	3.69267	0.26666
H	-4.28802	-1.63038	-1.13066
C	-3.35316	0.93133	-0.86171
H	-3.39171	0.9031	-1.95278
H	-2.6823	1.75742	-0.60238
C	-4.71794	1.20717	-0.28531
H	-4.74251	1.25122	0.80198
C	-5.87159	1.39836	-0.93358
C	-7.14419	1.67248	-0.17252
H	-7.90264	0.91349	-0.39376
H	-6.97818	1.68604	0.90558
H	-7.57475	2.63635	-0.46537
C	-6.03919	1.37176	-2.43018
H	-6.40211	2.34035	-2.79073
H	-5.12102	1.13828	-2.96661
H	-6.79561	0.63292	-2.71577

Conformer g1a_c31

C	-2.77302	-2.7694	-0.14177
C	-1.57425	-2.73049	0.56523
C	-0.94201	-1.51972	0.8487
C	-1.54128	-0.35296	0.36613
C	-2.7363	-0.35874	-0.34816
C	-3.35122	-1.59042	-0.58676
H	-3.2465	-3.72391	-0.34695
H	-1.05021	0.5948	0.55537
C	0.38433	-1.49281	1.60415
C	0.4029	-0.62052	2.84365
H	1.38963	-0.5284	3.2908
C	-0.62533	-0.03995	3.45076
H	-1.63609	-0.09324	3.06532
H	-0.47955	0.5124	4.37164
O	-0.95201	-3.89346	0.98796
H	-1.45064	-4.66217	0.68357
C	1.55724	-1.19782	0.66832
C	2.08489	0.09171	0.53542
C	2.138	-2.20986	-0.10107
C	3.14874	0.40787	-0.29712
H	1.64368	0.89024	1.12034
C	3.21421	-1.93321	-0.94658
C	3.7145	-0.64119	-1.04575
H	3.62938	-2.75026	-1.5187
O	1.72742	-3.51723	-0.08205
O	4.75733	-0.30005	-1.86101
C	5.38491	-1.32264	-2.62806
H	6.18272	-0.83221	-3.18136
H	5.81106	-2.09653	-1.98397
H	4.68242	-1.77875	-3.33082
H	0.87429	-3.63196	0.36893
H	0.54848	-2.50092	1.99754
C	3.70091	1.81761	-0.43596
H	4.79452	1.7495	-0.44052
H	3.43944	2.20694	-1.42363
C	3.28953	2.77329	0.64818
H	3.97104	2.81605	1.49605
C	2.22061	3.57525	0.68522
C	1.99457	4.50611	1.84973
H	2.7836	4.41978	2.59824
H	1.95349	5.54838	1.51435
H	1.03511	4.29871	2.33614
C	1.15968	3.6483	-0.38151
H	1.31288	2.9395	-1.19354
H	0.17232	3.45644	0.05191
H	1.11747	4.65431	-0.81316
H	-4.28802	-1.63038	-1.13066
C	-3.35316	0.93133	-0.86171
H	-3.39171	0.9031	-1.95278
H	-2.6823	1.75742	-0.60238
C	-4.71794	1.20717	-0.28531
H	-4.7044	1.78658	0.63601
C	-5.9116	0.83398	-0.75822
C	-7.17835	1.2179	-0.03584
H	-7.74696	0.32781	0.25513
H	-6.97297	1.80142	0.8627
H	-7.83504	1.80744	-0.68498
C	-6.13382	0.02876	-2.01154
H	-6.7289	0.6005	-2.73187
H	-5.21096	-0.27205	-2.50464
H	-6.70802	-0.87629	-1.78612

Conformer g1a_c15

C	-2.77302	-2.7694	-0.14177
C	-1.57425	-2.73049	0.56523
C	-0.94201	-1.51972	0.8487
C	-1.54128	-0.35296	0.36613
C	-2.7363	-0.35874	-0.34816
C	-3.35122	-1.59042	-0.58676
H	-3.2465	-3.72391	-0.34695
H	-1.05021	0.5948	0.55537
C	0.38433	-1.49281	1.60415
C	0.4029	-0.62052	2.84365
H	0.25208	0.43479	2.63001
C	0.64135	-0.98607	4.09761
H	0.80492	-2.01792	4.38323
H	0.68399	-0.24536	4.88748
O	-0.95201	-3.89346	0.98796
H	-1.45064	-4.66217	0.68357
C	1.55724	-1.19782	0.66832
C	2.08489	0.09171	0.53542
C	2.138	-2.20986	-0.10107
C	3.14874	0.40787	-0.29712
H	1.64368	0.89024	1.12034
C	3.21421	-1.93321	-0.94658
C	3.7145	-0.64119	-1.04575
H	3.62938	-2.75026	-1.5187
O	1.72742	-3.51723	-0.08205
O	4.75733	-0.30005	-1.86101
C	5.38491	-1.32264	-2.62806
H	6.18272	-0.83221	-3.18136
H	5.81106	-2.09653	-1.98397
H	4.68242	-1.77875	-3.33082
H	0.87429	-3.63196	0.36893
H	0.54848	-2.50092	1.99754
C	3.70091	1.81761	-0.43596
H	4.79452	1.7495	-0.44052
H	3.43944	2.20694	-1.42363
C	3.28953	2.77329	0.64818
H	3.97104	2.81605	1.49605
C	2.22061	3.57525	0.68522
C	1.99457	4.50611	1.84973
H	2.7836	4.41978	2.59824
H	1.95349	5.54838	1.51435
H	1.03511	4.29871	2.33614
C	1.15968	3.6483	-0.38151
H	1.31288	2.9395	-1.19354
H	0.17232	3.45644	0.05191
H	1.11747	4.65431	-0.81316
H	-4.28802	-1.63038	-1.13066
C	-3.35316	0.93133	-0.86171
H	-3.39171	0.9031	-1.95278
H	-2.6823	1.75742	-0.60238
C	-4.71794	1.20717	-0.28531
H	-4.7044	1.78658	0.63601
C	-5.9116	0.83398	-0.75822
C	-7.17835	1.2179	-0.03584
H	-7.74696	0.32781	0.25513
H	-6.97297	1.80142	0.8627
H	-7.83504	1.80744	-0.68498
C	-6.13382	0.02876	-2.01154
H	-6.7289	0.6005	-2.73187
H	-5.21096	-0.27205	-2.50464
H	-6.70802	-0.87629	-1.78612
