# Supplementary material for: Antileishmanial compounds from Connarus suberosus: Metabolomics, isolation and mechanism of action
Source: PLoS One. 2020 Nov 6;15(11):e0241855. doi: 10.1371/journal.pone.0241855 (PMC7647111; doi:10.1371/journal.pone.0241855)
Supplement: S1 Data — (PDF) [file pone.0241855.s030.pdf]

Supporting information

Antileishmanial compounds from *Connarus suberosus*: metabolomics, isolation and mechanism of action

Lais S. Morais[1], Renata G. Dusi[1], Daniel P. Demarque[1,3], Raquel L. Silva[1], Lorena C. Albernaz[1], Sônia N. Báo[2], Christian Merten[3], Luciana M. R. Antinarelli[4], Elaine S. Coimbra[4], Laila S. Espindola[1*]

[1] Laboratório de Farmacognosia, Universidade de Brasília, Campus Universitário Darcy Ribeiro, Asa Norte, Brasília, DF, Brazil

[2] Laboratório de Microscopia e Microanálise, Instituto de Ciências Biológicas, Universidade de Brasília, Campus Universitário Darcy Ribeiro, Asa Norte, Brasília, DF, Brazil

[3] Fakultät für Chemie und Biochemie, Organische Chemie II, Ruhr-Universität Bochum, Bochum, Germany

[4] Departamento de Parasitologia, Microbiologia e Imunologia, I.C.B., Universidade Federal de Juiz de Fora, Campus Universitário Juiz de Fora, Minas Gerais, Brazil

*** Corresponding author**

E-mail: darvenne@unb.br (LSE)

TABLE OF CONTENTS

S1 Table. Viability of *L. amazonensis* promastigotes after 24 h exposure to *C. suberosus* crude extracts at 100 µg/mL and 50 µg/mL 3
S2 Table. *In vitro* antileishmanial activity, cytotoxicity in murine peritoneal macrophages, and selectivity index of *C. suberosus* extracts 4
S3 Table. ^{1}H (600 MHz) and ^{13}C (75 MHz) NMR data assignments for hemileiocarpin (1) and leiocarpin (2). Chemical shifts (ppm) and coupling constants (J, Hz, in parenthesis) 5
S4 Table. Starting angles of selected conformers for connarin (3) 30

S1 Fig. Activity of silica column fractions of *C. suberosus* root bark hexane (RBH) extract. (A) Inhibitory effect of fractions A17-A27 (100 µg/mL) against *L. amazonensis* promastigotes. *Statistically significant ($p < 0.05$) when compared to the DMSO negative control using the Dunnett's test. **(B)** Dose-response curve of connarin (**3**) against *L. amazonensis* promastigotes after 24 h exposure 6
S2 Fig. Vibrational circular dichroism and infrared experimental (exptl) spectra of connarin (3) in comparison with calculated (calc) spectra for (S) configuration 7
S3 Fig. ^{1}H NMR spectrum (600 MHz, $CDCl_3$) of hemileiocarpin (1) 8
S4 Fig. ^{13}C NMR spectrum (75 MHz, $CDCl_3$) of hemileiocarpin (1) 9
S5 Fig. Edited HSQC spectrum ($CDCl_3$) of hemileiocarpin (1) 10
S6 Fig. HMBC spectrum ($CDCl_3$) of hemileiocarpin (1) 11
S7 Fig. COSY spectrum ($CDCl_3$) of hemileiocarpin (1) 12
S8 Fig. HRESIMS spectrum of hemileiocarpin (1) 13
S9 Fig. ^{1}H NMR spectrum (600 MHz $CDCl_3$) of leiocarpin (2) 14
S10 Fig. ^{13}C NMR spectrum (75 MHz, $CDCl_3$) of leiocarpin (2) 15
S11 Fig. Edited HSQC spectrum ($CDCl_3$) of leiocarpin (2) 16
S12 Fig. HMBC spectrum ($CDCl_3$) of leiocarpin (2) 17
S13 Fig. COSY spectrum ($CDCl_3$) of leiocarpin (2) 18
S14 Fig. HRESIMS spectrum of leiocarpin (2) 19
S15 Fig. ^{1}H NMR spectrum (600 MHz $CDCl_3$) of connarin (3) 20
S16 Fig. ^{13}C NMR spectrum (150 MHz, $CDCl_3$) of connarin (3) 21
S17 Fig. Edited HSQC spectrum ($CDCl_3$) of connarin (3) 22
S18 Fig. HMBC selected correlations of connarin (3) 23
S19 Fig. HMBC spectrum ($CDCl_3$) of connarin (3) 24
S20 Fig. COSY spectrum ($CDCl_3$) of connarin (3) 25
S21 Fig. DEPT spectrum (150 MHz, $CDCl_3$) of connarin (3) 26
S22 Fig. IR spectrum of connarin (3) 27
S23 Fig. HRESIMS spectrum of connarin (3) 28
S24 Fig. Systematic conformational search performed for connarin (3) 29

S1 File. Cartesian coordinates of selected conformers for connarin (3) 31

S1 Table. Viability of *L. amazonensis* promastigotes after 24 h exposure to *C. suberosus* crude extracts at 100 µg/mL and 50 µg/mL

Sample	*L. amazonensis* 100 µg/mL	*L. amazonensis* 50 µg/mL
SWH	14.0 (1.22 – 26.72)[S]	83.6 (54.0 – 162.20)
SBEtOAc	45.5 (23.98 – 67.0)[S]	58.1(28.57 – 87.66)[S]
SWEtOAc	79.1 (72.38 – 85.85)	93.7 (91.33 – 95.99)
RBH	10.8 (1.15 – 20.47)[S]	15.1 (-3. 071 – 33.16)[S]
RWH	83.7 (79.53 – 87.94)	92.8 (85.28 – 100.30)
RWEtOAc	17.7 (14.03 – 21.35)[S]	81.1 (50.64 – 111.50)
RBEtOAc	92.2 (88.65 – 95.79)	90.4 (78.16 – 102.60)
LEtOAc	78.9 (60.26 – 97.60)	94.7 (86.08 – 102.20)
RWEtOH	76.9 (65.43 – 88.30)[S]	94.0 (90.82 – 97.14)
Amphotericin B	0.00*	0.00*

SW: stem wood, SB: stem bark, RB: root bark, RW: root wood, L: leaf. H: hexane, EtOAc: ethyl acetate, EtOH: ethanol.

Amphotericin B was used as positive control. *The values for Amphotericin B were low comparing to the others, the viability was considered null. [S]Samples that are statistically significant ($p < 0.05$) when compared to DMSO in the Dunnet's test comparison of the means.

Data reported as the average of 3 independent experiments performed in duplicate.

S2 Table. ***In vitro*** **antileishmanial activity, cytotoxicity in murine peritoneal macrophages, and selectivity index of *C. suberosus* extracts**

	Promastigotes [a]IC_{50}		Amastigotes IC_{50}	Cytotoxicity [b]CC_{50}	
**Sample**	***L. amazonensis***	***L. infantum***	***L. amazonensis***	**Murine macrophages**	[c]**SI**
	**µg/mL**	**µg/mL**	**µg/mL**	**µg/mL**	
**RWH**	>100.0	>100.0	>100.0	>100.0	[d]-
**RWEtOAc**	29.9 ± 1.1	21.1 ± 2.0	26.6 (23.3-30.2)	90.8 (65.7-125.4)	3.4
**RWEtOH**	>100.0	>100.0	>100.0	>100.0	-
**RBEtOAc**	74.3 (65.8-84.0)	68.0 ± 6.6	58.6 (50.8-67.7)	>100.0	-
**LEtOAc**	>100.0	>100.0	>100.0	>100.0	-
**SWH**	>100.0	>100.0	>100.0	37.1 (30.7-45.0)	-
**SBEtOAc**	>100.0	>100.0	>100.0	>100.0	-
**SWEtOAc**	>100.0	>100.0	>100.0	>100.0	-
**Miltefosine**	9.0 ± 0.5	2.6 ± 0.3	5.2 (4.8-5.5)	53.6 (49.7-57.6)	10.4

RW: root wood, RB: root bark, L: leaf, SW: stem wood, SB: stem bark. H: hexane, EtOAc: ethyl acetate, EtOH: ethanol.

[a]IC_{50}: Concentration required to inhibit 50% of parasite growth after 72 h exposure.

[b]CC_{50}: Cytotoxic concentration to reduce cell viability by 50% after 72 h exposure.

[c]SI: Selectivity Index.

[d]-Not determined.

Miltefosine was used as reference compound. Data reported as the average of 3 independent experiments performed in duplicate.

S3 Table. ^{1}H (600 MHz) and ^{13}C (75 MHz) NMR data assignments for hemileiocarpin (1) and leiocarpin (2). Chemical shifts (ppm) and coupling constants (J, Hz, in parenthesis)

δC Type and position	hemileiocarpin (**1**)		leiocarpin (**2**)	
	**δC**	**δH**	**δC**	**δH**
**CH**				
1	130.8	7.27 (1; d)	130.7	7.24 d (7.0 Hz)
2	110.5	6.53 dd (8.3 Hz; 0.6 Hz)	110.5	6.52 dd (8.5 Hz; 0.7 Hz)
6a	39.5	3.52 m	40.2	3.46 m
8	124.7	7.13 dd (8.7 Hz; 0.5 Hz)		
7	106.3	6.45 m/ 6.46 m	104.7	6.72 bs
10	96.9	6.45 m/ 6.46 m	93.8	6.43 bs
11a	78.9	5.49 bd (11.0 Hz)	78.8	5.46 bd (7.0 Hz)
1'	116.5	6.63 dd (10.0 Hz; 0.6 Hz)	116.5	6.62 dd (10.0 Hz; 0.6 Hz)
2'	129.2	5.57 d (10.0 Hz)	129.2	5.57 d (10.0 Hz)
**CH2**				
			101.3	5.91 dd (14.5 Hz; 1.4 Hz)
6	66.7	3.63 m (11.0 Hz) 4.28 ddd (11.0 Hz; 5.1 Hz; 0.6 Hz)	66.6	4.26 ddd (11 Hz; 5.0 Hz; 0.6 Hz) 3.65 m
**CH3**				
5'	27.8/ 27.9	1.42 bs	27.8	1.42 s
6'	27.8/ 27.9	1.42 bs	27.9	1.42 s
**OCH3**	55.5	3.77 s		
C				
4a	154.1		154.1	
1a	110.2		110.2	
3	151.2		151.2	
7a	119.2		117.9	
9	161.1		148.1	
10a	160.8		154.3	
4	112.2		112.2	
3'	76.1		76.1	
8			141.7	

(A)

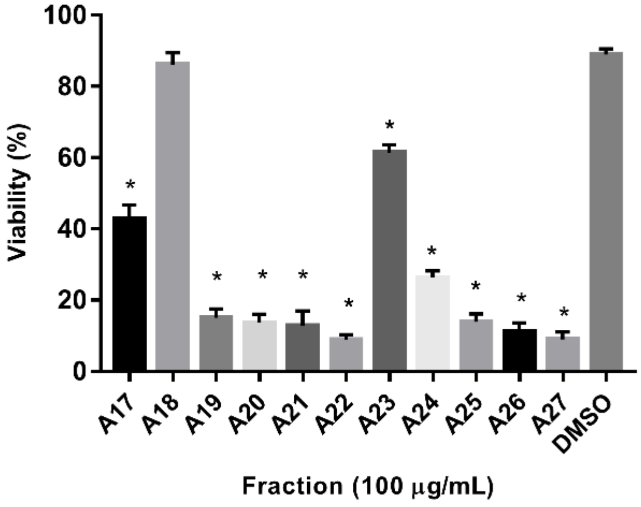

(B)

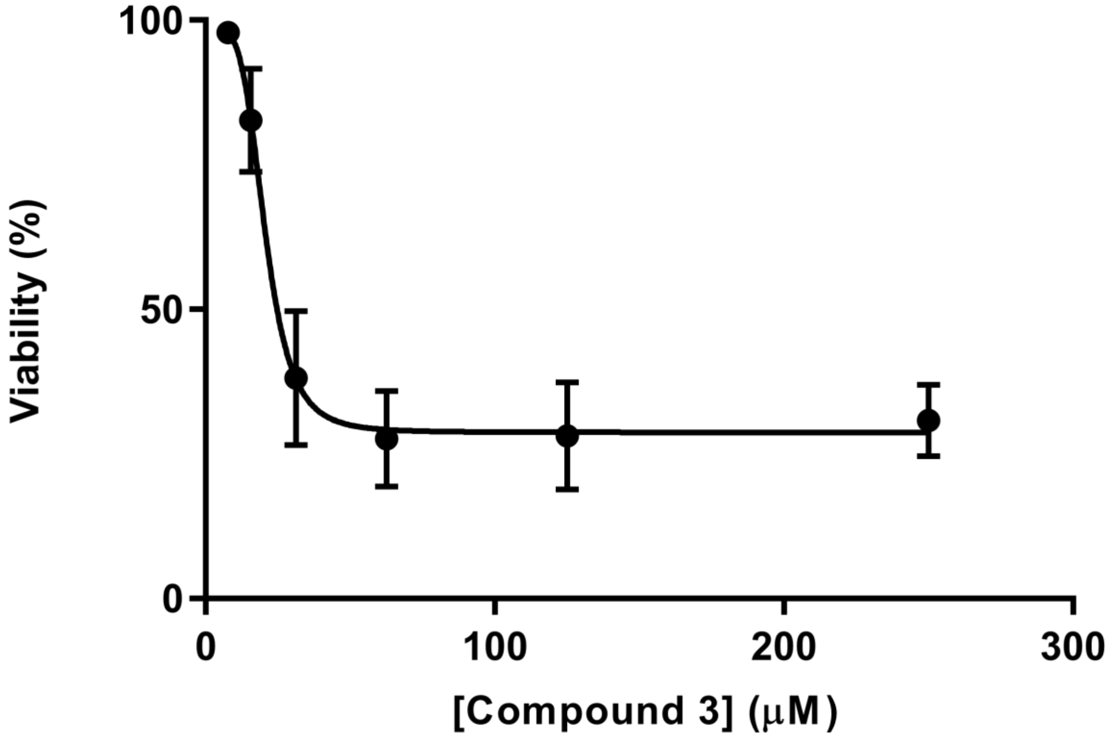

S1 Fig. Activity of silica column fractions of *C. suberosus* root bark hexane (RBH) extract. (A) Inhibitory effect of fractions A17-A27 (100 μg/mL) against *L. amazonensis* promastigotes. *Statistically significant ($p < 0.05$) when compared to the DMSO negative control using the Dunnett's test. **(B)** Dose-response curve of connarin (**3**) against *L. amazonensis* promastigotes after 24 h exposure

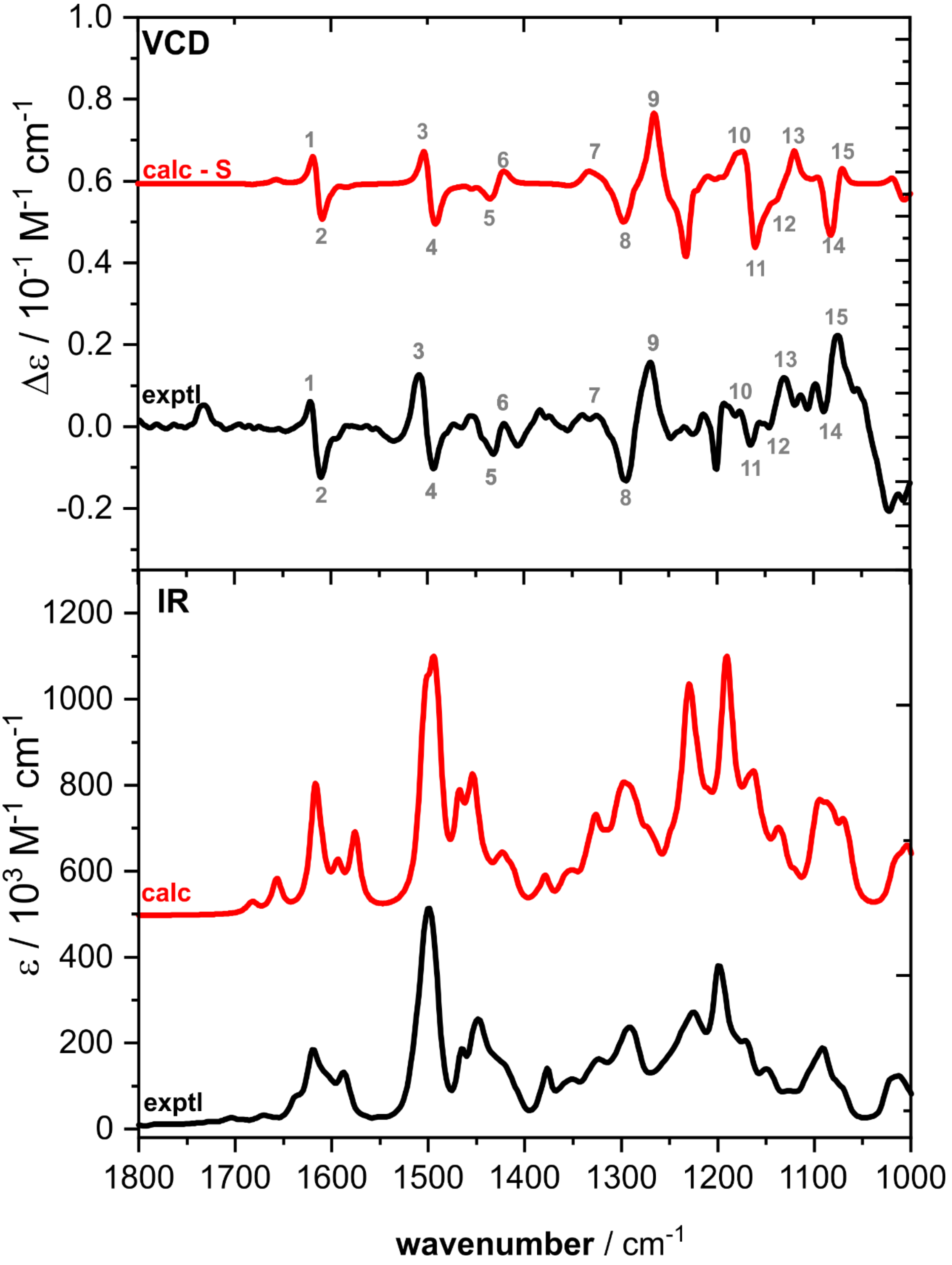

S2 Fig. Vibrational circular dichroism and infrared experimental (exptl) spectra of connarin (3) in comparison with calculated (calc) spectra for (S) configuration

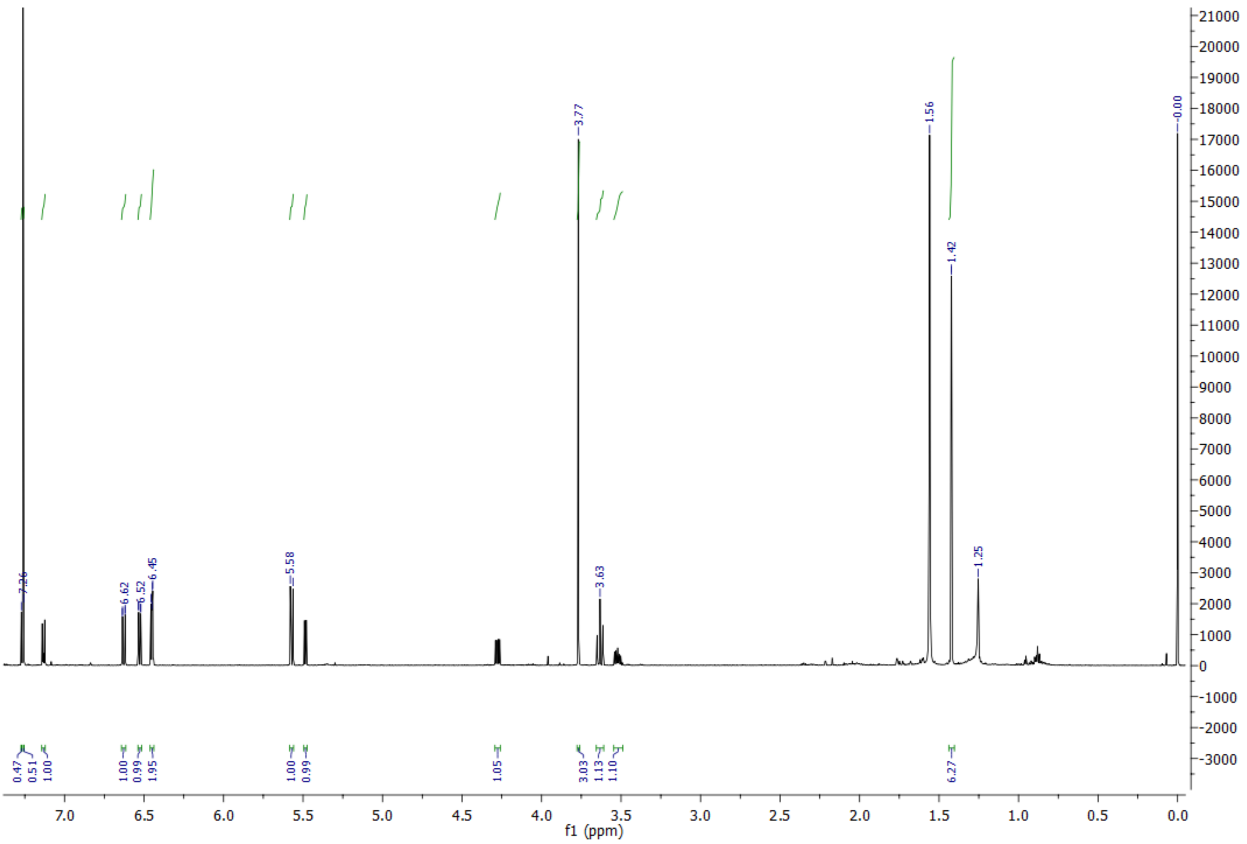

S3 Fig. ^{1}H NMR spectrum (600 MHz, $CDCl_3$) of hemileiocarpin (1)

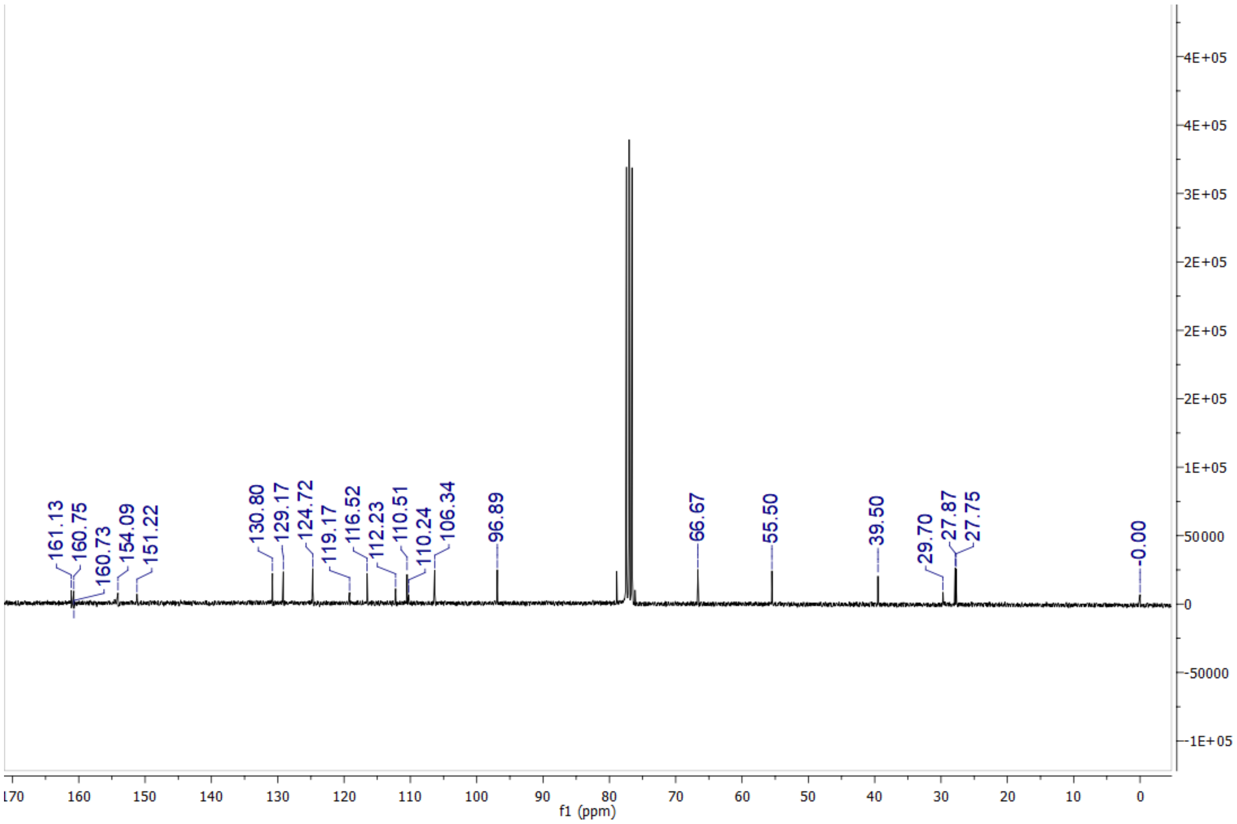

S4 Fig. ^{13}C NMR spectrum (75 MHz, $CDCl_3$) of hemileiocarpin (1)

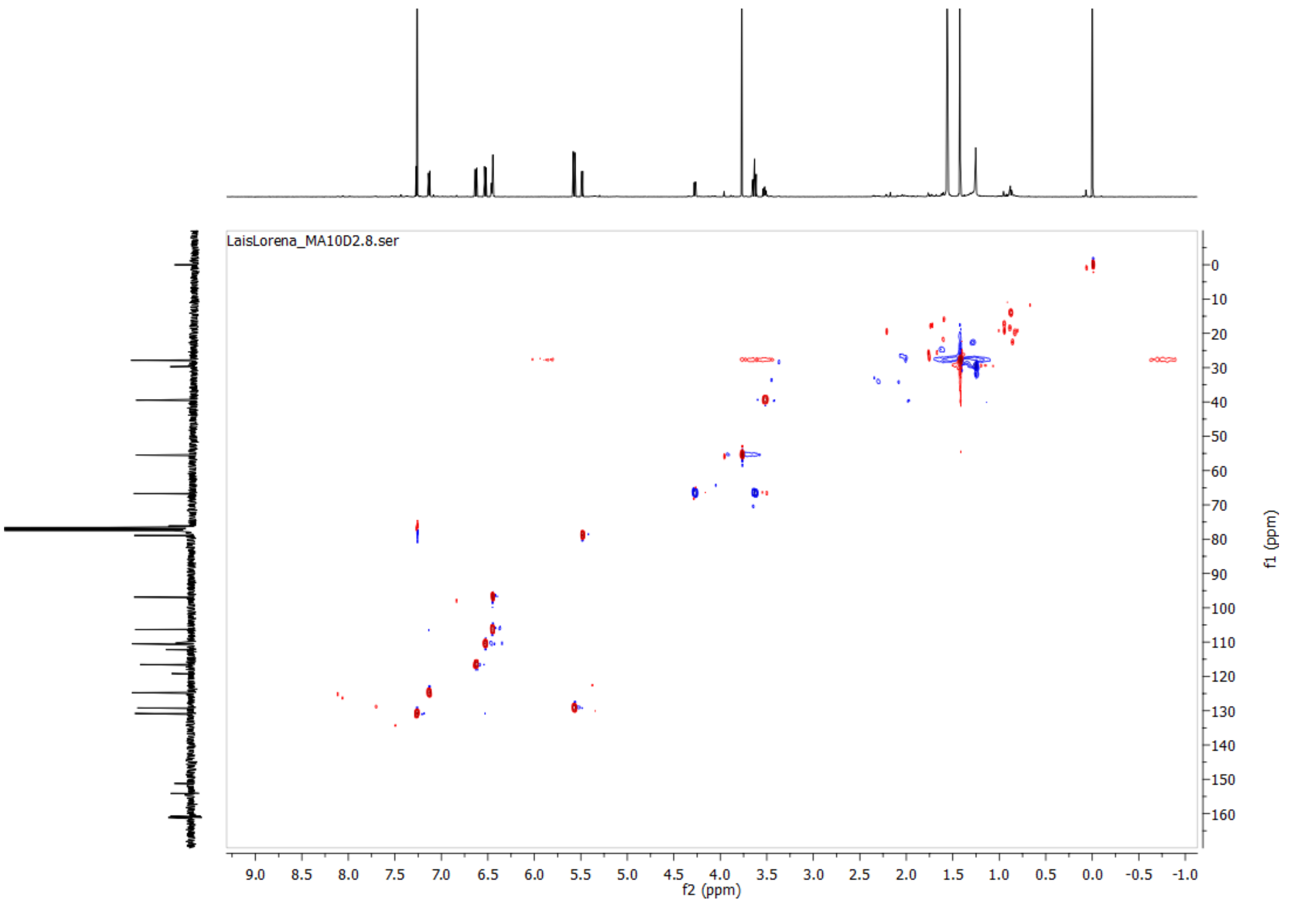

S5 Fig. Edited HSQC spectrum ($CDCl_3$) of hemileiocarpin (1)

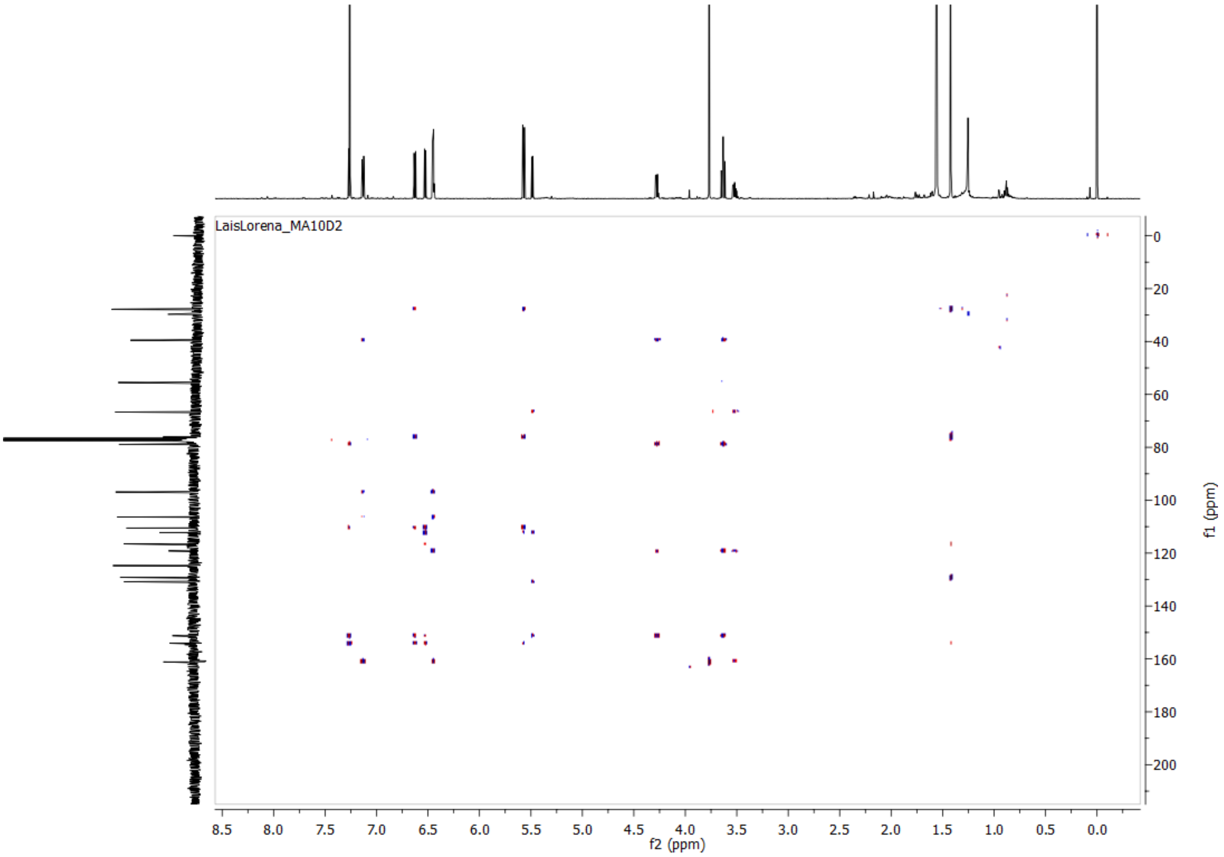

S6 Fig. HMBC spectrum ($CDCl_3$) of hemileiocarpin (1)

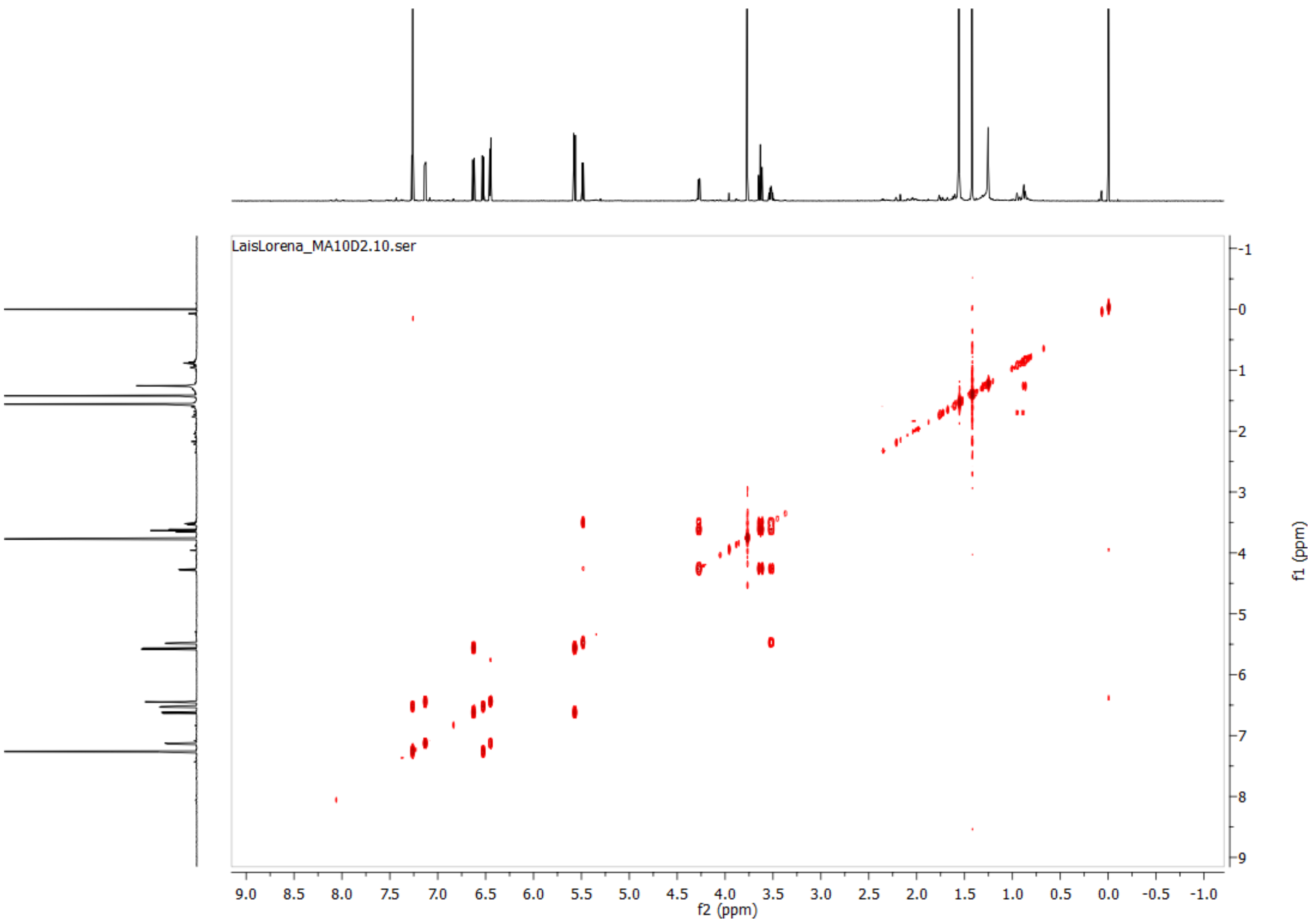

S7 Fig. COSY spectrum ($CDCl_3$) of hemileiocarpin (1)

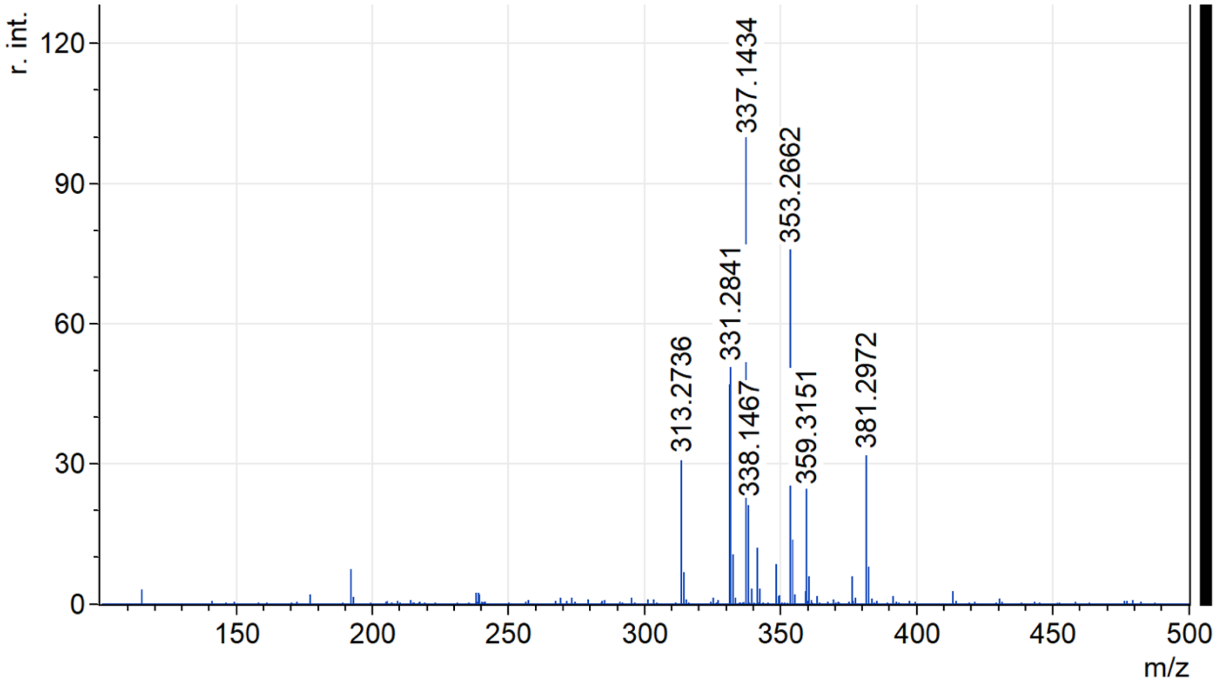

S8 Fig. HRESIMS spectrum of hemileiocarpin (1)

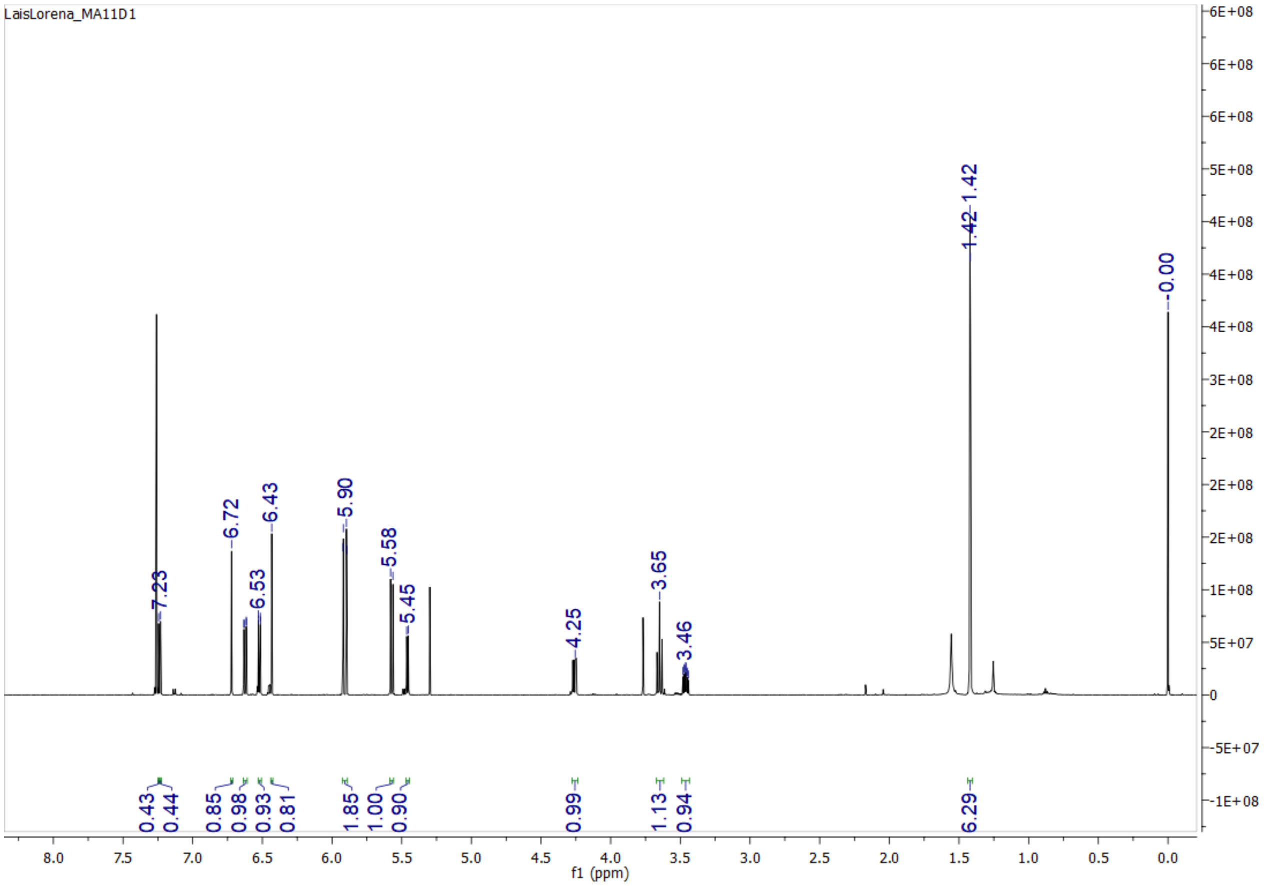

S9 Fig. **^{1}H NMR spectrum (600 MHz $CDCl_3$) of leiocarpin (2)**

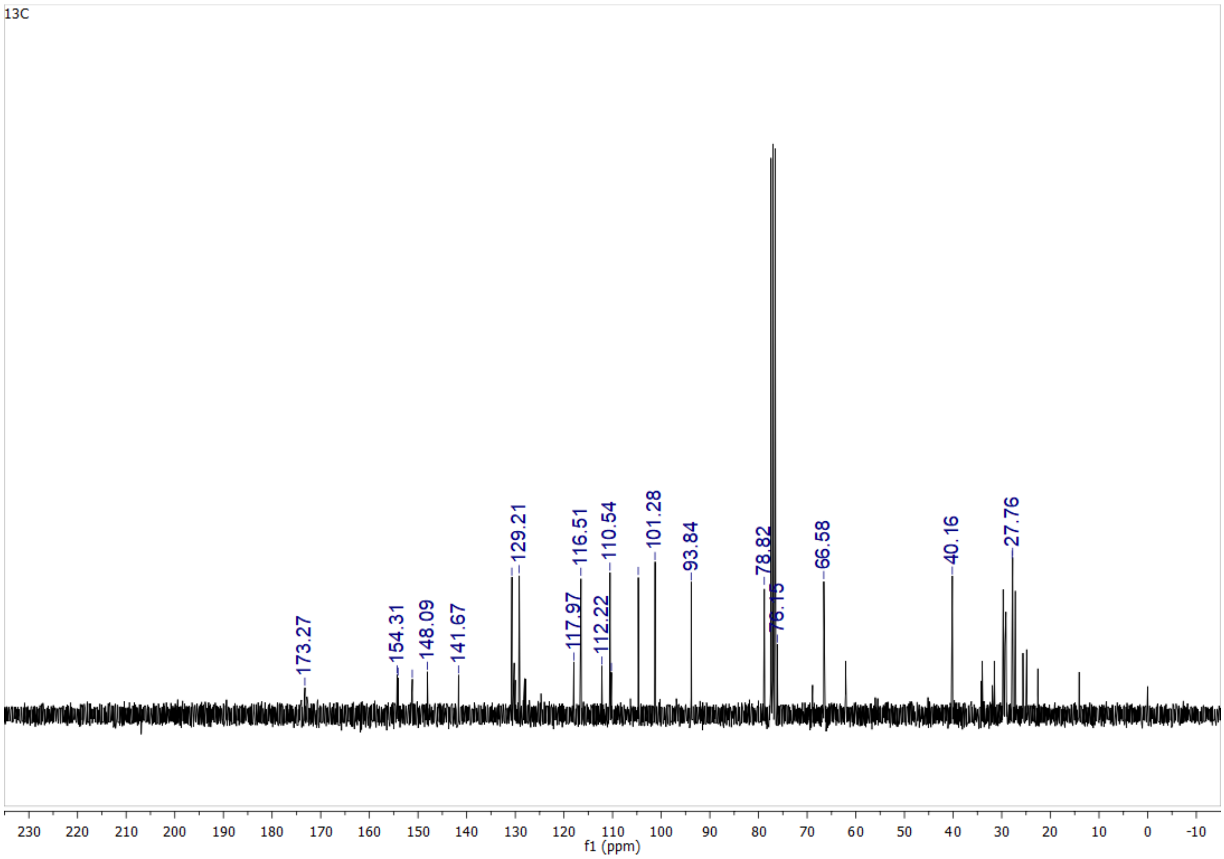

S10 Fig. **^{13}C NMR spectrum (75 MHz, $CDCl_3$) of leiocarpin (2)**

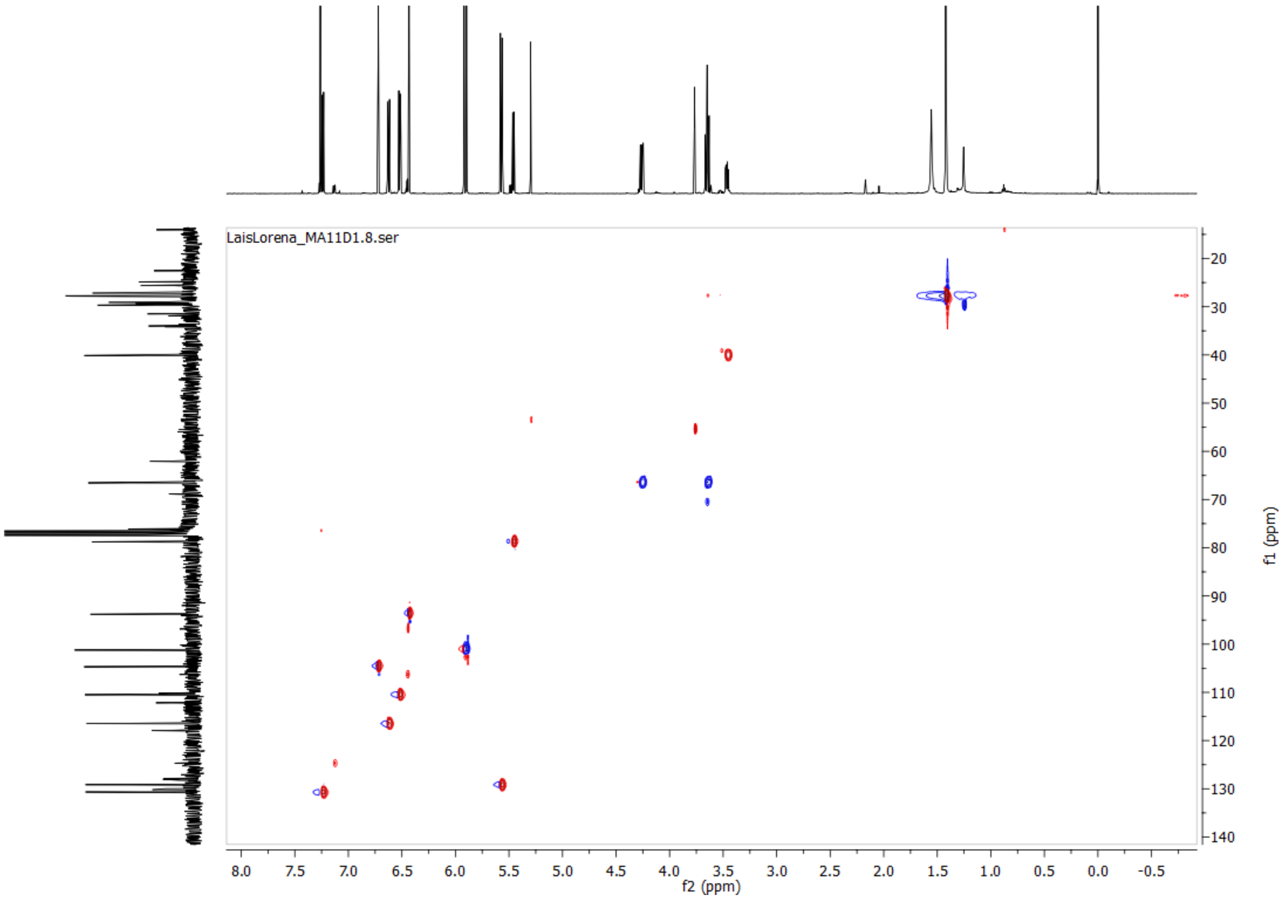

S11 Fig. Edited HSQC spectrum ($CDCl_3$) of leiocarpin (2)

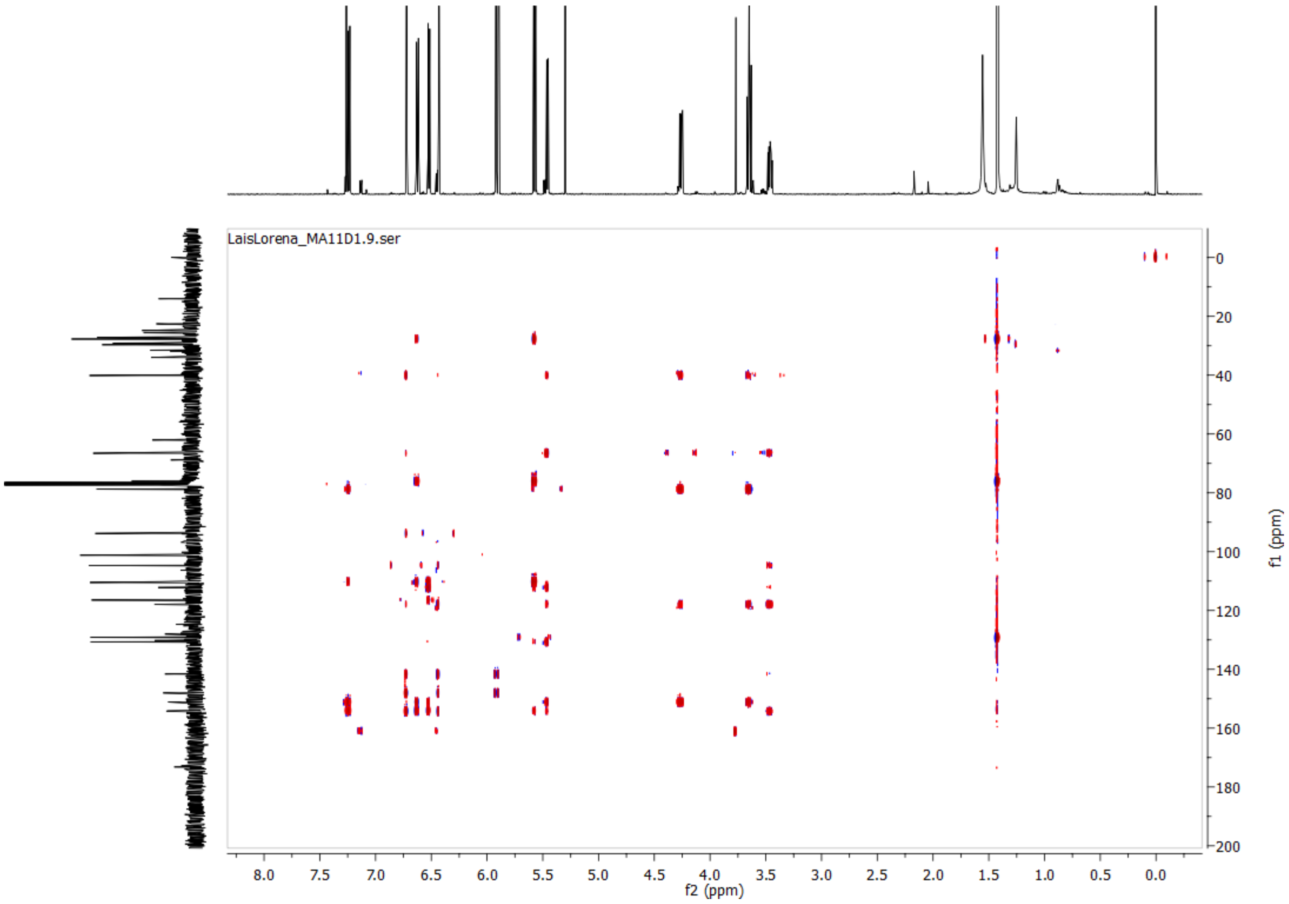

S12 Fig. HMBC spectrum ($CDCl_3$) of leiocarpin (2)

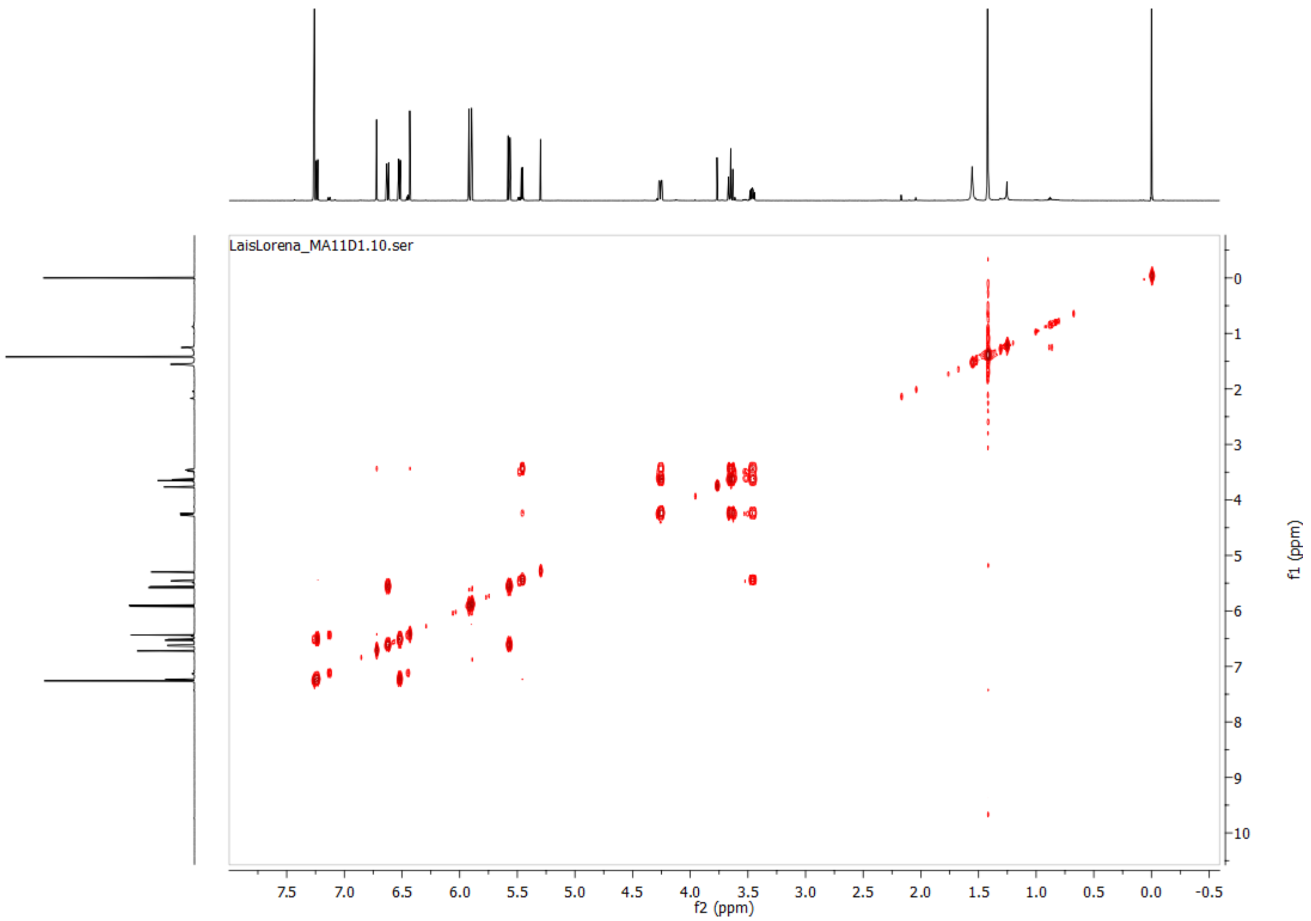

S13 Fig. COSY spectrum ($CDCl_3$) of leiocarpin (2)

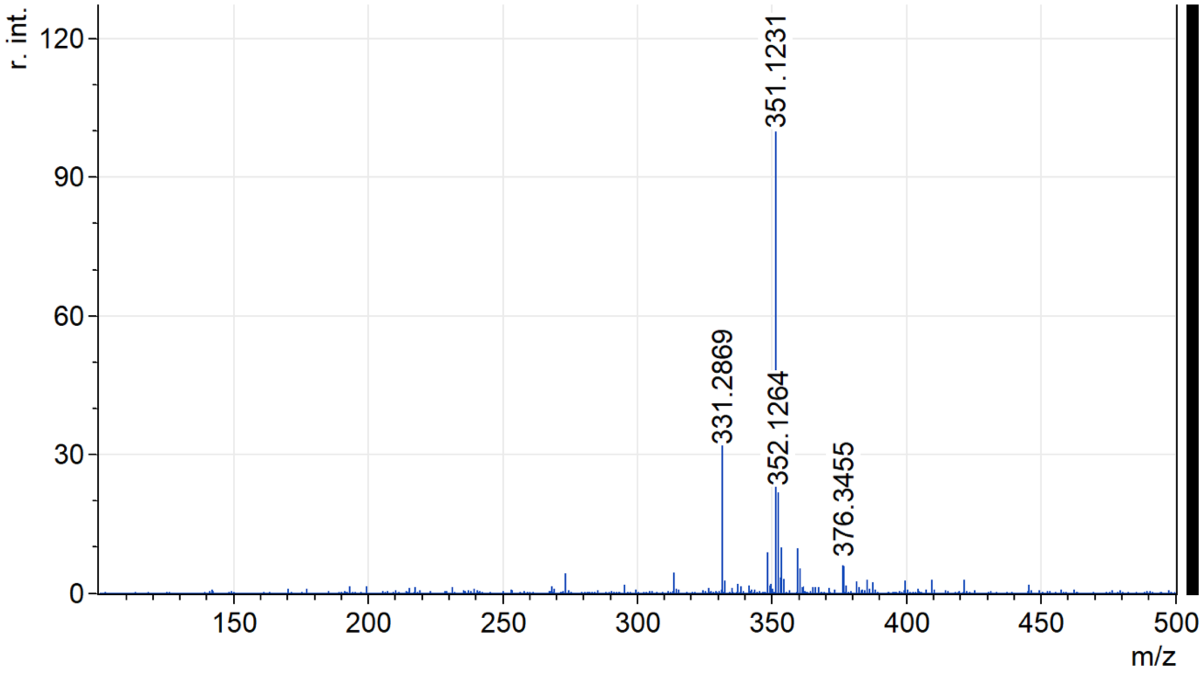

S14 Fig. HRESIMS spectrum of leiocarpin (2)

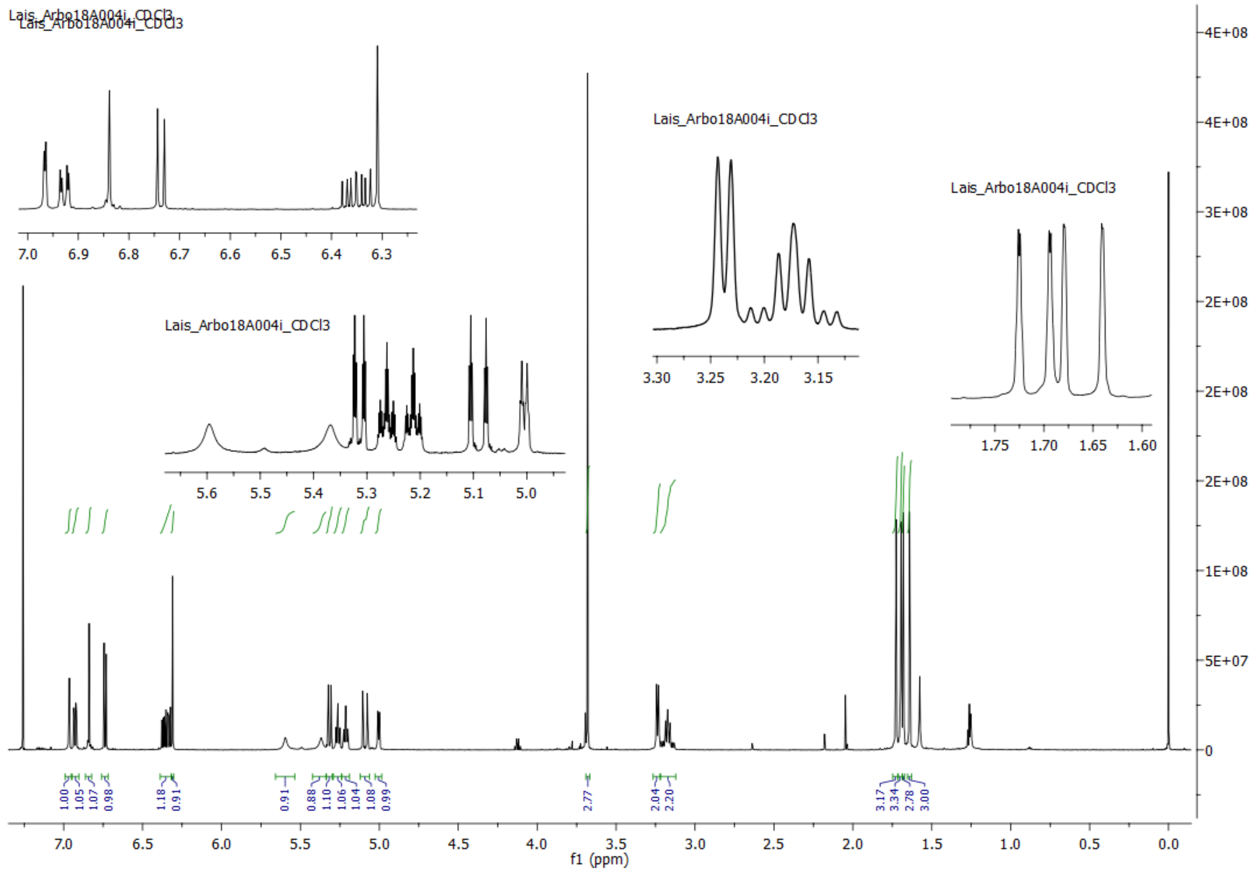

S15 Fig. ^{1}H NMR spectrum (600 MHz $CDCl_3$) of connarin (3)

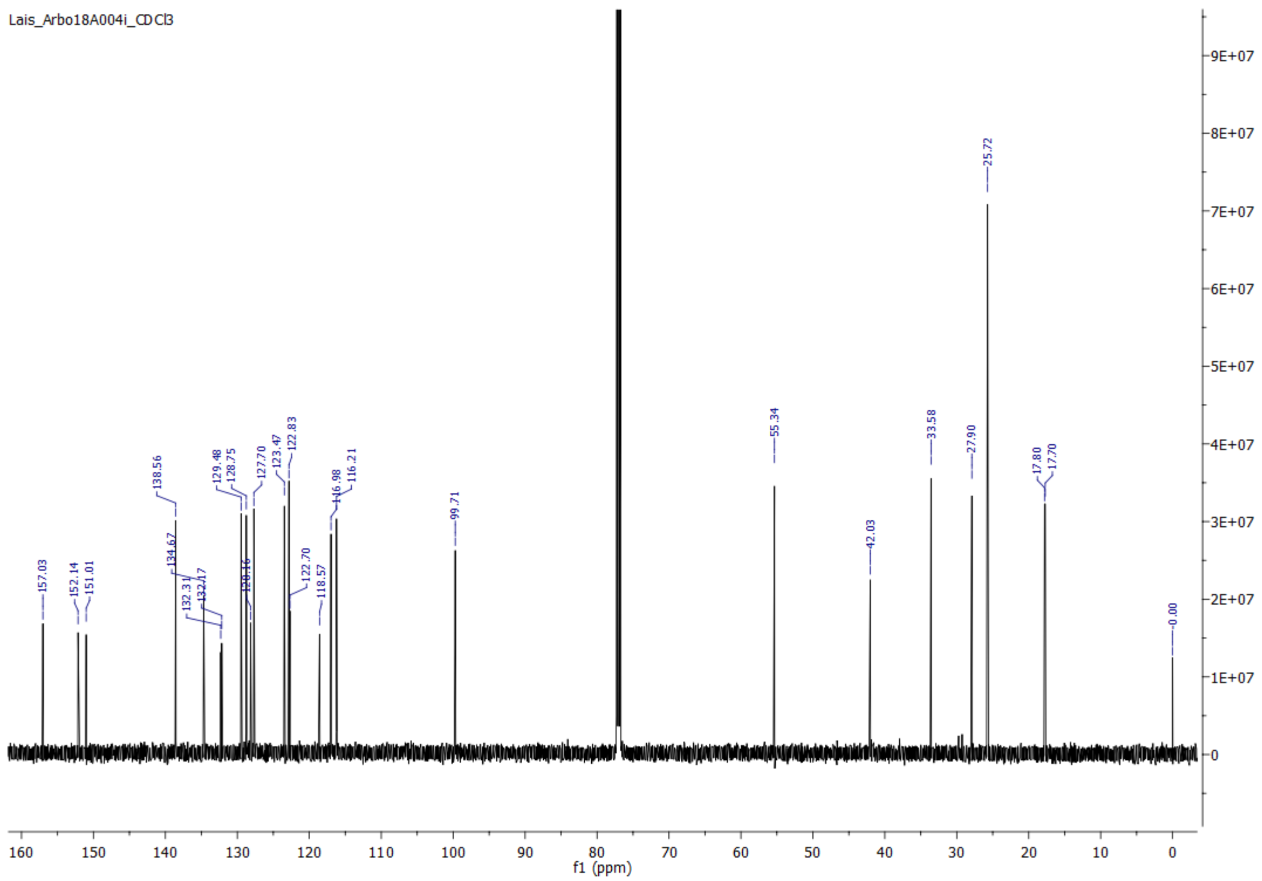

S16 Fig. ^{13}C NMR spectrum (150 MHz, $CDCl_3$) of connarin (3)

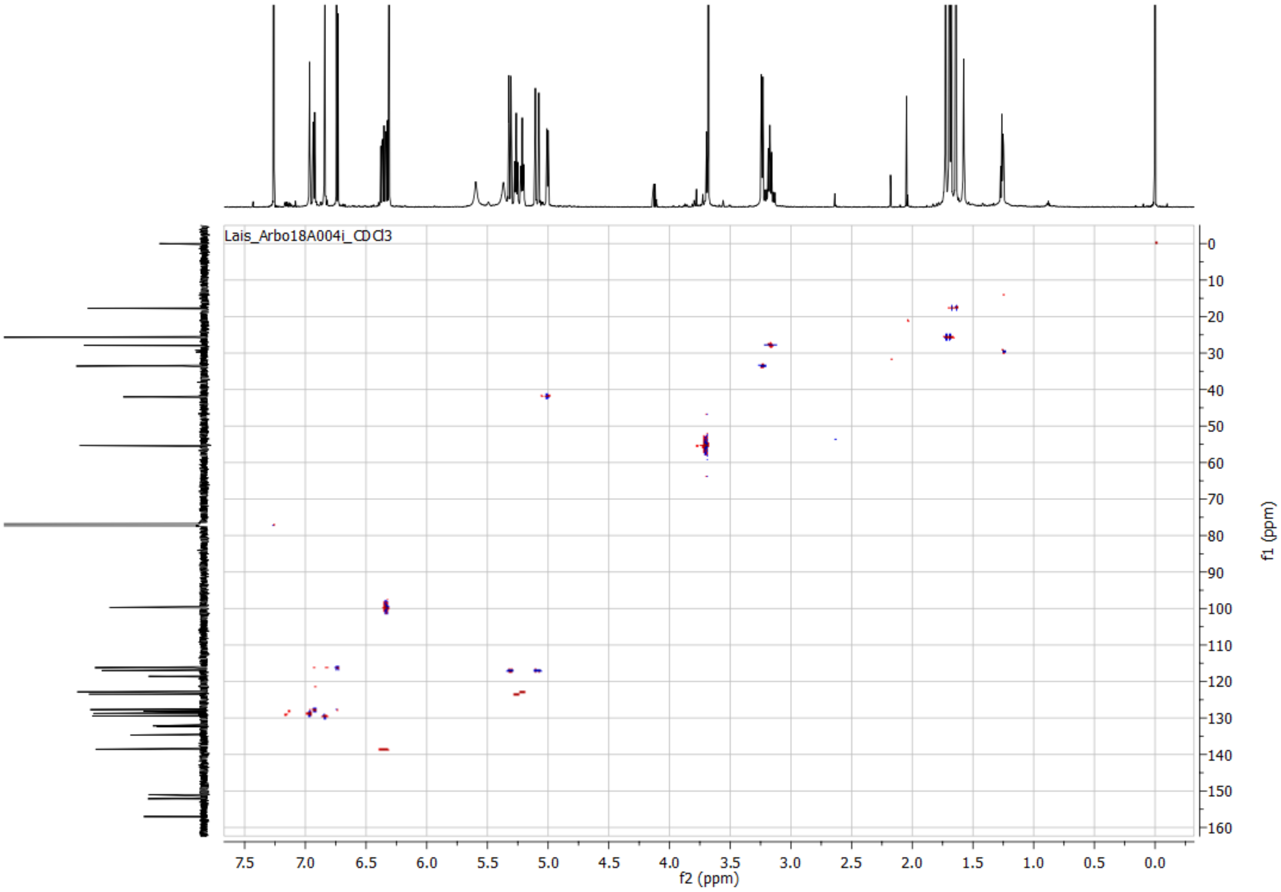

S17 Fig. Edited HSQC spectrum ($CDCl_3$) of connarin (3)

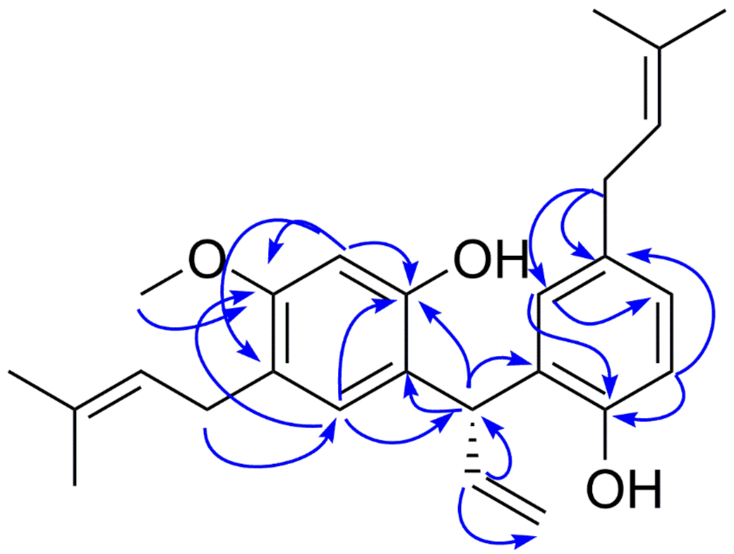

S18 Fig. HMBC selected correlations of connarin (3)

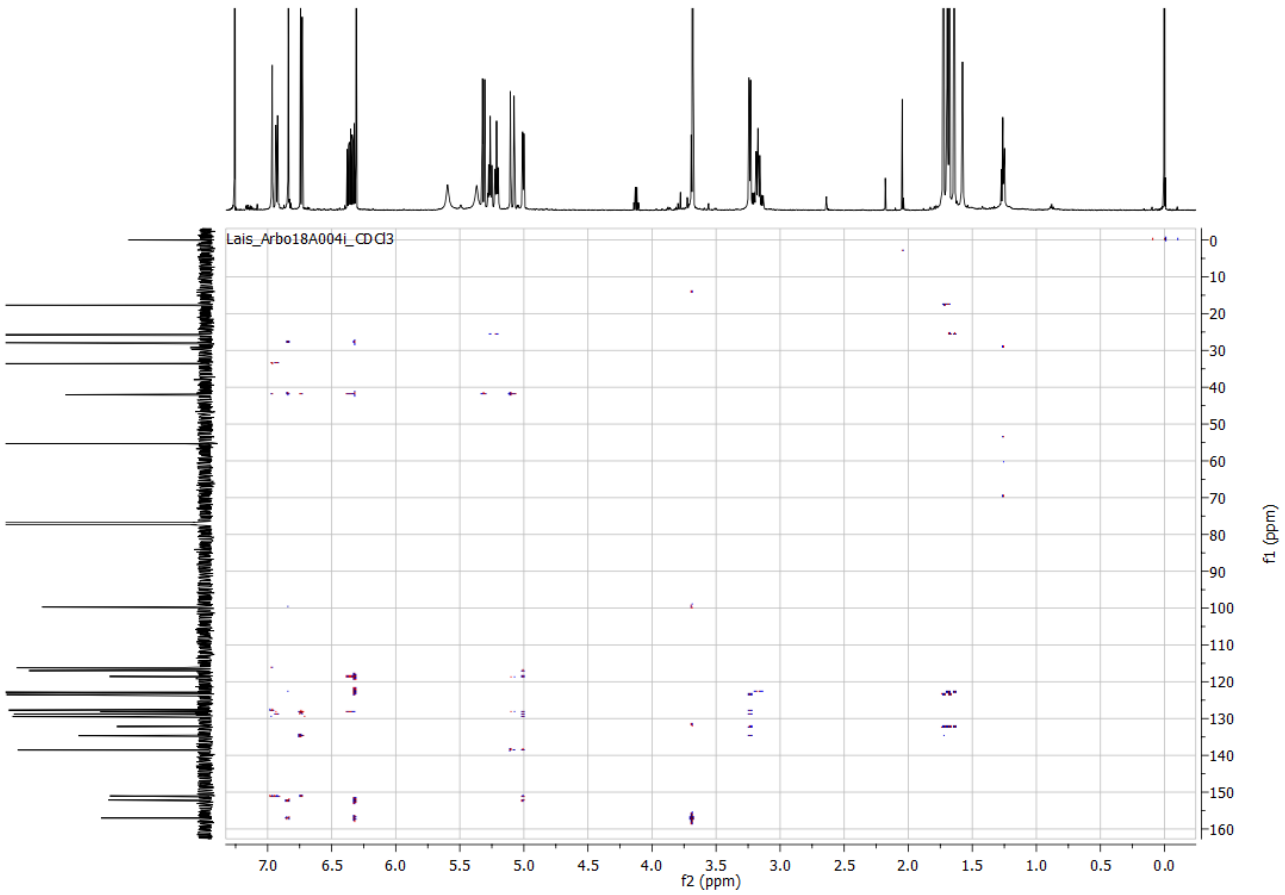

S19 Fig. HMBC spectrum ($CDCl_3$) of connarin (3)

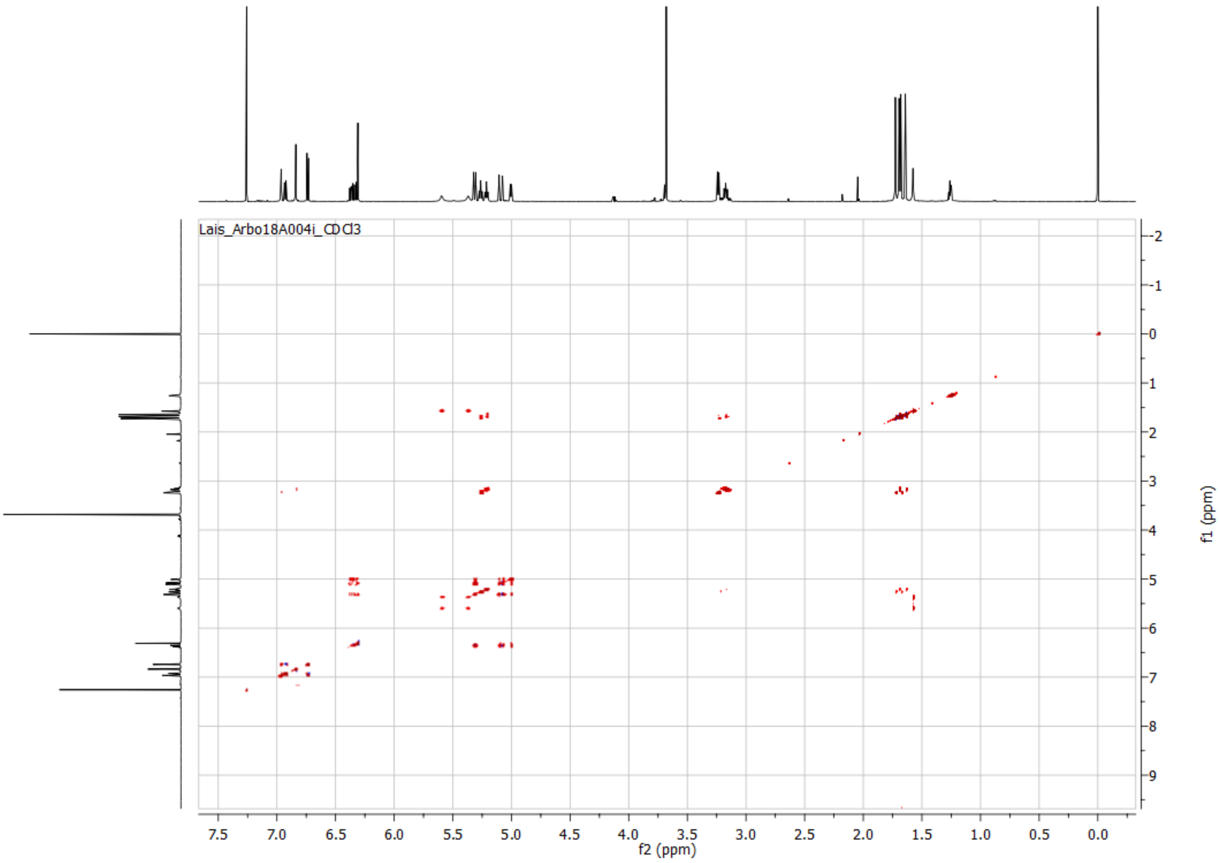

S20 Fig. COSY spectrum ($CDCl_3$) of connarin (3)

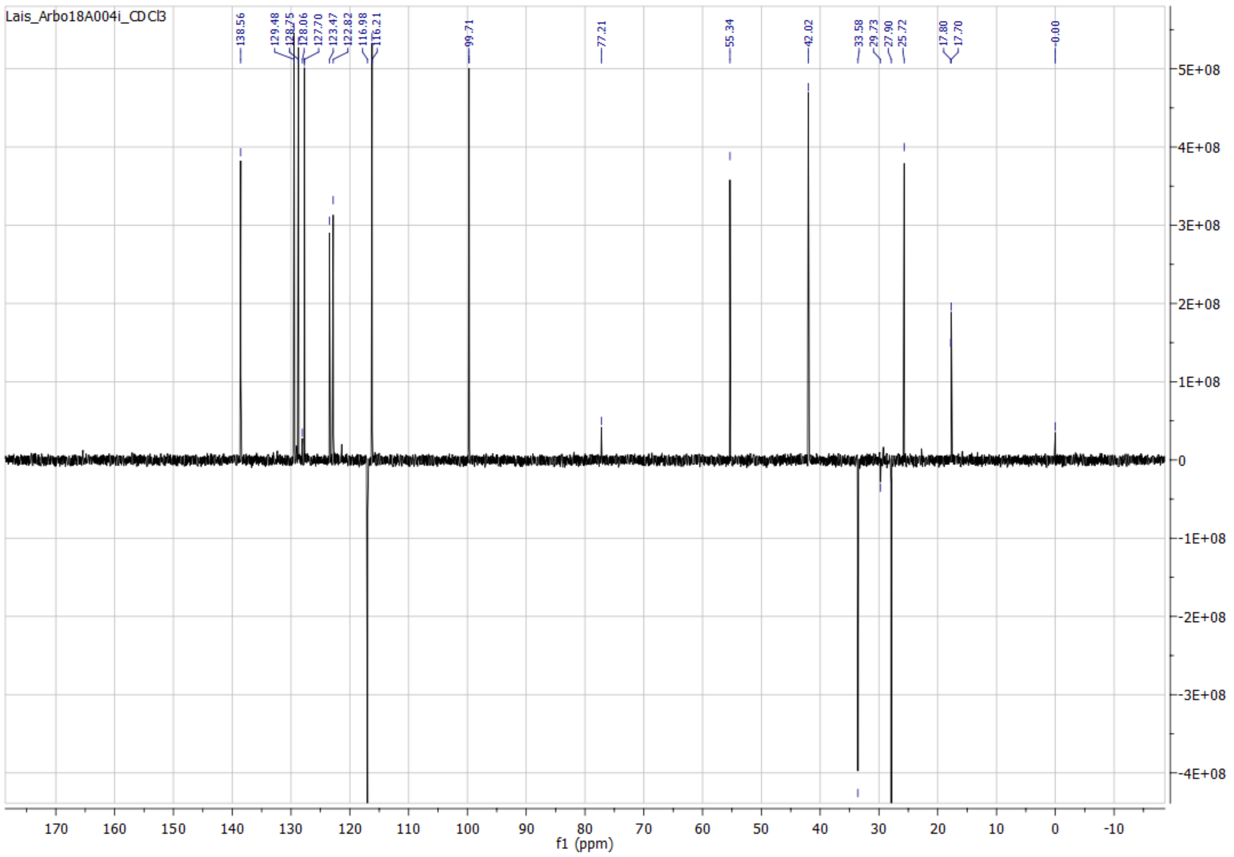

S21 Fig. DEPT spectrum (150 MHz, $CDCl_3$) of connarin (3)

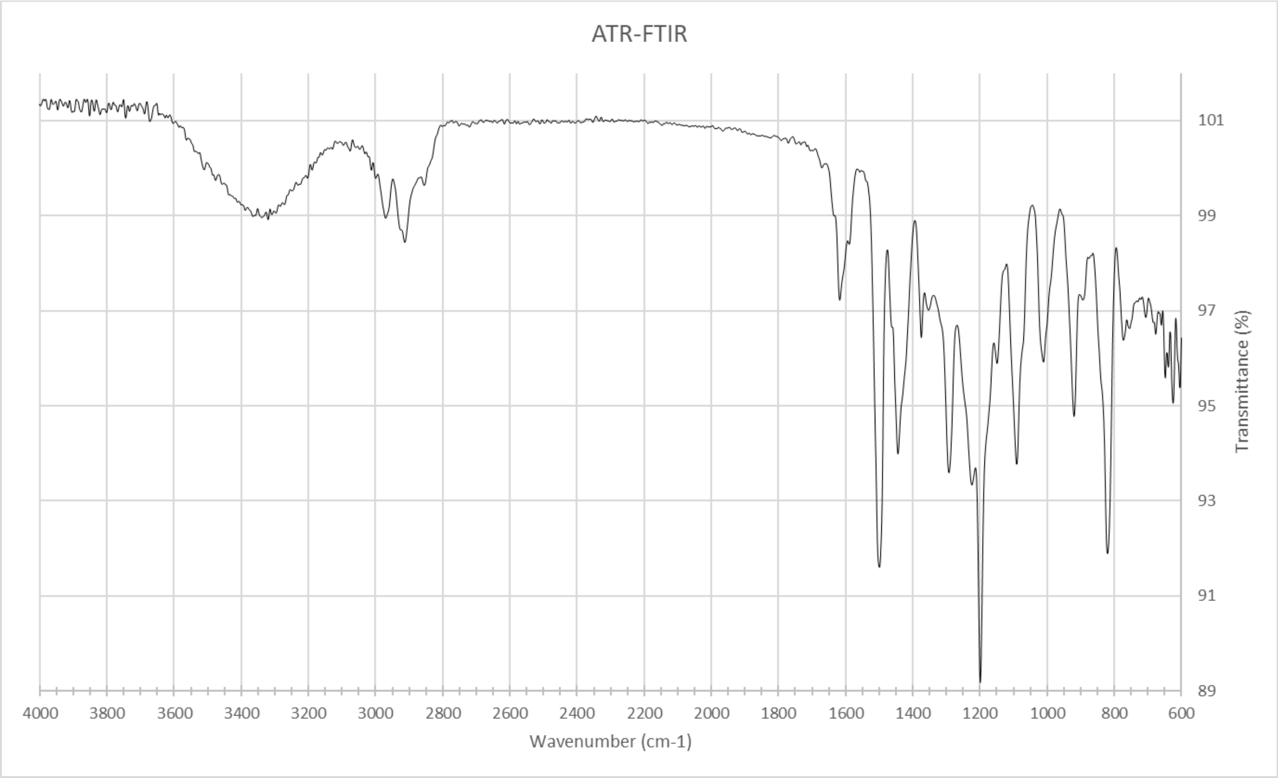

S22 Fig. IR spectrum of connarin (3)

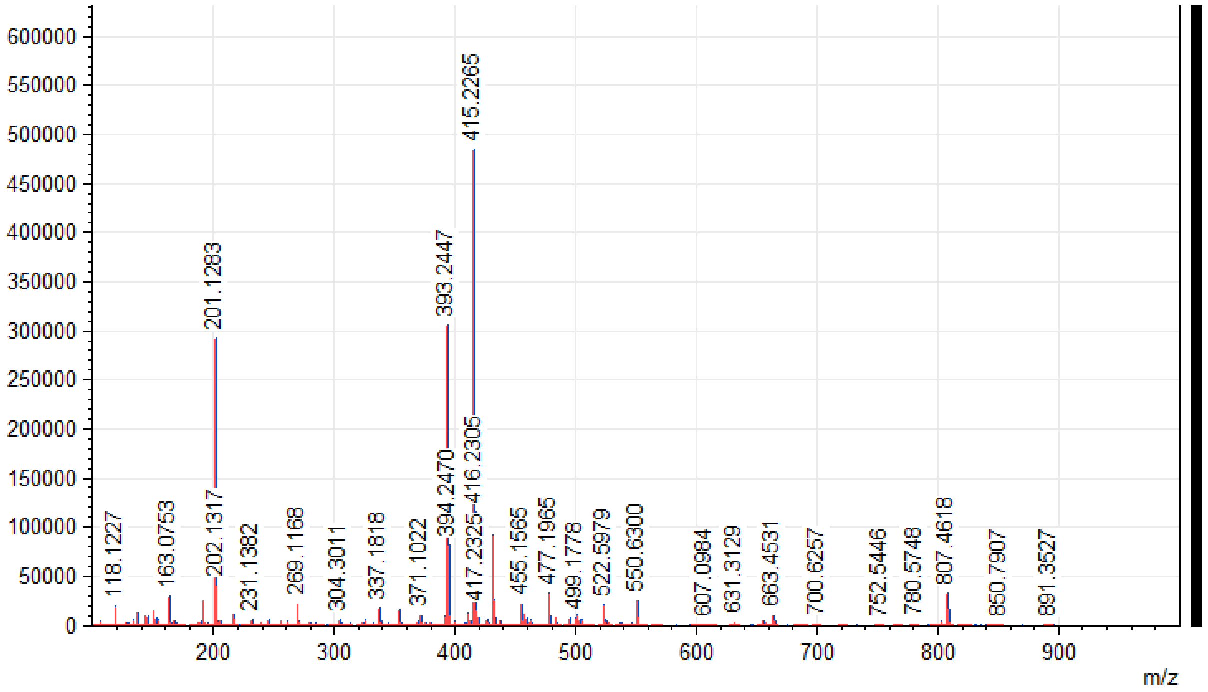

S23 Fig. HRESIMS spectrum of connarin (3)

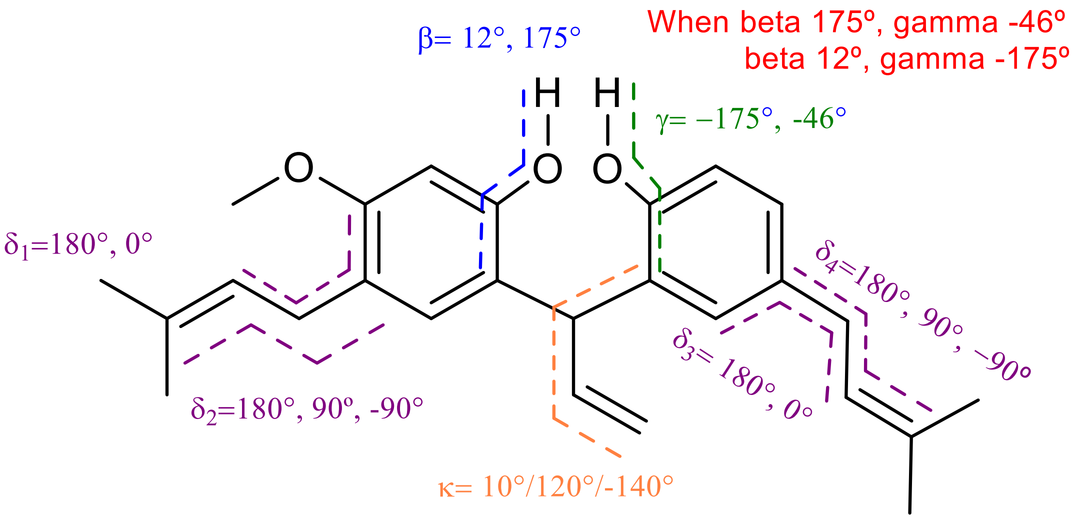

S24 Fig. Systematic conformational search performed for connarin (3)

S4 Table. Starting angles of selected conformers for connarin (3)

conformer	β	γ	κ	δ1	δ2	δ3	δ4
g1a_c27	12	-175	10	0	90	180	-90
g1a_c21	12	-175	10	0	180	180	-90
g1a_c19	12	-175	10	0	180	180	180
g1a_c31	12	-175	10	0	-90	180	180
g2a_c15	12	-175	120	180	-90	180	-90

S1 File. Cartesian coordinates of selected conformers for connarin (3)

Conformer g1a_c27

C	-2.77302	-2.7694	-0.14177
C	-1.57425	-2.73049	0.56523
C	-0.94201	-1.51972	0.8487
C	-1.54128	-0.35296	0.36613
C	-2.7363	-0.35874	-0.34816
C	-3.35122	-1.59042	-0.58676
H	-3.2465	-3.72391	-0.34695
H	-1.05021	0.5948	0.55537
C	0.38433	-1.49281	1.60415
C	0.4029	-0.62052	2.84365
H	1.38963	-0.5284	3.2908
C	-0.62533	-0.03995	3.45076
H	-1.63609	-0.09324	3.06532
H	-0.47955	0.5124	4.37164
O	-0.95201	-3.89346	0.98796
H	-1.45064	-4.66217	0.68357
C	1.55724	-1.19782	0.66832
C	2.08489	0.09171	0.53542
C	2.138	-2.20986	-0.10107
C	3.14874	0.40787	-0.29712
H	1.64368	0.89024	1.12034
C	3.21421	-1.93321	-0.94658
C	3.7145	-0.64119	-1.04575
H	3.62938	-2.75026	-1.5187
O	1.72742	-3.51723	-0.08205
O	4.75733	-0.30005	-1.86101
C	5.38491	-1.32264	-2.62806
H	6.18272	-0.83221	-3.18136
H	5.81106	-2.09653	-1.98397
H	4.68242	-1.77875	-3.33082
H	0.87429	-3.63196	0.36893
H	0.54848	-2.50092	1.99754
C	3.70091	1.81761	-0.43596
H	2.88312	2.46567	-0.77046
H	3.9704	2.1938	0.55465
C	4.84526	1.96234	-1.39899
H	5.82903	1.80825	-0.95897
C	4.79901	2.2312	-2.70768
C	6.06313	2.29548	-3.52716
H	6.95033	2.105	-2.92147
H	6.03946	1.56112	-4.33995
H	6.17383	3.27767	-3.99963
C	3.53873	2.48896	-3.49131
H	2.63908	2.48314	-2.87826
H	3.59733	3.45969	-3.99512
H	3.41638	1.73843	-4.27993
H	-4.28802	-1.63038	-1.13066
C	-3.35316	0.93133	-0.86171
H	-3.39171	0.9031	-1.95278
H	-2.6823	1.75742	-0.60238
C	-4.71794	1.20717	-0.28531
H	-4.7044	1.78658	0.63601
C	-5.9116	0.83398	-0.75822
C	-7.17835	1.2179	-0.03584
H	-7.74696	0.32781	0.25513
H	-6.97297	1.80142	0.8627
H	-7.83504	1.80744	-0.68498
C	-6.13382	0.02876	-2.01154
H	-6.7289	0.6005	-2.73187
H	-5.21096	-0.27205	-2.50464
H	-6.70802	-0.87629	-1.78612

Conformer g1a_c21

C	-2.77302	-2.7694	-0.14177
C	-1.57425	-2.73049	0.56523
C	-0.94201	-1.51972	0.8487
C	-1.54128	-0.35296	0.36613
C	-2.7363	-0.35874	-0.34816
C	-3.35122	-1.59042	-0.58676
H	-3.2465	-3.72391	-0.34695
H	-1.05021	0.5948	0.55537
C	0.38433	-1.49281	1.60415
C	0.4029	-0.62052	2.84365
H	1.38963	-0.5284	3.2908
C	-0.62533	-0.03995	3.45076
H	-1.63609	-0.09324	3.06532
H	-0.47955	0.5124	4.37164
O	-0.95201	-3.89346	0.98796
H	-1.45064	-4.66217	0.68357
C	1.55724	-1.19782	0.66832
C	2.08489	0.09171	0.53542
C	2.138	-2.20986	-0.10107
C	3.14874	0.40787	-0.29712
H	1.64368	0.89024	1.12034
C	3.21421	-1.93321	-0.94658
C	3.7145	-0.64119	-1.04575
H	3.62938	-2.75026	-1.5187
O	1.72742	-3.51723	-0.08205
O	4.75733	-0.30005	-1.86101
C	5.38491	-1.32264	-2.62806
H	6.18272	-0.83221	-3.18136
H	5.81106	-2.09653	-1.98397
H	4.68242	-1.77875	-3.33082
H	0.87429	-3.63196	0.36893
H	0.54848	-2.50092	1.99754
C	3.70091	1.81761	-0.43596
H	2.88312	2.46567	-0.77046
H	3.9704	2.1938	0.55465
C	4.84526	1.96234	-1.39899
H	4.5932	1.74909	-2.43637
C	6.10344	2.33402	-1.14221
C	7.11152	2.46554	-2.25583
H	6.67821	2.22256	-3.22715
H	7.50948	3.48525	-2.304
H	7.96939	1.80539	-2.08725
C	6.64585	2.65521	0.22581
H	5.91916	2.51294	1.02388
H	7.51578	2.02723	0.44584
H	6.99542	3.69267	0.26666
H	-4.28802	-1.63038	-1.13066
C	-3.35316	0.93133	-0.86171
H	-3.39171	0.9031	-1.95278
H	-2.6823	1.75742	-0.60238
C	-4.71794	1.20717	-0.28531
H	-4.7044	1.78658	0.63601
C	-5.9116	0.83398	-0.75822
C	-7.17835	1.2179	-0.03584
H	-7.74696	0.32781	0.25513
H	-6.97297	1.80142	0.8627
H	-7.83504	1.80744	-0.68498
C	-6.13382	0.02876	-2.01154
H	-6.7289	0.6005	-2.73187
H	-5.21096	-0.27205	-2.50464
H	-6.70802	-0.87629	-1.78612

Conformer g1a_c19

C	-2.77302	-2.7694	-0.14177
C	-1.57425	-2.73049	0.56523
C	-0.94201	-1.51972	0.8487
C	-1.54128	-0.35296	0.36613
C	-2.7363	-0.35874	-0.34816
C	-3.35122	-1.59042	-0.58676
H	-3.2465	-3.72391	-0.34695
H	-1.05021	0.5948	0.55537
C	0.38433	-1.49281	1.60415
C	0.4029	-0.62052	2.84365
H	1.38963	-0.5284	3.2908
C	-0.62533	-0.03995	3.45076
H	-1.63609	-0.09324	3.06532
H	-0.47955	0.5124	4.37164
O	-0.95201	-3.89346	0.98796
H	-1.45064	-4.66217	0.68357
C	1.55724	-1.19782	0.66832
C	2.08489	0.09171	0.53542
C	2.138	-2.20986	-0.10107
C	3.14874	0.40787	-0.29712
H	1.64368	0.89024	1.12034
C	3.21421	-1.93321	-0.94658
C	3.7145	-0.64119	-1.04575
H	3.62938	-2.75026	-1.5187
O	1.72742	-3.51723	-0.08205
O	4.75733	-0.30005	-1.86101
C	5.38491	-1.32264	-2.62806
H	6.18272	-0.83221	-3.18136
H	5.81106	-2.09653	-1.98397
H	4.68242	-1.77875	-3.33082
H	0.87429	-3.63196	0.36893
H	0.54848	-2.50092	1.99754
C	3.70091	1.81761	-0.43596
H	2.88312	2.46567	-0.77046
H	3.9704	2.1938	0.55465
C	4.84526	1.96234	-1.39899
H	4.5932	1.74909	-2.43637
C	6.10344	2.33402	-1.14221
C	7.11152	2.46554	-2.25583
H	6.67821	2.22256	-3.22715
H	7.50948	3.48525	-2.304
H	7.96939	1.80539	-2.08725
C	6.64585	2.65521	0.22581
H	5.91916	2.51294	1.02388
H	7.51578	2.02723	0.44584
H	6.99542	3.69267	0.26666
H	-4.28802	-1.63038	-1.13066
C	-3.35316	0.93133	-0.86171
H	-3.39171	0.9031	-1.95278
H	-2.6823	1.75742	-0.60238
C	-4.71794	1.20717	-0.28531
H	-4.74251	1.25122	0.80198
C	-5.87159	1.39836	-0.93358
C	-7.14419	1.67248	-0.17252
H	-7.90264	0.91349	-0.39376
H	-6.97818	1.68604	0.90558
H	-7.57475	2.63635	-0.46537
C	-6.03919	1.37176	-2.43018
H	-6.40211	2.34035	-2.79073
H	-5.12102	1.13828	-2.96661
H	-6.79561	0.63292	-2.71577

Conformer g1a_c31

C	-2.77302	-2.7694	-0.14177
C	-1.57425	-2.73049	0.56523
C	-0.94201	-1.51972	0.8487
C	-1.54128	-0.35296	0.36613
C	-2.7363	-0.35874	-0.34816
C	-3.35122	-1.59042	-0.58676
H	-3.2465	-3.72391	-0.34695
H	-1.05021	0.5948	0.55537
C	0.38433	-1.49281	1.60415
C	0.4029	-0.62052	2.84365
H	1.38963	-0.5284	3.2908
C	-0.62533	-0.03995	3.45076
H	-1.63609	-0.09324	3.06532
H	-0.47955	0.5124	4.37164
O	-0.95201	-3.89346	0.98796
H	-1.45064	-4.66217	0.68357
C	1.55724	-1.19782	0.66832
C	2.08489	0.09171	0.53542
C	2.138	-2.20986	-0.10107
C	3.14874	0.40787	-0.29712
H	1.64368	0.89024	1.12034
C	3.21421	-1.93321	-0.94658
C	3.7145	-0.64119	-1.04575
H	3.62938	-2.75026	-1.5187
O	1.72742	-3.51723	-0.08205
O	4.75733	-0.30005	-1.86101
C	5.38491	-1.32264	-2.62806
H	6.18272	-0.83221	-3.18136
H	5.81106	-2.09653	-1.98397
H	4.68242	-1.77875	-3.33082
H	0.87429	-3.63196	0.36893
H	0.54848	-2.50092	1.99754
C	3.70091	1.81761	-0.43596
H	4.79452	1.7495	-0.44052
H	3.43944	2.20694	-1.42363
C	3.28953	2.77329	0.64818
H	3.97104	2.81605	1.49605
C	2.22061	3.57525	0.68522
C	1.99457	4.50611	1.84973
H	2.7836	4.41978	2.59824
H	1.95349	5.54838	1.51435
H	1.03511	4.29871	2.33614
C	1.15968	3.6483	-0.38151
H	1.31288	2.9395	-1.19354
H	0.17232	3.45644	0.05191
H	1.11747	4.65431	-0.81316
H	-4.28802	-1.63038	-1.13066
C	-3.35316	0.93133	-0.86171
H	-3.39171	0.9031	-1.95278
H	-2.6823	1.75742	-0.60238
C	-4.71794	1.20717	-0.28531
H	-4.7044	1.78658	0.63601
C	-5.9116	0.83398	-0.75822
C	-7.17835	1.2179	-0.03584
H	-7.74696	0.32781	0.25513
H	-6.97297	1.80142	0.8627
H	-7.83504	1.80744	-0.68498
C	-6.13382	0.02876	-2.01154
H	-6.7289	0.6005	-2.73187
H	-5.21096	-0.27205	-2.50464
H	-6.70802	-0.87629	-1.78612

Conformer g1a_c15

C	-2.77302	-2.7694	-0.14177
C	-1.57425	-2.73049	0.56523
C	-0.94201	-1.51972	0.8487
C	-1.54128	-0.35296	0.36613
C	-2.7363	-0.35874	-0.34816
C	-3.35122	-1.59042	-0.58676
H	-3.2465	-3.72391	-0.34695
H	-1.05021	0.5948	0.55537
C	0.38433	-1.49281	1.60415
C	0.4029	-0.62052	2.84365
H	0.25208	0.43479	2.63001
C	0.64135	-0.98607	4.09761
H	0.80492	-2.01792	4.38323
H	0.68399	-0.24536	4.88748
O	-0.95201	-3.89346	0.98796
H	-1.45064	-4.66217	0.68357
C	1.55724	-1.19782	0.66832
C	2.08489	0.09171	0.53542
C	2.138	-2.20986	-0.10107
C	3.14874	0.40787	-0.29712
H	1.64368	0.89024	1.12034
C	3.21421	-1.93321	-0.94658
C	3.7145	-0.64119	-1.04575
H	3.62938	-2.75026	-1.5187
O	1.72742	-3.51723	-0.08205
O	4.75733	-0.30005	-1.86101
C	5.38491	-1.32264	-2.62806
H	6.18272	-0.83221	-3.18136
H	5.81106	-2.09653	-1.98397
H	4.68242	-1.77875	-3.33082
H	0.87429	-3.63196	0.36893
H	0.54848	-2.50092	1.99754
C	3.70091	1.81761	-0.43596
H	4.79452	1.7495	-0.44052
H	3.43944	2.20694	-1.42363
C	3.28953	2.77329	0.64818
H	3.97104	2.81605	1.49605
C	2.22061	3.57525	0.68522
C	1.99457	4.50611	1.84973
H	2.7836	4.41978	2.59824
H	1.95349	5.54838	1.51435
H	1.03511	4.29871	2.33614
C	1.15968	3.6483	-0.38151
H	1.31288	2.9395	-1.19354
H	0.17232	3.45644	0.05191
H	1.11747	4.65431	-0.81316
H	-4.28802	-1.63038	-1.13066
C	-3.35316	0.93133	-0.86171
H	-3.39171	0.9031	-1.95278
H	-2.6823	1.75742	-0.60238
C	-4.71794	1.20717	-0.28531
H	-4.7044	1.78658	0.63601
C	-5.9116	0.83398	-0.75822
C	-7.17835	1.2179	-0.03584
H	-7.74696	0.32781	0.25513
H	-6.97297	1.80142	0.8627
H	-7.83504	1.80744	-0.68498
C	-6.13382	0.02876	-2.01154
H	-6.7289	0.6005	-2.73187
H	-5.21096	-0.27205	-2.50464
H	-6.70802	-0.87629	-1.78612
